# Post-feeding transcriptomics reveals essential genes expressed in the midgut of the desert locust

**DOI:** 10.3389/fphys.2023.1232545

**Published:** 2023-08-10

**Authors:** Joachim Van Lommel, Michiel Holtof, Laurentijn Tilleman, Dorien Cools, Seppe Vansteenkiste, Daria Polgun, Rik Verdonck, Filip Van Nieuwerburgh, Jozef Vanden Broeck

**Affiliations:** ^1^ Molecular Developmental Physiology and Signal Transduction Lab, Department of Biology, University of Leuven, Leuven, Belgium; ^2^ NXTGNT, Department of Pharmaceutics, Ghent University, Ghent, Belgium; ^3^ Environmental Biology, Centre for Environmental Sciences, Hasselt University, Hasselt, Belgium

**Keywords:** post-feeding, transcriptomics, RNA-seq, digestion, desert locust, insect, Niemann-pick 1b protein, vacuolar-type H(+)-ATPase

## Abstract

The digestive tract constitutes an important interface between an animal’s internal and external environment. In insects, available gut transcriptome studies are mostly exploratory or look at changes upon infection or upon exposure to xenobiotics, mainly performed in species belonging to holometabolan orders, such as Diptera, Lepidoptera or Coleoptera. By contrast, studies focusing on gene expression changes after food uptake and during digestion are underrepresented. We have therefore compared the gene expression profiles in the midgut of the desert locust, *Schistocerca gregaria*, between three different time points after feeding, i.e., 24 h (no active digestion), 10 min (the initial stage of feeding), and 2 h (active food digestion). The observed gene expression profiles were consistent with the polyphagous herbivorous lifestyle of this hemimetabolan (orthopteran) species. Our study reveals the upregulation of 576 genes 2 h post-feeding. These are mostly predicted to be associated with digestive physiology, such as genes encoding putative digestive enzymes or nutrient transporters, as well as genes putatively involved in immunity or in xenobiotic metabolism. The 10 min time point represented an intermediate condition, suggesting that the *S. gregaria* midgut can react rapidly at the transcriptional level to the presence of food. Additionally, our study demonstrated the critical importance of two transcripts that exhibited a significant upregulation 2 h post-feeding: the vacuolar-type H(+)-ATPase and the sterol transporter Niemann-Pick 1b protein, which upon RNAi-induced knockdown resulted in a marked increase in mortality. Their vital role and accessibility via the midgut lumen may make the encoded proteins promising insecticidal target candidates, considering that the desert locust is infamous for its huge migrating swarms that can devastate the agricultural production in large areas of Northern Africa, the Middle East, and South Asia. In conclusion, the transcriptome datasets presented here will provide a useful and promising resource for studying the midgut physiology of *S. gregaria*, a socio-economically important pest species.

## 1 Introduction

The digestive tract represents an important interface between an animal’s external and internal environment. To ensure survival, changes in the digestive tract, such as food intake, infection, and ingestion of plant toxins and other xenobiotics, require a swift reaction. The aim of this study is to improve our understanding of insect digestive physiology by analyzing the midgut transcriptome of the socio-economically important pest species, the desert locust (*Schistocerca gregaria*) (Cullen et al., 2017), as well as to compare gene expression profiles in the midgut at different post-feeding time points.

Annotated midgut transcriptomes have become abundantly available, though mostly in holometabolan insect orders, such as Diptera, Lepidoptera and Coleoptera, presumably due to their large species diversity and prevalence of insect pests in these orders (Sanders et al., 2003; Campbell et al., 2005; Pauchet et al., 2009; Ma et al., 2012; Xu et al., 2012; Noland et al., 2013; Herde and Howe, 2014; Spit et al., 2016a; Zhang et al., 2016; Gazara et al., 2017; Huang et al., 2017; Nadeau et al., 2017; Lu et al., 2018; Dhania et al., 2019; Bonelli et al., 2020; Coutinho-Abreu et al., 2020; Cui and Franz, 2020; Hung et al., 2020; Kuang et al., 2021; Shu et al., 2021; Zou et al., 2021; Hixson et al., 2022; Jin et al., 2022). As expected in the insect midgut, transcripts encoding digestive enzymes, nutrient transporters, and proteins involved in detoxification and peritrophic membrane formation are abundantly present (Pauchet et al., 2009; Ma et al., 2012; Gazara et al., 2017). The composition of midgut digestive enzymes is often complex, species-dependent and is largely determined by feeding habits, food quality, and specific midgut luminal environments, and presence of symbiotic microbiota (Holtof et al., 2019; Hung et al., 2020).

In addition to descriptive transcriptomic studies, many comparative studies are available, primarily focusing on changes in midgut gene expression upon infection or exposure to xenobiotics. Changes in immunity- and detoxification-related genes upon infection tend to be complex as well as species- and pathogen- or toxin-dependent. Upon infection, a shift in gene expression generally occurs, inhibiting digestive processes in favor of the immune system (Noland et al., 2013; Coutinho-Abreu et al., 2020; Kuang et al., 2021). Transcriptomic studies investigating the xenobiotic metabolism in the digestive tract clearly revealed a strong ability of insects to metabolize foodborne toxins, i.e., plant-derived xenobiotics, bacterial entomotoxins, or synthetic insecticides (Herde and Howe, 2014; Dhania et al., 2019; Shu et al., 2021; Jin et al., 2022). This is reflected by high numbers of detoxification enzymes, namely, cytochrome P450s (CYPs), carboxylesterases (CEs), glutathione S-transferases (GSTs), and uridine diphosphate glucuronosyl-transferases (UGTs), being observed in the insect midgut (Pauchet et al., 2009; Spit et al., 2016a; Zhang et al., 2016; Rane et al., 2019).

Despite the abundance of midgut transcriptomes, very few studies have focused on post-feeding midgut gene expression changes. Often these studies have focused on gene expression changes upon a bloodmeal in dipterans, where generally an upregulation of digestion-related genes is reported, especially of serine proteases, which is to be expected for a protein-rich food source such as blood (Sanders et al., 2003; Campbell et al., 2005; Cui and Franz, 2020). There is a limited number of reports comparing high-quality to low-quality diets in herbivorous insects (Huang et al., 2017; Lu et al., 2018; Bonelli et al., 2020; Zou et al., 2021). In *Chilo suppressalis* larvae fed on a toxin-rich plant diet, the majority of studied digestion-, detoxification-, and immune-related genes were downregulated (Lu et al., 2018). By contrast, when *Spodoptera litura* larvae were fed with a toxin-rich food source, upregulated genes were enriched in detoxification-related genes as soon as 6 h after feeding, while downregulated genes were enriched in digestion- and immune-related genes as soon as 48 h after feeding (Zou et al., 2021). Various digestive enzymes were upregulated when the grasshopper, *Oedaleus asiaticus*, was fed on a high-quality food source (Huang et al., 2017). Black soldier fly (*Hermetia illucens*) larvae showed the ability to optimally exploit various food sources by tweaking the expression of digestive enzymes (Bonelli et al., 2020). Nevertheless, research investigating post-feeding temporal changes in midgut gene expression, besides post-bloodmeal ingestion, are surprisingly scarce. In *Nilaparvata lugens*, two midgut sugar transporters were found to be upregulated 2 h after feeding, although other genes were not analyzed (Kikuta et al., 2015). One study investigated differences between unfed and fed males and females in the beetle *Dendroctonus ponderosae.* Although primarily focusing on pheromone biosynthesis (Nadeau et al., 2017), large differences were observed in gene expression profiles, including an upregulation of digestion related genes upon feeding. In *Bombyx mori*, many digestion-related genes were reported to be downregulated, while immunity-related genes were upregulated in the wandering stage, during which larvae stop feeding (Xu et al., 2012).

Here, we intend to address this knowledge gap by investigating the gene expression changes upon feeding in the midgut of the desert locust, *S. gregaria* (Orthoptera). The desert locust is a polyphagous herbivorous species, known as one of the most destructive migratory pests in the world due to its swarming behavior and voracious appetite (Cullen et al., 2017). Gut transit time can vary greatly amongst insects, depending on feeding habitats and diet (Chapman, 2013). Locusts will ingest food until their crop is maximally expanded, resulting in the activation of stretch receptors present on the foregut (Chapman, 2013). Active stretch receptors will lead to the cessation of feeding. Water and solutes are absorbed in the midgut of *Locusta migratoria* as soon as 5 min after feeding, while solid particles enter the midgut as soon as 15 min after feeding (Baines et al., 1973). In *L. migratoria* with continuous access to food a meal had left the crop and had entered the midgut after 1.5 h and the midgut was empty after 2 h, which is consistent with observations in the grasshopper *Abracris flavolineata* (Baines et al., 1973; Simpson, 1983; Marana et al., 1997). By contrast, in the absence of additional food, it can take 8 h for food to leave the locust midgut (Chapman, 2013).

The main objective of this study is to analyze the general midgut transcript profile of *S. gregaria* as well as changes in midgut gene expression during different stages of feeding. By doing so we aim to address the underrepresentation of midgut gene expression studies outside of the Diptera, Lepidoptera and Coleoptera orders as well as the lack of studies focusing on post-feeding temporal changes. To illustrate this, there are currently only two Orthoptera species with a publicly available annotated midgut transcriptome: *L. migratoria* and *O. asiaticus* (Spit et al., 2016a; Huang et al., 2017). This is remarkable as some orthopteran species, including *S. gregaria*, are notorious pests*.* In this study, we compared the midgut gene expression profile between three different time points of the digestive process, which were chosen based on reports in literature (Baines et al., 1973; Marana et al., 1997) as well as our own observations: 24 h after feeding (no active digestion), 10 min after feeding (the initial stages of feeding) and 2 h after feeding (active digestion in the midgut). In addition, we functionally characterized two transcripts that were upregulated upon feeding, using RNA interference, namely, a vacuolar-type H(+)-ATPase (H^+^ V-ATPase) subunit (*SgVAHa1*) and a sterol transporter orthologue (*SgNPC1b*).

## 2 Materials and methods

### 2.1 Animal rearing

Desert locusts (*S. gregaria gregaria*) were kept under crowded conditions at a controlled temperature (30°C ± 1°C), relative humidity (50% ± 10%), and photoperiod (a daily 14 h:10 h light:dark cycle). In addition, during this photoperiod, incandescent light bulbs (40 W) generated a temperature gradient within the cages. Locusts were allowed daily to feed *ad libitum* on fresh cabbage leaves (*Brassica oleracea*), supplemented with rolled oats. Adult females deposited their eggs in pots filled with a slightly moistened turf/sand mixture (1:3 ratio). Pots were collected weekly and then transferred to empty cages. Measures were taken to avoid contamination of midgut samples intended for sequencing by gregarines (2.3). Gregarines are parasitic microorganisms that infect the midgut of *S. gregaria* and other Orthoptera. Six to seven-day-old eggs were carefully removed from the egg pods and rinsed with distilled water to remove soil particles. Next, eggs were disinfected by washing them in ethanol (70%) for 30 s, then rinsed with distilled water and finally transferred to clean egg pots (pretreated with 10% bleach) containing an autoclaved turf/sand mixture. The egg pots were placed in a disinfected cage (pretreated with 10% bleach) located inside a closed incubator (J.P. Selecta^®^, 30°C ± 1°C, relative humidity 50% ± 10%).

### 2.2 Tissue collection and RNA extraction

#### 2.2.1 Dissections and tissue collection

All dissections were performed under an SZ2-ST binocular microscope (Olympus), and images were taken using a mounted HD-Pro VC.3038 camera (Euromex). Animals were weighed using a Genius ME215p analytical balance (Sartorius). Tissues were immediately transferred to screwcapped 2 mL Eppendorf vials containing 0.5 mL RNAlater (Thermo Scientific™) to avoid RNA degradation. Subsequently, tissues were removed from RNAlater, rinsed in *S. gregaria* Ringer (1 L: 8.766 g NaCl; 0.188 g CaCl_2_; 0.746 g KCl; 0.407 g MgCl_2_; 0.336 g NaHCO_3_; 30.807 g sucrose; 1.892 g trehalose; pH 7.2), and transferred to MagNA Lyser Green Beads vials (Roche), with the exception of suboesophageal ganglia (SOG) and prothoracic glands (PG) which were transferred to screwcapped 1.5 mL Eppendorf vials. All tissues were stored at −80°C until RNA extraction (2.2.2). For ecdysteroid measurements, 10 µL of hemolymph was collected using a glass capillary prior to sacrificing the animals and immediately transferred to 300 µL of phosphate-buffered saline (PBS) (13.7 mM NaCl, 0.27 mM KCl, 1 mM NH_2_HPO_4_, 0.18 mM KH_2_PO_4_, pH 7.4) and 300 µL of 1-butanol (Merck). After vortexing and centrifugation at 20,000 × g for 10 min, the upper organic phase was collected, lyophilized using a SpeedVac vacuum concentrator, and stored at −20°C until ecdysteroid extraction (2.6).

#### 2.2.2 Total RNA extraction

Two different RNA extraction methods were used based on tissue type. Total RNA was extracted using the RNeasy Lipid Tissue extraction kit (Qiagen) according to the manufacturer’s protocols. To each MagNA Lyser Green Beads vial (Roche), 1 mL QIAzol lysis reagent buffer (Thermo Scientific™) was added. Next, the tissues were homogenized for 30 s at 6,000 × g using a MagNA Lyser instrument (Roche). Finally, total RNA was eluted in 80 μL of nuclease-free water. By contrast, total RNA was extracted from the SOG and PG using the RNAqueous®-Micro Kit (Ambion) according to the manufacturer’s protocols. First, 100 µL of lysis solution was added to the tissues. Next, tissues were disrupted and homogenized using a Teflon micro pestle attached to a drill. Using either extraction method, a DNase treatment was performed upon elution of the extracted RNA. In both cases, the concentration of the resulting RNA extracts was determined by means of a Nanodrop spectrophotometer (Nanodrop ND-1000, Thermo Fisher Scientific, Inc.). RNA extracts were stored at −80°C.

### 2.3 mRNA sequencing of the midgut

The *S. gregaria* midgut transcriptome was studied at three distinct time points. The following steps were therefore followed. First, the whole-body transcriptome of Ragionieri et al. (Ragionieri et al., 2022) (GenBank accession number GJPN000000000.1) was cleaned and annotated (2.3.1). Next, total RNA extracts from the midgut (2.3.2) were sequenced and mapped (2.3.3) to this reference transcriptome. Finally, a differential gene expression analysis was performed to characterize gene expression changes across three different timepoints of the digestive process (2.3.4).

#### 2.3.1 Processing and annotating of the reference transcriptome

**FIGURE 1:**
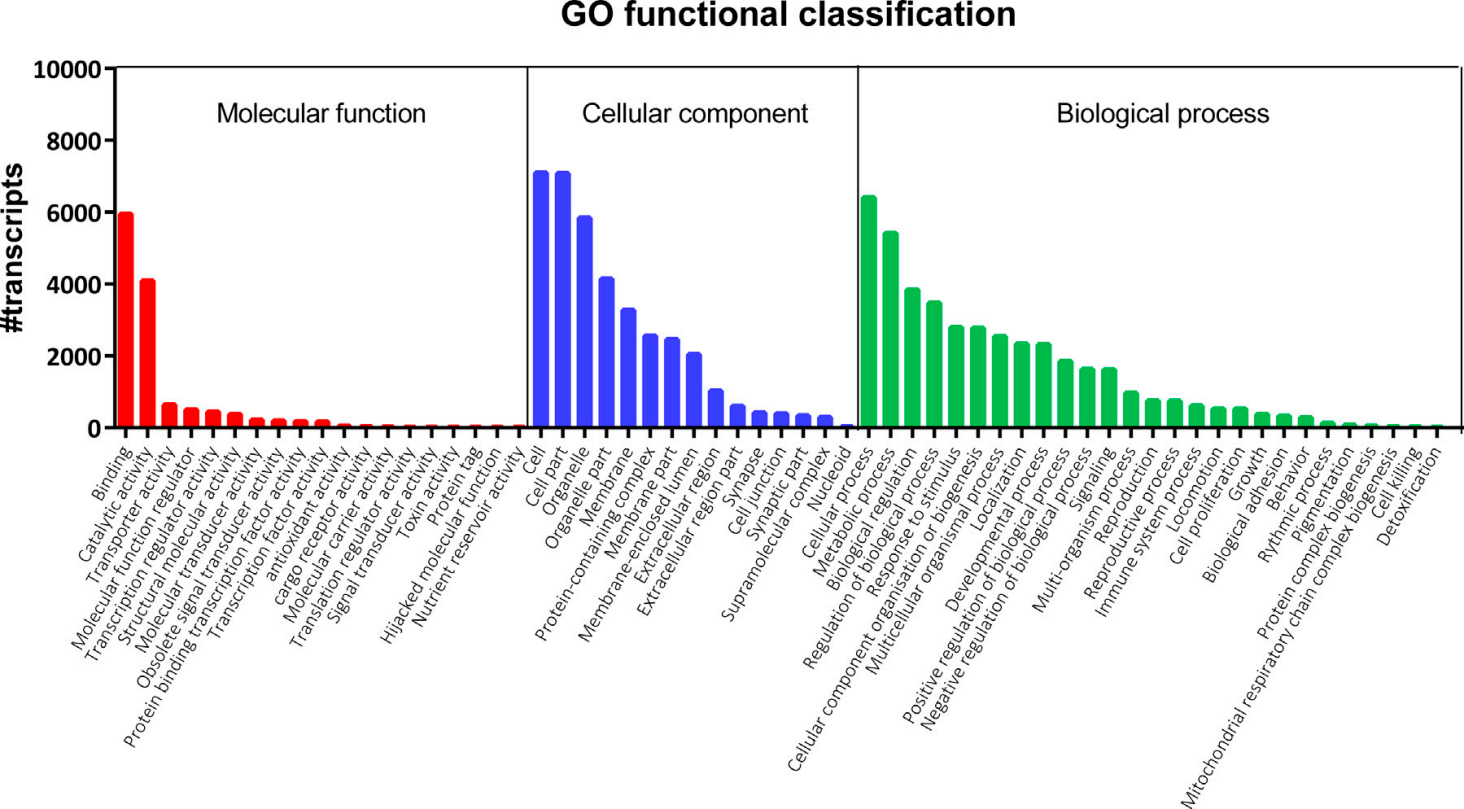
Gene Ontology (GO) classification of nucleotide sequences in the *S. gregaria* midgut transcriptome. GO terms belonging to three categories are indicated on the *x*-axis: “Molecular function” (red), “Cellular component” (blue), and “Biological process” (green). The number of annotated transcripts is indicated on the *y*-axis. A total of 22,961 GO terms were assigned to 8,645 distinct transcripts (44.6%) of the midgut transcriptome. These GO terms were further subdivided into 7,443; 7,837 and 7,681 terms representing “Biological process”, “Cellular component”, and “Molecular function”, respectively.

**FIGURE 2:**
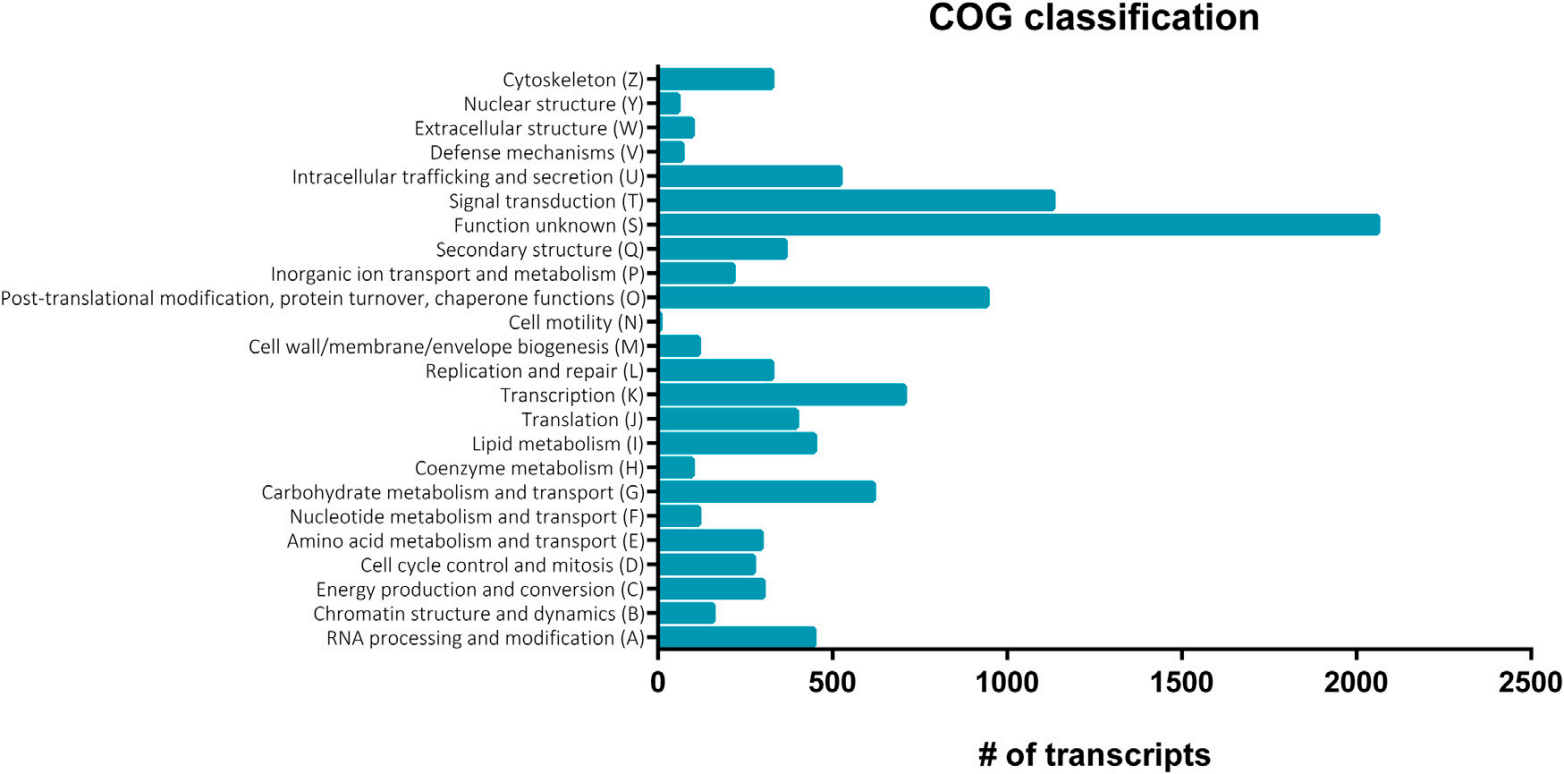
Clusters of orthologous groups (COG) functional classification of nucleotide sequences in the *S. gregaria* midgut transcriptome. COG assignments were based on the COG alphabet, retrieved from the Swiss-Prot database and used to cluster transcripts according to predicted orthology. A total of 7,142 transcripts (36.9%) of the midgut transcriptome could be attributed to at least one COG category. The number of transcripts is plotted on the *x*-axis. The different COG categories are shown on the *y*-axis.

The *S. gregaria* whole-body transcriptome of Ragionieri et al. (Ragionieri et al., 2022) (GenBank accession number GJPN000000000.1) contains different gene isoforms and was therefore first processed to reduce the redundancy of transcript sequences. The tool Supertranscripts combines all isoforms of a transcript into one assembled contiguous sequence referred to as a SuperTranscript (Davidson et al., 2017). A SuperTranscript is a single linear sequence constructed from collapsing unique and common sequence regions of all available splicing isoforms of a transcript. This way, all isoforms of a transcript are combined into one assembled contiguous sequence without losing transcript data (Davidson et al., 2017). Furthermore, the original transcripts that make up the SuperTranscripts can be retraced in the original transcriptome (GJPN000000000.1). Therefore, the SuperTranscripts transcriptome version provides a very potent alternative reference for mapping RNA-Seq reads. The SuperTranscripts transcriptome retained 140,093 distinct transcripts. Next, the CD-HIT-EST tool clusters highly similar sequences (>95%) and thereby filters remaining redundant sequences, resulting in the removal of 16,379 redundant contigs (Li and Godzik, 2006; Fu et al., 2012). The resulting processed transcriptome containing 123,714 contigs was submitted to Gene Expression Omnibus (GEO) (accession number GSE226871). Next, the processed transcriptome was annotated in different steps. First, these nucleotide sequences were translated to their predicted best coding sequence using Transdecoder^1^ and annotated using Trinotate according to the procedure described by Bryant et al*.* (Bryant et al., 2017). Next, all transcript sequences and their predicted coding sequences were used as queries for Basic Local Alignment Search Tool searches (BLASTx and BLASTp, E ≤ 1e^−5^) (Boratyn et al., 2013) in the annotated UniProtKB/Swiss-Prot databases (Boeckmann et al., 2005; Bateman et al., 2015). The hits from these homology searches were used to provide the functional annotations of Gene Ontology (GO) and Clusters of Orthologous Groups (COG) terms (Figures 1, 2). The predicted coding regions were further used to annotate domain content in the Pfam database currently hosted by InterPro^2^ (Mistry et al., 2021) using the HMMER software^3^ (version 3.1.2), signal peptides^4^ (SignalP, version 4.1), and transmembrane domains^5^ (TMHMM, version 2.0). This processed and annotated transcriptome served as a reference for mapping the sequenced reads and is referred to as the reference transcriptome from this point forward. Finally, the annotated mapped midgut transcripts were organized in an SQLite database using Trinotate (Supplementary Table S1).

#### 2.3.2 Sample preparation

**FIGURE 3:**
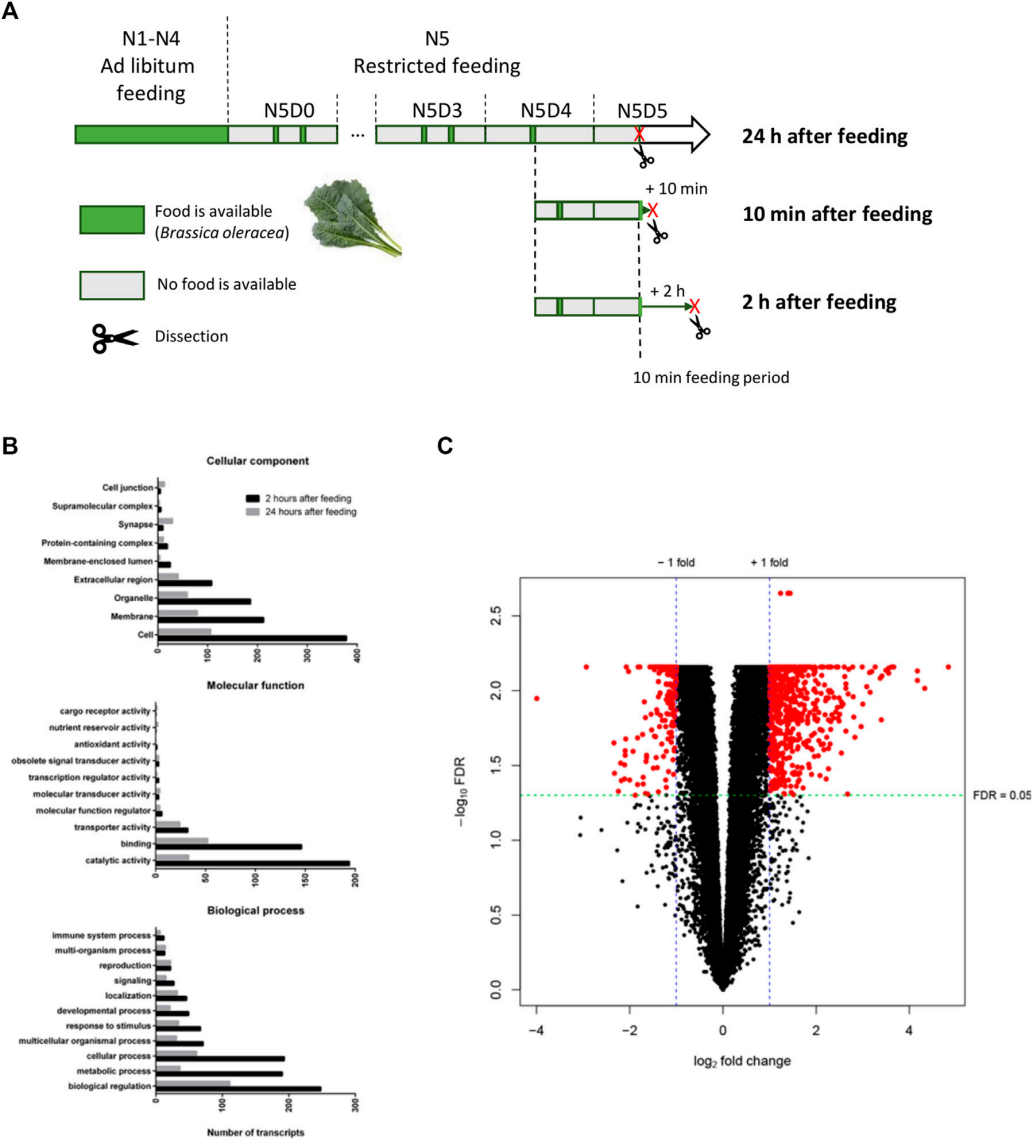
Differential gene expression analysis of the midgut transcriptome. **(A)** Graphical overview of the sampling strategy for the collection of midgut tissues at three post-feeding timepoints for RNA-seq. Desert locusts (*Schistocerca gregaria*) were fed *ad libitum* from the first (N1) until the end of the 4th nymphal instar (N4). From the day animals molted to the 5th nymphal instar (N5D0) they were fed twice a day during 1 h feeding periods at 10 a.m. and 5 p.m. (restricted feeding). Feeding periods when animals were provided *Brassica oleracea* leaves are indicated by green bars. Periods when animals were deprived of food are indicated by grey bars. The length of the bars is time-proportional for the N5 instar. Sacrificing and subsequent dissection of animals are indicated by a red “X” and a scissor pictogram, respectively. Animals were divided into three groups on the morning of N5D4. Animals from the 24 h condition were deprived of food after the feeding session on the morning of N5D4 and dissected 24 h later on N5D5. Animals from the remaining two conditions were allowed to feed for 10 min on the morning of N5D5 and were dissected 10 min or 2 h after this initial feeding period (10 min after feeding and 2 h after feeding condition, respectively). **(B)** Summary of the gene ontology (GO) term annotations of differentially expressed genes in the *S. gregaria* midgut 2 hours after feeding (2 h) compared to 24 h after feeding (24 h). Black bars represent transcripts belonging to GO terms that are upregulated 2 h after feeding compared to 24 h, while gray bars represent transcripts belonging to GO terms that are downregulated 2 h after feeding compared to 24 h. GO terms are subdivided into three ontologies: “Cellular component”, “Molecular function”, and “Biological process”. GO terms are indicated on the *y*-axis. The number of annotated transcripts is indicated on the *x*-axis. **(C)** Volcano plot of differentially expressed genes 2 h after feeding (2 h) compared to 24 h after feeding (24 h). For each individual transcript (represented by a single dot) the negative log of the false discovery rate adjusted *p*-values (-log_10_FDR) is plotted against the log_2_ fold change (log_2_FC). Red dots represent differentially expressed transcripts. A -log_10_FDR-adjusted *p*-value of ±1.30 corresponds to an FDR-adjusted *p*-value (FDR) of 0.05. 569 transcripts with log_2_FC ≥ 1 and FDR ≤ 0.05 were considered upregulated 2 h after feeding compared to 24 h. 212 transcripts with log_2_FC ≤ −1 and FDR ≤0.05 were considered downregulated 2 h after feeding compared to 24 h.

Three different post-feeding timepoints were selected based on the progression of the digestive process in *S. gregaria*. Based on descriptions in literature (Baines et al., 1973; Marana et al., 1997) and our own observations, we decided to analyze the transcript profile of the midgut in *S. gregaria* during the initial phase of digestion (10 min after food uptake) when the ingested food was present in the foregut, and the core phase of digestion when the majority of the food bolus was observed in the midgut lumen (2 h after food uptake). We compared both these timepoints to a reference condition with locusts that had not been fed for 24 h (24 h after food uptake). This condition can therefore also be considered as a group of animals that were food-deprived (or starved) for 24 h. To obtain samples from these timepoints, animals with a balanced sex ratio were bred under gregarine-free conditions (2.1). From the day they molted to the 5th nymphal stage (N5D0), feeding was synchronized by allowing animals to feed on freshly washed cabbage leaves twice daily, in the morning and the afternoon, for 1 h periods (Figure 3A). After feeding on the morning of the 4^th^ day of the N5 stage (N5D4), three groups were established: one group was deprived of food for the next 24 h and sacrificed the next day (24 h condition). The other animals were allowed to eat for 1 h in the afternoon of N5D4 and received a final meal in the morning of N5D5. They were allowed to eat for 10 min from this meal, which was equal to their typically observed initial feeding stage. A group of animals was sacrificed after an additional 10 min (10 min condition), and a second group of animals was sacrificed after 2 h (2 h condition). In both cases, the time was measured from the end of the initial 10 minutes feeding period. Animals were dissected as described in 2.2.1. Additionally, the dissections of all three groups were executed on the same day around the same time to exclude any potential circadian effects. Midgut tissues of six animals were pooled per sample. For each condition, six biological replicate samples were collected.

#### 2.3.3 Mapping of sequenced reads to the reference transcriptome

From each sample, 1 μg of total RNA was used to perform an Illumina^®^ sequencing library preparation using the TruSeq^®^ Stranded mRNA Library prep kit (Illumina^®^) according to the manufacturer’s protocol. First, poly-A-containing mRNA molecules are enriched from the total RNA mixture using poly-dT oligonucleotides attached to magnetic beads. Next, these purified mRNA molecules were fragmented into smaller fragments (100–1,000 nt) using divalent metal cations under elevated temperature in a proprietary Illumina^®^ fragmentation buffer. The smaller RNA fragments resulting from the fragmentation step were used for the first-strand cDNA synthesis using reverse transcriptase and random primers. Next, a single adenosine was added to these cDNA fragments, followed by the ligation of the Illumina^®^ adapters. The resulting cDNA products were then purified and enriched with 13 PCR cycles to create the final cDNA library. Libraries were quantified by RT-qPCR, according to Illumina’s “Sequencing Library qPCR Quantification protocol guide,” version February 2011. A High Sensitivity DNA chip (Agilent Technologies^®^) was used to control the library’s size distribution and quality. A total of six midgut samples per condition (eighteen in total) were sequenced on a high throughput Illumina^®^ NextSeq 500 flow cell generating 75 bp single reads. The samples were multiplexed, allowing for the sequencing of all samples on one Illumina^®^ flow cell. Moreover, sequencing was performed in duplicate on two different flow cells to increase sequencing depth (Supplementary Figure S1).

Prior to mapping, Illumina^®^ adapter sequences were trimmed using Cutadapt (version 1.11) (Martin, 2011). Base calling quality was assessed using FastQC^6^. Trimmed sequencing reads were mapped to the reference transcriptome using Bowtie2 (version 2.2.5). Transcript abundance was quantified using the RSEM software package (RNA-seq by Expectation Maximization, version 1.2.31) (Li and Dewey, 2011). Transcripts that did not have an abundance of at least 1 count-per-million in at least six out of eighteen samples were considered to be both biologically and statistically irrelevant and were removed from the dataset prior to downstream analyses. The other transcripts were considered quantifiable. Read counts were normalized using Bioconductor software edgeR’s standard TMM normalization method (Robinson, McCarthy, and Smyth, 2009) in R, version 3.4.2^7^. These TMM-normalized transcript counts were used to calculate differential expression among the three conditions (Supplementary Table S2).

#### 2.3.4 Differential expression analysis

A principal component analysis (PCA) was performed on regularized log-transformed counts using the DESeq2 Bioconductor software in R (Supplementary Figure S2) (Love et al., 2014). Differential expression analysis between groups of samples was performed using the Bioconductor package edgeR in R (Robinson et al., 2009). Three separate differential expression analyses were performed: 10 min vs. 24 h, 2 h vs. 24 h, and 10 min vs. 2 h. A generalized linear model was built for each separate analysis, and statistical testing was done using the empirical Bayes quasi-likelihood F-test. Transcripts having a false discovery rate (FDR) adjusted *p*-value ≤ 0.05 and a log_2_ fold change (FC) ≥ 1 or ≤ −1 were considered to be significantly differentially expressed.

### 2.4 RNA interference

Double-stranded (ds)RNA constructs targeting *SgNPC1b* (*dsNPC1b1* and *dsNPC1b2*), *SgVAHa1* (*dsVAHa1*) and *GFP* (*dsGFP*) were produced using the MEGAscript^®^ RNAi kit (Ambion). This procedure of dsRNA production is based on a high-yield *in vitro* transcription reaction from a DNA template with flanking T7 promoter sequences. Therefore, forward and reverse primers flanked by the T7 promoter sequence were used in a PCR reaction with REDTaq DNA polymerase (Sigma-Aldrich) to amplify a fragment of the target gene (Supplementary Table S3). Primer positions and amplicon sequences can be found in Supplementary Figures S3, S4. The dsRNA constructs are 542 bp (*dsVAHa1*), 795 bp (*dsNPC1b1*), 550 bp (*dsNPC1b2*) and 589 bp (*dsGFP*) in length. Amplicons were separated by agarose gel electrophoresis (1.2% agarose gel containing GelRedTM (Biotium) and further purified (GenElute^TM^ Gel Extraction Kit, Sigma-Aldrich). These fragments were cloned (TOPO^®^ TA cloning kit for sequencing, Invitrogen) and the amplicon sequences were confirmed (Sanger sequencing, LGC genomics, Berlin, Germany). The resulting plasmids were subsequently used as templates for *in vitro* transcription. The dsRNA concentration was determined by means of a Nanodrop spectrophotometer (Nanodrop ND-1000, Thermo Fisher Scientific, Inc.). The dsRNA was further diluted to 50 ng/μL in an isotonic *S. gregaria* Ringer solution to avoid osmotic effects upon injection and stored at −20°C. Locusts were injected with dsRNA using a Hamilton syringe into the hemocoel by entering the needle between the first and second abdominal segment on the ventrolateral side, pointing towards the thorax. Animals were marked on their prothoracic tergite with nail polish to distinguish the experimental conditions. Animals were injected on N4D6, N5D1, N5D3, and N5D6 with 500 ng of dsRNA in 10 µL of *S. gregaria* Ringer solution (*SgVAHa1* KD, *dsGFP*: *n* = 45, *dsVAHa1*: *n* = 46; *SgNPC1b* KD, *dsGFP*: *n* = 10, *dsNPC1b1*: *n* = 10, *dsNPC1b2*: *n* = 10). A subset of fifteen animals per condition were dissected on N5D6 for their midgut to assess knockdown efficiency. The remaining animals were monitored daily to assess mortality and successful molting until they either died or molted into adulthood. In case of a *SgNPC1b* knockdown, animals were weighed on N5D0 and N5D6.

### 2.5 RT-qPCR

Prior to RT-qPCR transcript profiling, several previously described housekeeping genes were tested for their stability (Van Hiel et al., 2009). Optimal housekeeping genes were selected using the geNorm software (Vandesompele et al., 2002). RT-qPCR primers for reference genes and target genes were designed using the online tool primer3plus^8^ (Supplementary Table S4). Primer sets were validated by designing relative standard curves for gene transcripts with a serial ten-fold dilution of a calibrator cDNA sample. The efficiency of RT-qPCR and correlation coefficient were measured for each primer pair. All PCR reactions were performed in duplicate in 96-well plates on a QuantStudio™ 3 Real-Time PCR System (ABI Prism, Applied Biosystems). Each reaction contained 5 µL Fast SYBR^TM^ Green Master Mix (Applied Biosystems), 0.5 µL Forward and Reverse primer (10 μM), 1.5 µL Milli-Q water (Millipore, MQ), and 2.5 µL of diluted cDNA. For all RT-qPCR reactions, the following thermal cycling profile was used: 50°C for 2 min, 95°C for 10 min, followed by 40 cycles of 95°C for 15 s and 60°C for 60 s. Finally, a melt curve analysis was performed to check for primer dimers. Relative transcript levels were calculated using the ΔΔCt method according to Livak and Schmittgen (Livak and Schmittgen, 2001). To correct for variation between samples, expression was normalized against two housekeeping genes, *SgRP49* and *SgGAPDH*.

### 2.6 Ecdysteroid measurements using EIA

Hemolymph samples were collected, and the organic phase was stored as described in 2.2.1. Dried extracts were dissolved in 200 µL chloroform (Acros Organics, Thermo Fisher Scientific) and 200 µL MQ. The organic phase was re-extracted twice with (1:1) MQ. Next, the aqueous phases were pooled and lyophilized. Ecdysteroid titers were measured using the Enzyme Immunoassay kit (Bertin Bioreagent) according to the manufacturer’s protocol. This protocol includes acetylcholinesterase (AChE^®^)-labeled 20-hydroxyecdysone (20E) as a tracer together with rabbit polyclonal antiserum. Absorbance was measured at 414 nm using a Mithras LB 940 (Mikrotek) microplate reader. The ecdysteroid levels were normalized to a series of known 20E concentrations ranging from 39.1 pg/mL to 5,000 pg/mL.

### 2.7 Statistical analysis

Differential expression analysis of the midgut transcriptome is described in 2.3.4. Relative transcript levels, weight gain, and 20E levels were first tested for normality using the Shapiro-Wilk normality test. Next, differences between control and treatment groups were inferred using the Student’s t-test or one-way ANOVA with Holm-Šídák’s *post hoc* test. Survival and molting rate were analyzed using a log-rank Mantel-Cox test. The threshold for statistical significance was *p* ≤ 0.05. All of the above-mentioned statistical analyses were performed using GraphPad Prism software (Version 6).

## 3 Results

### 3.1 mRNA sequencing of the midgut

A total of eighteen samples, six per timepoint, were sequenced on two Illumina^®^ NextSeq 500 flow cells, generating on average 55 ± 10 million (M) reads per sample. On average, 88%–90% of the reads mapped at least once to the reference transcriptome. RSEM was used to estimate the transcript abundances in all eighteen RNA-Seq samples (Supplementary Table S2). These data were used to infer differential gene expression across the different conditions (3.3). Amongst 19,345 quantifiable transcripts, 5,732 are SuperTranscripts constructed from at least two transcript isoforms, while the remaining 13,613 are unique transcripts. Using Transdecoder, 10,536 transcripts were shown to have at least one open reading frame. Trinotate retrieved GO and COG based functional annotations of the transcripts from the Swiss-Prot database (Table 1). Altogether, a total of 10,939 of the quantifiable transcripts had at least one functional annotation. Additionally, non-coding (nc) RNAs databases were searched, but no significant hits were retrieved. A total of 22,961 GO terms were assigned to 8,645 distinct transcripts (44.6%). These GO terms were further divided into 7,443; 7,837 and 7,681 terms representing biological process (BP), cellular component (CC), and molecular function (MF), respectively (Figure 1). The BP ontology indicated that the annotated transcripts are predicted to mediate a wide range of biological processes, including cellular, metabolic, and regulatory processes. In the CC ontology, the most abundant subcategories were “cell” and “cell part” (7,098 and 7,079 transcripts; respectively). Additionally, a high number of transcripts were assigned to the subcategories “membrane” (3,274 transcripts) and “extracellular region” (1,022 transcripts). Most transcripts in the MF ontology, 5,947 and 4,090; respectively, were assigned to the subcategories “binding” and “catalytic activity.” A total of 7,142 transcripts (36.9%) could be attributed to at least one COG category (Figure 2). “Function unknown” (S) was by far the largest represented COG group (2,060; 20.5%), followed by “signal transduction” (T) (1,130; 11.2%), and “post-translational modification, protein turnover and chaperone functions,” respectively (O) (941, 9.3%).

**TABLE 1:** Functional annotation of the *S. gregaria* midgut transcriptome by matching to different databases.

Annotation	# Transcripts
BLASTx UniProtKB/Swiss-Prot	8,659
BLASTp UniProtKB/Swiss-Prot	7,990
Pfam	7,656
GO	8,645
COG	7,142

### 3.2 Composition of the midgut transcriptome

#### 3.2.1 Digestive enzymes

Numerous transcripts predicted to encode proteases were identified amongst putative digestive enzymes, of which the majority code for serine proteases, such as trypsin and chymotrypsin (Supplementary Table S5). Large numbers of the most commonly found types of carbohydrases were identified, namely, α-Amylase, α-Glucosidase, and β-Glucosidase, as well as three transcripts encoding putative cellulases belonging to the GH9 family of endoglucanases (Pfam00759). A relatively large variety of lipid-degrading enzymes was identified, namely, lipases, phospholipases, and sterol dehydrogenases/reductases. Finally, putative DNase, RNase, nucleotidase and nucleosidase encoding transcripts were present in the *S. gregaria* midgut.

#### 3.2.2 Nutrient absorption and other intestinal processes

Multiple transcripts encoding putative amino acid, carbohydrate, and lipid transporters were identified (Supplementary Table S6). Additionally, the midgut transcriptome was scanned for other transcripts known to contribute to the intestinal physiology of herbivorous insects, including those involved in peritrophic membrane formation, transmembrane trafficking, and xenobiotic metabolism (Supplementary Table S7). Transcripts predicted to encode proteins active in all three phases of detoxification of xenobiotics were identified, namely, P450s and CEs (phase I), UGTs and GSTs (phase II) as well as putative transmembrane transporters such as members of the ATP-binding cassette (ABC) family of transporters (phase III).

### 3.3 Differential expression analysis

A PCA was performed to explore if samples clustered condition-dependently and to detect any outlier samples (Supplementary Figure S2). The majority of the variability, 48% and 17.7% respectively, could be explained by principal components (PC) 1 and 2. Samples of the 2 h and 24 h conditions could be separated on PC1, with the exception of sample sixteen. By contrast, two samples of the 10 min condition grouped with the 24 h condition, while the remaining samples grouped with the 2 h condition. A differential gene expression analysis was performed with pairwise comparisons of all three conditions. No significantly differentially expressed genes (DEGs) were identified when comparing the 10 min condition to the 24 h condition or the 10 min condition to the 2 h condition. Nevertheless, a total of 569 and 212 transcripts were found to be significantly up- and downregulated, respectively, in the 2 h condition compared to the 24 h condition (Figure 3; Supplementary Tables S8, S9). From these transcripts, 283 up- and 84 downregulated transcripts had at least one GO term annotation, meaning that 50.3% and 60.4% of these transcripts, respectively, did not have a functional annotation. Transcripts with a GO term annotation that were upregulated 2 h post-feeding compared to 24 h, belong mostly to the GO terms “catalytic activity” and “binding” from the MF ontology and “Cellular process”, “Metabolic process”, and “Biological regulation” from the BP ontology (Figure 3B).

Differentially expressed transcripts 2 h after feeding were classified according to Pfam protein family, of which 239 up- and 69 downregulated transcripts could be ascribed to at least one Pfam family. Amongst others, various transcripts predicted to encode proteins contributing to the enzymatic digestion, nutrient uptake and detoxification of xenobiotics were upregulated 2 h after feeding (Table 2). In contrast to the high number of induced putative protease and carbohydrase encoding transcripts, significantly fewer putative lipid-degrading enzymes were induced in transcript levels 2 h after feeding. Furthermore, transcripts predicted to encode transporters for all major nutrients, namely, amino acids, carbohydrates, and lipids, were represented amongst upregulated transcripts. We also identified transcripts encoding proteins that are predicted to play a role in all three phases of detoxification of xenobiotics, namely, CEs, GSTs, UGTs, nitrile-specifier proteins (NSP) and members of the ABC family of transporters. In addition to putative ABC transporters, transcripts predicted to encode other transmembrane protein complexes were upregulated, such as a H^+^ V-ATPase subunit-a transcript. By contrast, according to their Pfam classification, no genes predicted to be involved in enzymatic digestion or nutrient uptake were downregulated 2 h after feeding (Table 3).

**TABLE 2:** Summary of Pfam families of upregulated transcripts 2 h after feeding versus 24 h after feeding.

Classification	Pfam	# DEGs	Description
Peritrophins	PF01607	17	Chitin binding Peritrophin-A domain
Enzymatic digestion	PF00089	20	Trypsin
	PF00232	15	Glycosyl hydrolase family 1
	PF01055	8	Glycosyl hydrolases family 31
	PF00151	5	Lipase
	PF01433	3	Peptidase family M1 domain
	PF11838	3	ERAP1-like C-terminal domain - Aminopeptidase family
	PF05577	1	Serine carboxypeptidase S28
	PF00128	1	Alpha amylase, catalytic domain
	PF02449	1	Beta-galactosidase
	PF00686	1	Starch binding molecule
	PF02837	1	Glycosyl hydrolase family 2
	PF00759	1	Glycosyl hydrolase family 9
	PF02055	1	Glycosyl hydrolase family 30
	PF01301	1	Glycosyl hydrolase family 35
	PF01074	1	Glycosyl hydrolase family 38, N-terminal domain
	PF04116	1	Fatty acid hydroxylase superfamily
	PF16884	1	N-terminal domain of oxidoreductase
Detoxification	PF00135	15	Carboxylesterase family
	PF00201	6	UDP-glucoronosyl and UDP-glucosyl transferase
	PF06757	4	Insect-allergen-repeat protein, nitrile-specifier detoxification
	PF00106	4	Short chain dehydrogenase
	PF00248	3	Aldo/keto reductase family
	PF02798	3	Glutathione S-transferase, N-terminal domain
	PF04488	2	Glycosyltransferase sugar-binding region
	PF01762	2	Galactosyltransferase
Nutrient absorption	PF00083	7	Sugar (and other) transporter
	PF01490	4	Transmembrane amino acid transporter protein
	PF00061	3	Cytosolic fatty-acid binding protein family
	PF16414	2	Niemann-Pick C1 N terminus
	PF00854	1	Proton-dependent oligopeptide transporter family
	PF13906	1	C-terminus of amino acid permease
Membrane pumps	PF07690	3	Major Facilitator Superfamily
	PF00664	1	ABC transporter transmembrane region
	PF00005	1	ABC transporter
	PF01061	1	ABC-2 type transporter
	PF00999	1	Sodium/hydrogen exchanger family
	PF03619	1	Organic solute transporter Ostalpha
	PF01496	1	V-type ATPase a subunit family
Other	PF00011	4	Heat shock protein 20 (Hsp20)
	PF06585	3	Haemolymph juvenile hormone binding protein (JHBP)
	PF07714	2	Protein tyrosine kinase
	PF01370	2	NAD dependent epimerase/dehydratase family
	PF13561	2	Enoyl-(Acyl carrier protein) reductase
	PF01683	2	EB domain—Function unknown
	PF00226	2	Chaperone DnaJ domain
	PF02518	2	Histidine kinase-, and HSP90-like ATPase
	PF16984	1	Group 7 allergen
	PF00012	1	Heat shock protein 70 (Hsp70)
	PF00183	1	Heat shock protein 83 (Hsp83)
	PF13855	1	Leucine rich repeat
	PF00063	1	Myosin head (motor domain)
	PF00076	1	RNA recognition motif
	PF00642	1	Zinc finger C-x8-C-x5-C-x3-H type/RNA-binding
	PF00096	1	Zinc finger, C2H2 type
	PF00390	1	Malic enzyme
	PF16177	1	Acetyl-coenzyme A synthetase N-terminus
	PF00109	1	Beta-ketoacyl synthase
	PF17297	1	Phosphoenolpyruvate carboxykinase, N-terminal domain
	PF03953	1	Tubulin C-terminal domain
	PF01553	1	Acyltransferase
	PF00085	1	Thioredoxin
	PF08211	1	Cytidine and deoxycytidylate deaminase
	PF00330	1	Aconitase family (aconitate hydratase)
	PF01869	1	BadF/BadG/BcrA/BcrD ATPase family
	PF00352	1	Transcription factor TFIID (or TATA-binding protein, TBP)
	PF02460	1	Patched family/membrane receptor for Sonic Hedgehog
	PF13637	1	Ankyrin repeats (many copies)
	PF01569	1	Phosphatidic acid phosphatase superfamily
	PF04193	1	PQ loop repeat
	PF13306	1	BspA type Leucine rich repeat region (6copies) domain
	PF01564	1	Spermine/spermidine synthase domain
	PF01510	1	N-acetylmuramoyl-L-alanine amidase
	PF07084	1	Thyroid hormone-inducible hepatic protein Spot 14
	PF06463	1	Molybdenum Cofactor Synthesis C
	PF06293	1	Lipopolysaccharide kinase (Kdo/WaaP) family
	PF00560	1	Leucine Rich Repeat
	PF00501	1	AMP-binding enzyme
	PF04055	1	Radical SAM superfamily
	PF05368	1	NmrA-like family
	PF04922	1	DIE2/ALG10 family
	PF02668	1	Taurine catabolism dioxygenase TauD, TfdA family
	PF03009	1	Glycerophosphoryl diester phosphodiesterase family
	PF17284	1	Spermidine synthase tetramerisation domain
	PF13606	1	Ankyrin repeat
	PF00389	1	D-isomer specific 2-hydroxyacid dehydrogenase
	PF07991	1	Acetohydroxy acid isomeroreductase
	PF13353	1	4Fe-4S single cluster domain
	PF12796	1	Ankyrin repeats (3 copies)
	PF12349	1	Sterol-sensing domain of SREBP cleavage-activation
	PF09335	1	SNARE associated Golgi protein
	PF08659	1	KR domain part of polyketide synthases
	PF13664	1	Domain of unknown function (DUF4149)
	PF16012	1	Domain of unknown function (DUF4780)
	PF06155	1	Protein of unknown function (DUF971)
	PF05705	1	Eukaryotic protein of unknown function (DUF829)

**TABLE 3:** Summary of Pfam families of downregulated transcripts 2 h after feeding versus 24 h after feeding.

Classification	Pfam	# DEGs	Description
Peritrophins	PF01607	5	Chitin binding Peritrophin-A domain
Enzymes	PF00089	2	Trypsin
	PF00351	1	Biopterin-dependent aromatic amino acid hydroxylase
	PF01019	1	Gamma-glutamyltranspeptidase
	PF00930	1	Dipeptidyl peptidase IV (DPP IV) N-terminal region
	PF01619	1	Proline dehydrogenase
Inhibitors	PF00079	1	Serpin (serine protease inhibitor)
Detoxification	PF00067	6	Cytochrome P450
	PF00106	1	Short chain dehydrogenase
	PF00135	1	Carboxylesterase family
	PF00171	1	Aldehyde dehydrogenase family
	PF16884	1	N-terminal domain of oxidoreductase
Membrane associated	PF00664	3	ABC transporter transmembrane region
	PF00083	2	Sugar (and other) transporter
	PF03522	1	Solute carrier family 12
	PF00860	1	Permease family
	PF00909	1	Ammonium Transporter Family
	PF00916	1	Sulfate permease family
	PF01061	1	ABC-2 type transporter
	PF00155	1	Aminotransferase class I and II
	PF04547	1	Calcium-activated chloride channel
	PF03600	1	Citrate transporter
	PF00061	1	Fatty acid binding protein
	PF00335	1	Tetraspanin family
Other	PF00595	2	PDZ domain
	PF01347	2	Lipoprotein amino terminal region
	PF03392	2	Insect pheromone-binding family
	PF03722	2	Hemocyanin, all-alpha domain
	PF12796	2	Ankyrin repeats (3 copies)
	PF06585	1	Haemolymph juvenile hormone binding protein (JHBP)
	PF00059	1	Lectin C-type domain
	PF00046	1	Homeodomain
	PF00078	1	Reverse transcriptase (RNA-dependent DNA polymerase)
	PF00096	1	Zinc finger, C2H2 type
	PF00041	1	Fibronectin type III domain
	PF00169	1	PH domain
	PF00560	1	Leucine Rich Repeat
	PF00875	1	DNA photolyase
	PF02014	1	Reeler domain
	PF02931	1	Neurotransmitter-gated ion-channel ligand binding domain
	PF04321	1	RmlD substrate binding domain
	PF04500	1	FLYWCH zinc finger domain
	PF05978	1	Ion channel regulatory protein UNC-93
	PF07177	1	Neuralized
	PF09298	1	Fumarylacetoacetase N-terminal
	PF15993	1	Fuseless
	PF12017	1	Transposase protein
	PF12803	1	mRNA (guanine-7-)methyltransferase (G-7-MTase)
	PF13516	1	Leucine Rich repeat
	PF16984	1	Group 7 allergen
	PF00650	1	CRAL/TRIO domain
	PF03729	1	Short repeat of unknown function (DUF308)

### 3.4 RNAi-induced gene silencing experiments

Two genes were selected for functional analysis because of their potentially important role in gut physiology and their upregulation 2 h after feeding compared to 24 h (Supplementary Figures S5A, B, one-way ANOVA with Holm-Šídák’s *post hoc* test, *SgVAHa1* RNA-seq: *p* = 0.00016, *SgNPC1b* RNA-seq: *p* = 0.00030). The first transcript ‘TR39983|c0_g2’ encodes a H^+^ V-ATPase 116 kDa subunit and is referred to as H^+^ V-ATPase subunit-a isoform 1 (*SgVAHa1*). The second transcript ‘TR102686|c0_g1’ encodes a Niemann-pick C1 b (NPC1b) protein and is referred to as *SgNPC1b*. In addition to *SgVAHa1*, two other transcripts annotated as H^+^ V-ATPase subunit-a were present in the *S. gregaria* midgut transcriptome, namely, ‘TR70116|c0_g1_i2’ and ‘TR91547|c0_g1_i1’ showing overall high sequence similarity to *SgVAHa1*. However, *SgVAHa1* was the only *SgVAHa* transcript that was significantly upregulated in the midgut 2 h after feeding compared to 24 h. Using *SgVAHa1* and *SgNPC1b* sequences as a query, nucleotide sequences encoding full open reading frames were retrieved from the *S. gregaria* genome (GenBank assembly accession GCA_023897955.2) for both genes (Supplementary Figures S3, S4). For *SgVAHa1*, the obtained amino acid sequence was identical to a previously annotated *S. gregaria* H^+^ V-ATPase 116 kDa subunit (GenBank accession number XP_049832128.1). A multiple sequence alignment, as well as a phylogenetic analysis, was performed for *SgVAHa1* (Supplementary Figures S6, S7) and *SgNPC1b* (Supplementary Figures S8, S9). The amino acid sequence alignments of both genes showed a high sequence similarity to their respective orthologs in other insect species (*SgVAHa1*: coverage = 97.2–99.6%, identity = 51.2–73.3%; *SgNPC1b*: coverage = 93.2–95.4%, identity = 30–39.3%). In addition, all of the 14 amino acid residues previously identified to be crucial for H^+^ V-ATPase subunit assembly and/or function were conserved in the *SgVAHa1* sequence (Leng et al., 1998) and *SgNPC1b* contained the three key motifs that are typical for NPC1 proteins: a patched domain (Pfam annotation PF02460), N-terminal domain (PF16414) and sterol-sensing domain (SSD, PF12349) (Zheng et al., 2018). Additionally, phylogenetic analyses of these genes are generally consistent with insect phylogeny and all species belonging to the same order were clustered together.

The RNA-seq results for *SgVAHa1* and *SgNPC1b* were validated by measuring mRNA levels in the midgut in all three conditions using RT-qPCR, confirming the induced expression of both genes in the midgut samples 2 h after feeding (Supplementary Figures S5C, D, one-way ANOVA with Holm-Šídák’s *post hoc* test, *SgVAHa1*: *p* = 0.0008, *SgNPC1b*: *p* = 0.0027). Furthermore, *SgVAHa1* transcript levels were already significantly elevated as soon as 10 min after feeding compared to 24 h (one-way ANOVA with Holm-Šídák’s *post hoc* test, *p* = 0.0057). Finally, the FCs 2 h after feeding compared to 24 h after feeding observed in this RT-qPCR analysis did not differ drastically from those observed in the RNA-Seq analysis (*SgVAHA1*: 1.067 vs. 1.225, *SgNPC1b*: 1.624 vs. 1.713 respectively).

#### 3.4.1 RNAi of *SgVAHa1*

**FIGURE 4:**
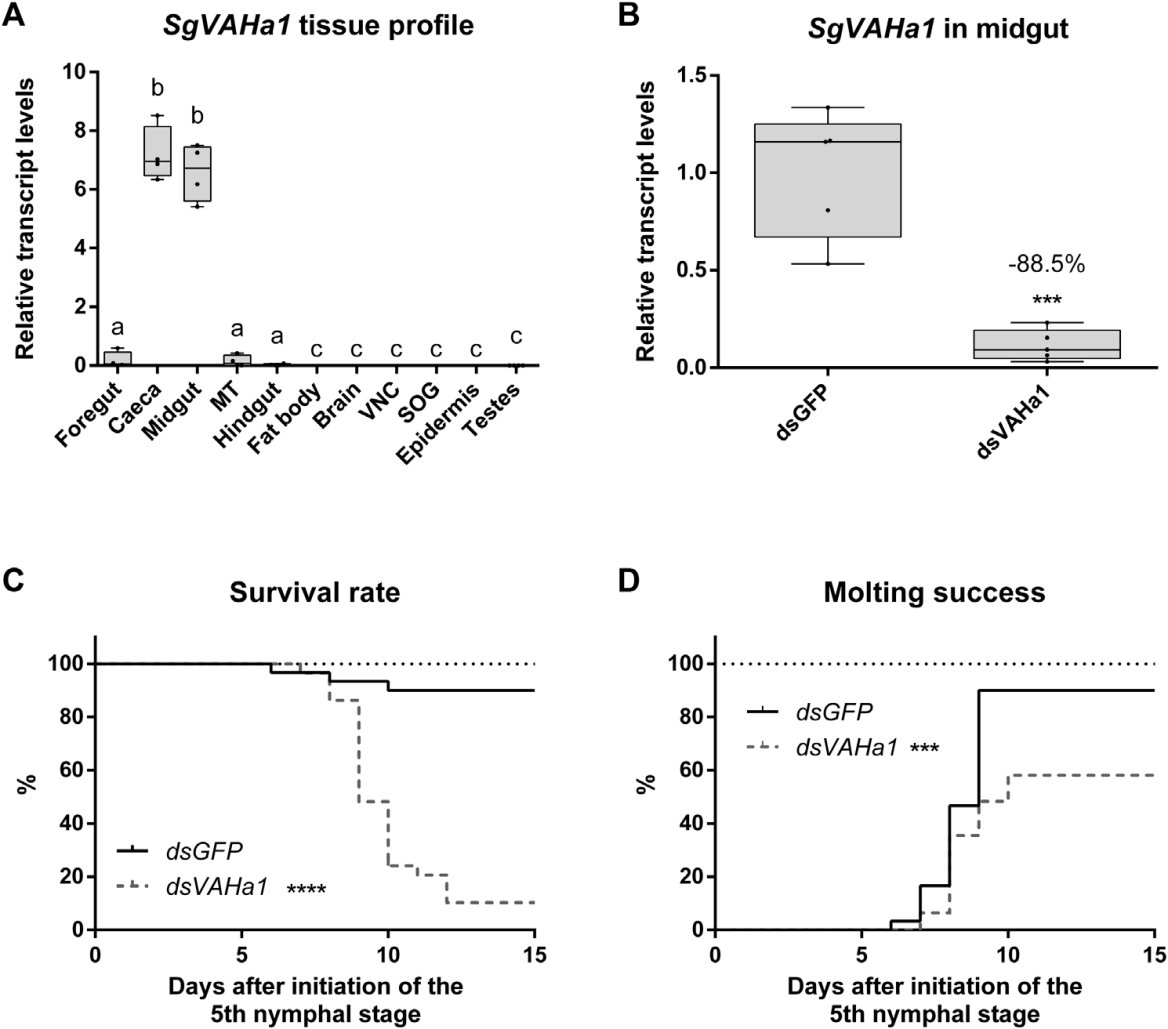
RNAi-induced knockdown of *SgVAHa1*. Animals injected with a dsRNA construct targeting *SgVAHa1* (*dsVAHa1*) were compared to control animals injected with *dsGFP*. *SgVAHa1* transcript levels **(A,B)** are represented by boxplots containing the upper and lower quartile, while the whiskers indicate the minimum and maximum. The median is indicated by the gray line within each box. Relative transcript levels were normalized using *SgGAPDH* and *SgRP49* as reference genes. Statistical significance (*p* ≤ 0.05) is either indicated by (a combination of) distinct characters [**(A)**, a, b, and c) or an asterisk (panel B–D, ***: *p* ≤ 0.001, ****: *p* ≤ 0.0001]. **(A)** Relative transcript levels of *SgVAHa1* were measured in a range of tissues (MT: Malpighian tubules, VNC: ventral nerve cord, SOG: suboesophageal ganglion) using RT-qPCR (*n* = 4) (one-way ANOVA with Holm-Šídák’s *post hoc* test; midgut and caeca compared to all other tissues: *p* < 0.0001; hindgut, foregut and MT compared to all other tissues: *p* < 0.005). **(B)** Relative mRNA levels of *SgVAHa1* in the midgut of *dsGFP* and *dsVAHa1* injected animals measured by RT-qPCR (*dsGFP*: *n* = 5, *dsVAHa1*, *n* = 5). Transcript levels of *SgVAHa1* were significantly reduced by 88.5% upon *dsVAHa1* injection (unpaired Student’s t-test, *p* = 0.0003). **(C)** Survival rate is expressed as the percentage of live animals. Animals injected with *dsVAHa1* (dashed gray line) had a significantly lower survival rate compared to control animals (full black line) (*dsGFP*: *n* = 30, *dsVAHa1*, *n* = 31, logrank Mantel-Cox test, *p* < 0.0001). **(D)** Molting success is expressed as the percentage of animals that successfully molted to the adult stage. Animals injected with *dsVAHa1* (dashed gray line) had significantly lower molting success compared to control animals (full black line) (*dsGFP*: *n* = 30, *dsVAHa1*, *n* = 31, logrank Mantel-Cox test, *p* = 0.0003).

*SgVAHa1* is predominantly expressed in the gastric caeca and in the midgut and only low transcript levels were detected in other parts of the digestive tract, namely, foregut, Malpighian tubules, and hindgut (Figure 4A). Transcript levels of *SgVAHa1* were significantly higher in caeca and midgut compared to all other tissues (*n* = 4; one-way ANOVA with Holm-Šídák’s *post hoc* test; midgut and caeca compared to all other tissues: *p* < 0.0001; hindgut, foregut and Malpighian tubules compared to all other tissues: *p* < 0.005). Upon injection of *dsVAHa1*, the midgut transcript levels of *SgVAHa1* on N5D6 were significantly reduced by 88.5% compared to animals injected with *dsGFP* (Figure 4B; *dsGFP: n* = 5, *dsVAHa1*: *n* = 5; unpaired Student’s t-test, *p* = 0.0003).

An RNAi-induced knockdown of *SgVAHa1* (*dsGFP*: *n* = 30, *dsVAHa1*: *n* = 31) significantly affected survival throughout the N5 stage (Figure 4C, logrank Mantel-Cox test, *p* < 0.0001), as well as molting from the nymphal to the adult stage (Figure 4D, logrank Mantel-Cox test, *p* = 0.0003). The first animals died on N5D6 (*dsGFP*) and N5D7 (*dsVAHa1*). The highest mortality occurred in the *dsVAHa1* condition on N5D9 (37.93%) and N5D10 (24.14%). On N5D10, only 24.14% of animals were still alive (*dsVAHa1*) compared to 90% in the control condition (*dsGFP*). The first animals molted to the adult stage at N5D6 (*dsGFP*) and N5D7 (*dsVAHa1*). On N5D9, only 28.85% of animals had molted (*dsVAHa1*) compared to all of the surviving *dsGFP* injected animals (90%). At the end of the experiment, a total of eighteen out of thirty-one animals (58.06%) from *dsVAHa1* injected animals had reached the adult stage. However, these animals were visibly unhealthy (our observations) and fifteen of these eighteen adults died within the duration of this experiment (15 days after the molt to the N5 stage). By contrast, all of the twenty-seven *dsGFP* injected animals that successfully molted to the adult stage remained healthy throughout this experiment.

#### 3.4.2 RNAi of *SgNPC1b*

**FIGURE 5:**
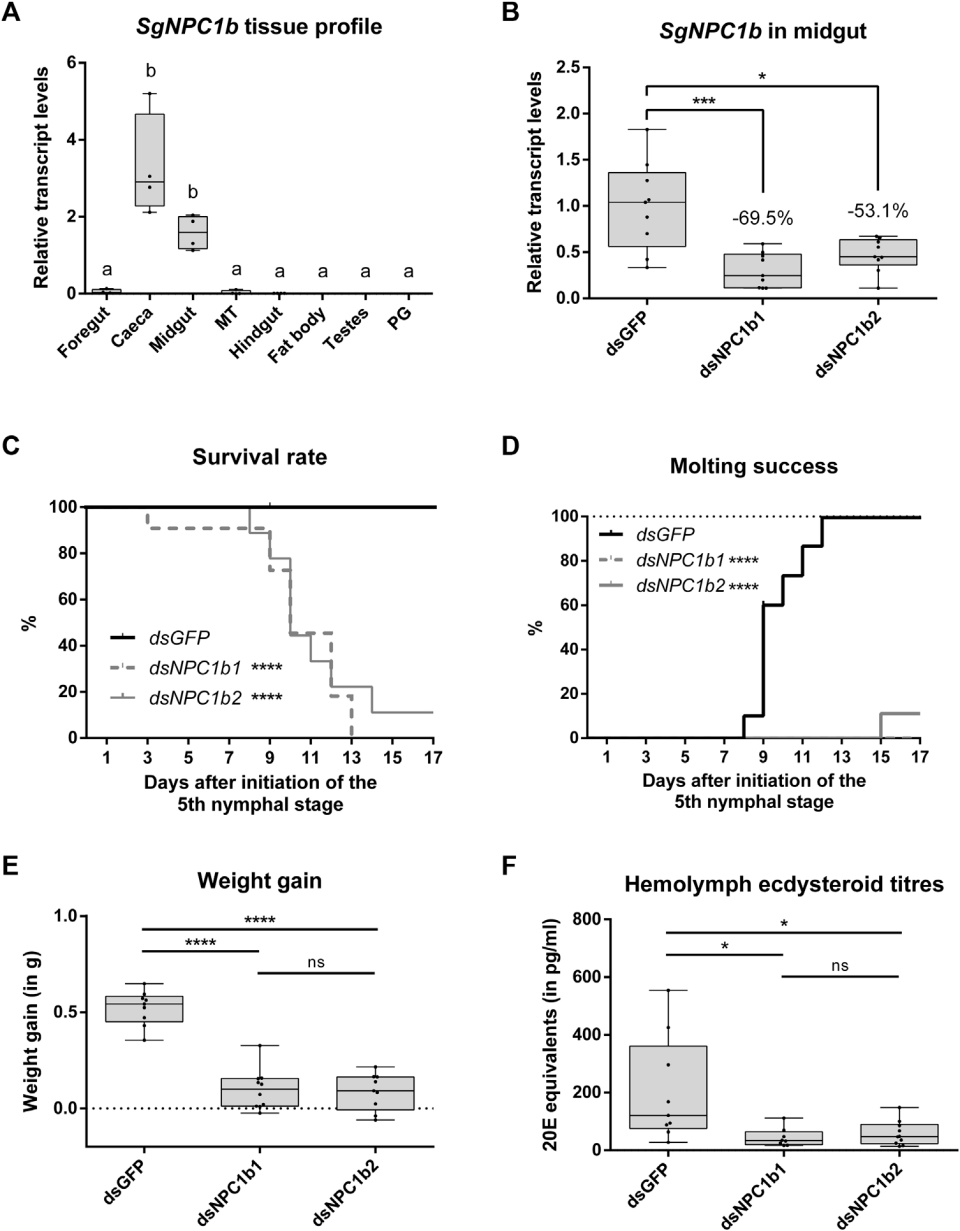
RNAi-induced knockdown of *SgNPC1b*. Animals injected with dsRNA targeting *SgNPC1b* (*dsNPC1b1* or *dsNPC1b2*) were compared to control animals injected with *dsGFP* (*dsGFP*: *n* = 10, *dsNPC1b1*: *n* = 11, *dsNPC1b2*: *n* = 9). Transcript levels of *SgNPC1b* **(A,B)**, weight gain **(E)** and hemolymph ecdysteroid titers **(F)** are represented by boxplots containing the upper and lower quartile, while the whiskers indicate the minimum and maximum. The median is indicated by the gray line within each box. Statistical significance (*p* ≤ 0.05) is either indicated by (a combination of) distinct characters [**(A)**, a and b] or an asterisk [**(B–E)**, *: *p* ≤ 0.05, ***: *p* ≤ 0.001, ****: *p* ≤ 0.0001]. Animals that died or escaped during the experiment were censored from the analysis and are indicated with ticks on the line graph **(C,D)**. **(A)** Relative transcript levels of *SgNPC1b* were measured in a range of tissues (MT: Malpighian tubules, PG: prothoracic gland) by RT-qPCR (*n* = 4). Relative transcript levels were normalized using *SgGAPDH* and *SgRP49* as reference genes. Relative transcript levels of *SgNPC1b* were significantly higher in caeca and midgut compared to other tissues (one-way ANOVA with Holm-Šídák’s *post hoc* test, midgut vs. foregut: *p* = 0.0003, all other pairwise comparisons: *p* < 0.0001). **(B)** Relative transcript levels of *SgNPC1b* in the midgut of *dsGFP* and *dsNPC1b* injected animals measured by RT-qPCR (*dsGFP*: *n* = 9, *dsNPC1b1*: *n* = 9, *dsNPC1b2*: *n* = 9). Transcript levels of *SgNPC1b* were significantly reduced by 69.5% and 53.1% upon *dsNPC1b1* and *dsNPC1b2* injection, respectively (one-way ANOVA with Holm-Šídák’s *post hoc* test, *dsNPC1b1*: *p* = 0.0004, *dsNPC1b2*: *p* = 0.0303). **(C)** Survival rate is expressed as the percentage of live animals. Animals injected with *dsNPC1b1* (dashed gray line) or *dsNPC1b2* (full gray line) had a significantly lower survival rate compared to control animals (full black line) (Logrank Mantel-Cox test, *dsNPC1b1*: *p* < 0.0001, *dsNPC1b2*: *p* < 0.0001). **(D)** Molting success is expressed as the percentage of animals that successfully molted to the adult stage. Animals injected with *dsNPC1b1* (dashed gray line) or *dsNPC1b2* (full gray line) had significantly lower molting success compared to control animals (full black line) (logrank Mantel-Cox test, *dsNPC1b1*: *p* < 0.0001, *dsNPC1b2*: *p* < 0.0001). **(E)** Weight gain (in g) is calculated as the difference in weight between day 0 of the N5 stage (N5D0) and N5D6. Weight gain was significantly lower upon injection of *dsNPC1b* (one-way ANOVA with Holm-Šídák’s *post hoc* test, *dsNPC1b1*: *p* < 0.0001, *dsNPC1b2*: *p* < 0.0001). **(F)** hemolymph ecdysteroid titers (expressed as 20E equivalents in pg/mL) as determined by EIA. Ecdysteroid titers were significantly lower in *dsNPC1b* injected animals compared to control (*dsGFP*) animals (one-way ANOVA with Holm-Šídák’s *post hoc* test, *dsNPC1b1*: *p* = 0.0164, *dsNPC1b2*: *p* = 0.0212).

The majority of *SgNPC1b* expression was measured in the gastric caeca and the midgut. By contrast, *SgNPC1b* transcript levels were low in other parts of the digestive tract, namely, foregut, Malpighian tubules, and hindgut (Figure 5A). Transcript levels of *SgNPC1b* were significantly higher in caeca and midgut compared to all other tissues (*n* = 4, one-way ANOVA with Holm-Šídák’s *post hoc* test, midgut vs. foregut: *p* = 0.0002, all other comparisons: *p* < 0.0001). Transcript levels of *SgNPC1b* were measured in the midgut on N5D6, 24 h after a single injection with one of the (non-overlapping) dsRNAs targeting *SgNPC1b* (*dsNPC1b1 or dsNPC1b2*) in 24 h starved animals, and were significantly reduced by 69.5% (*dsNPC1b1*) and 53.1% (*dsNPC1b2*) compared to *dsGFP* injected animals (Figure 5B; *dsGFP*: *n* = 9, *dsNPC1b1*: *n* = 9, *dsNPC1b2*: *n* = 9; one-way ANOVA with Holm-Šídák’s *post hoc* test, *dsNPC1b1*: *p* = 0.0004, *dsNPC1b2*: *p* = 0.0303).

An RNAi-induced knockdown of *SgNPC1b* (*dsGFP*: *n* = 10, *dsNPC1b1*: *n* = 11, *dsNPC1b2*: *n* = 9) significantly affected survival throughout the N5 stage (Figure 5C, logrank Mantel-Cox test, *dsNPC1b1*: *p* < 0.0001, *dsNPC1b2*: *p* = 0.0003), as well as molting from the nymphal to the adult stage (Figure 5D, logrank Mantel-Cox test, *dsNPC1b1*: *p* < 0.0001, *dsNPC1b2*: *p* = 0.0001). The first animals died at N5D3 (*dsNPC1b1*) and N5D8 (*dsNPC1b2*). All but one *dsNPC1b* injected animal died during the N5 stage. The only surviving knockdown animal, injected with *dsNPC1b2*, molted to the adult stage at N5D15. By contrast, no control animals (*dsGFP*) died during the experiment. All control animals molted to the adult stage between N5D8 and N5D12. The total body weight was monitored from N5D0 onwards (Figure 5E). Weight gain was determined as the difference in total body weight between N5D0 and N5D6. Control animals gained significantly more weight, approximately doubling in weight, compared to both knockdown conditions (one-way ANOVA with Holm-Šídák’s *post hoc* test, *dsNPC1b1*: *p* < 0.0001, *dsNPC1b2*: *p* < 0.0001). Finally, ecdysteroid levels were measured in the hemolymph at N5D6 and expressed as 20E equivalents (Figure 5F, *dsGFP*: *n* = 9, *dsNPC1b1*: *n* = 8, *dsNPC1b2*: *n* = 10). Upon knockdown of *SgNPC1b*, ecdysteroids levels in the hemolymph were significantly reduced (one-way ANOVA with Holm-Šídák’s *post hoc* test, *dsNPC1b1: p* = 0.0184, *dsNPC1b2*: *p* = 0.0184). No significant differences in survival rate, molting success, weight gain or hemolymph ecdysteroid levels were observed between the treatments with non-overlapping *dsNPC1b* constructs.

## 4 Discussion

### 4.1 The *S. gregaria* midgut transcriptome

In this study, we sequenced transcripts expressed in the midgut at three post-feeding timepoints. At least one functional annotation was retrieved for 56.5% of the total 19,345 transcripts. Consequently, 43.5% of transcripts did not retrieve any functional annotation, which is reflected by “Function unknown” being the largest represented COG group (20.5%, Figure 2). However, the functions attributed to (most of) these transcripts are predicted by bioinformatic analysis and are not conclusively proven. Annotation of the transcripts was primarily based on BLASTs, searching for regions of similarity between sequences of various species. Sequence identification was therefore strongly based on the assumed conservation of functions. Many transcripts in the *S. gregaria* midgut are possibly too divergent from sequences in other species. Alternatively, a subset of these transcripts possibly consists of either orthologues of conserved insect genes, or *Schistocerca-*specific genes, whose functions have yet to be discovered. Indeed, the abundance of unannotated transcripts is in line with research on other insects (Pauchet et al., 2009; Ma et al., 2012; Gazara et al., 2017; Huang et al., 2017; Dhania et al., 2019; Coutinho-Abreu et al., 2020). Both GO (Figure 1) and COG (Figure 2) functional classifications were consistent with midgut transcriptomic studies in other insect species (Pauchet et al., 2009; Spit et al., 2016b; Huang et al., 2017). Therefore, the limitations of bioinformatic predictions and functional annotations based on large sequencing datasets clearly demonstrate the need for more functional research on these transcripts. Functional characterization and validation of these transcripts will require time and will depend on the scientific progress in insect (*i.c.* locust) molecular physiology. Nevertheless, we provided a high-quality comprehensive database of transcript sequences obtained from the midgut of the desert locust, highlighting potential areas of interest for future investigations.

### 4.2 Differential expression analysis

We clearly illustrated the contrasting gene expression in the *S. gregaria* midgut at 2 h *versus* 24 h after feeding (Figure 3). A total of 569 and 212 transcripts were significantly up- and downregulated, respectively, in the 2 h condition compared to the 24 h condition. By contrast, no DEGs were detected for the other pairwise comparisons. At 24 h after feeding, only some highly digested remnants remained present inside the midgut lumen, while the foregut was empty. On the other hand, at 2 h after feeding, ingested plant material was observed inside the midgut. At 10 min after feeding, food was present in the foregut, while the midgut content was similar in appearance to that of the 24 h condition. We hypothesize that the 24 h condition was possibly the least subjected to variation because the digestive activity is drastically reduced in the absence of food. On the other hand, the presence of food in the alimentary tract (10 min and 2 h conditions) induced various biological processes. It is possible that the sensing of food and/or food entering the foregut may already have resulted in a rapid transcriptional response in the midgut. Therefore, the 10 min condition is likely a transitional timepoint between the 24 h and the 2 h conditions. This is supported by the high variation in transcript profiles between samples at 10 min after feeding, as the samples of the 10 min condition do not cluster based on PC1 and PC2 (Supplementary Figure S2). These findings suggest that the *S. gregaria* midgut can respond very swiftly at the transcriptional level to the uptake of food.

In accordance with the reference transcriptome, we also observed a high number of unannotated DEGs. Of the annotated transcripts, those putatively involved in enzymatic digestion, peritrophic membrane formation, nutrient digestion and xenobiotic metabolism were strongly upregulated in response to food uptake. This indicates that the organism fully invests in digestion and its associated processes. On the other hand, none of the downregulated transcripts were predicted, based on their Pfam annotation to exhibit extracellular digestive or nutrient absorption activity. Nevertheless, it cannot be excluded that the proteins encoded by these downregulated transcripts play important roles during periods of food deprivation, such as mediating stress tolerance, tissue maintenance/protection, or immune defenses. The following paragraphs will discuss the most important findings related to midgut-associated functions.

#### 4.2.1 Digestive enzymes and nutrient transporters

We identified various transcripts predicted to code for putative proteases, carbohydrases, lipases, and nucleases (Supplementary Table S5). Such broad repertoires of digestive enzymes are frequently observed in insects (Campbell et al., 2005; Pauchet et al., 2009; Ma et al., 2012; Spit et al., 2016a; Zhang et al., 2016). Nevertheless, important species-specific differences in digestive enzyme repertoires are observed amongst insects, partially due to different dietary preferences and midgut luminal pH (Holtof et al., 2019). The fact that the majority of putative proteolytic enzyme encoding transcripts identified here code for serine proteases is in line with previous research (Campbell et al., 2005; Spit et al., 2012; Bonelli et al., 2020; Coutinho-Abreu et al., 2020). The proteolytic activity in the midgut of *S. gregaria* has indeed been mainly attributed to serine proteases, such as trypsins and chymotrypsins, but was also revealed to contain minor cysteine and carboxypeptidase activity (Spit et al., 2012). This high serine protease activity has also been found in other orthopterans (Spit et al., 2014; Huang et al., 2017). However, this is not the case for the more acidic midgut of Coleoptera, and Hemiptera, where the most abundant proteolytic activity is due to cysteine peptidases (Pauchet et al., 2009; Valencia et al., 2016; Oppert et al., 2018; Holtof et al., 2019; Pimentel et al., 2020).

Interestingly, three putative cellulases were identified in the locust midgut transcriptome. This is in accordance with the more recent findings that an endogenous cellulase activity is present in some insect species, such as many cockroaches and termites (Blattaria), the cricket *Teleogryllus emma*, the beetle *Tribolium castaneum* and the fly *Musca domestica*, amongst others (Watanabe and Tokuda, 2010; Holtof et al., 2019). However, genome encoded cellulase enzymes appear to be absent in *Anopheles gambiae*, *Drosophila melanogaster* and *B. mori* (Watanabe and Tokuda, 2010). In conclusion, our data align with the general observation that insects deploy divergent sets of enzymes to mediate the enzymatic digestion of food.

In addition to putative digestive enzymes, we observed the presence and upregulation upon feeding of putative transporters for all of the most abundant nutrient classes, namely, amino acids, carbohydrates, and lipids (Table 2; Supplementary Table S6). We identified several transcripts encoding proteins putatively involved in dietary lipid and sterol uptake. Fatty acid transport proteins, as well as scavenger receptors class B type I, have been suggested to interact in the active transport of dietary fatty acids across the midgut membrane, which is supported by their high abundance in the midgut transcriptome (Holtof et al., 2019). Additionally, various putative sterol transporters were identified, namely, sterol carrier protein x (SCPx), SCP2, and NPC1 proteins.

#### 4.2.2 Xenobiotic metabolism and detoxification processes

Insects often encounter various toxic or xenobiotic compounds that can be harmful if they are not properly dealt with. Herbivorous polyphagous species, like *S. gregaria*, have to typically rely on a wide range of detoxification mechanisms. This is especially true for insects feeding on complex diets with a high diversity of allelochemicals, such as green leaves, in contrast to diets less diverse in allelochemicals, such as plant sap, nectar and pollen (Rane et al., 2019). Our transcriptome data indeed predicted the presence of various counter-defensive mechanisms in the *S. gregaria* midgut. This is based on the abundant presence of transcripts predicted to encode NPSs, UGTs, GSTs, CEs, P450s as well as ABC transporters (Supplementary Table S7), all of which have been shown previously to play important roles in xenobiotic metabolism and detoxification in a wide range of species (Rane et al., 2019; Denecke et al., 2021). Furthermore, multiple transcripts for each of these protein classes, with the exception of P450, were upregulated upon feeding (Table 2). Such differential gene expression patterns upon exposure to xenobiotics have been observed in several other species of insects (Herde and Howe, 2014; Spit et al., 2016b; Dhania et al., 2019; Shu et al., 2021; Jin et al., 2022). Our findings suggest that *S. gregaria* actively detoxifies its ingested food in the midgut by swiftly increasing the transcript levels of several detoxification systems. Additionally, we hypothesize that the broad repertoire of putative detoxification mechanisms encountered here is a consequence of the polyphagous herbivorous lifestyle of this locust species, implying that it can cope with a large diversity of plant toxins in its diet.

One of the plant toxins most often encountered by the locusts in our colony is glucosinolate from cabbage (Barba et al., 2016). We showed the upregulation of several putative NSPs, UGTs and GSTs, which have all been shown to be specifically involved in defense against glucosinolate-derived toxins (Burow et al., 2006; Li et al., 2018), suggesting that these proteins may also have a detoxifying function in the *S. gregaria* midgut. High levels of NSPs have been identified in the midguts of Lepidoptera as well as the locust *L. migratoria* (Fischer et al., 2008; Spit et al., 2016a). Interestingly NSPs isolated from other insect species, including *A. gambiae* and *Blattella germanica*, showed binding capacity to human immunoglobulin E, provoking an allergic reaction in atopic persons (Wang, Lee, and Wu, 1999). Locust NSPs might therefore provoke allergies in a similar manner. Six upregulated transcripts are predicted to belong to the multidrug resistance protein 1 (MRP1) family, including the ABC transporter subfamilies ABCB, ABCC and ABCG, which are described to have important functions in the xenobiotic metabolism of a wide range of insect species (Labbé et al., 2011; Sodani et al., 2012; Dermauw and Van Leeuwen, 2014; Qi et al., 2016; Denecke et al., 2021). Among these MRPs, one was further categorized as a P-glycoprotein 1 (ABCB1), which has previously been shown to be present in the brain barrier of *S. gregaria* (Andersson et al., 2014). P-glycoprotein 1, and other MRPs, such as MRP49 (ABCB1) and MRP1 (ABCC1), have been shown to play a role in pesticide resistance (Tian et al., 2013; Denecke et al., 2021). In summary, we showed the presence of various transcripts encoding proteins putatively involved in xenobiotic metabolism, as well as their upregulation upon feeding. Our findings therefore illustrate that polyphagous herbivorous insects are able to rely on various defense mechanisms against a large array of toxins. Interestingly, insects have been shown to rely on similar mechanisms for their protection against natural plant toxins and synthetic pesticides (Hawkins et al., 2019). How they succeed in doing so is still an important subject of study. Identifying and characterizing the underlying patterns orchestrating metabolic resistance against pesticides will be crucial in combatting insect pesticide resistance going forward.

In summary, based on our differential gene expression analysis in *S. gregaria* as well as those described in literature, it is apparent that gene expression in the insect midgut can respond rapidly to environmental changes, such as the presence of food, infection or exposure to xenobiotics. Based on our data, as well as reports in literature, multiple transcripts encoding putative digestive enzymes, nutrient transporters and other digestion-related genes are abundantly upregulated upon feeding (Sanders et al., 2003; Xu et al., 2012; Cui and Franz, 2020). By contrast, immunity-related genes and detoxification enzymes are strongly upregulated upon infection or exposure to xenobiotics, respectively, while digestion-related genes are downregulated, although these reactions are often complex (Noland et al., 2013; Herde and Howe, 2014; Dhania et al., 2019; Coutinho-Abreu et al., 2020; Kuang et al., 2021; Shu et al., 2021; Jin et al., 2022).

### 4.3 RNAi of *SgVAHa1*

#### 4.3.1 Structure and function of H^+^ V-ATPases

Based on our multiple sequence alignment (Supplementary Figure S6) and phylogenetic analysis (Supplementary Figure S7), we can conclude that *SgVAHa1* is a true ortholog of insect H^+^ V-ATPase subunit-a. We observed a significant upregulation of a H^+^ V-ATPase subunit-a 2 h after feeding, indicating that this subunit is under transcriptional regulation. The H^+^ V-ATPases are highly conserved transmembrane proton pumps which in insects occur mostly in various epithelial tissues, such as the salivary glands, the gut, Malpighian tubules, and the nervous system (Toei et al., 2010). These protein complexes use the energy released from hydrolyzing ATP to translocate protons across the apical membrane towards the extracellular fluid or across lysosomal or endosomal membranes towards the lumen of these intracellular compartments. This proton transport generates an electrochemical gradient enabling numerous biological processes, such as regulation of the internal pH of intracellular compartments, lysosomal degradation, loading of cargo into secretory vesicles, and maintaining the polarity of midgut epithelial cells (Banerjee and Kane, 2020). Furthermore, it is well established that in insects this proton motive force drives cation/H^+^ exchangers (i.e., K^+^/H^+^ and Na^+^/H^+^ antiporters) and therefore the transepithelial secretion of ions, which in turn stimulates Na^+^ or K^+^ coupled nutrient uptake (e.g., Na^+^ coupled glucose transporters) (Torrie et al., 2004; Wieczorek et al., 2009; Halberg and Møbjerg, 2012). However, the interaction between the electrochemical gradient generated by H^+^ V-ATPase and the secondary transport of inorganic ions by Na^+^/K^+^-ATPase at the basal membrane of epithelial cells is still unclear (Halberg and Møbjerg, 2012). The H^+^ V-ATPase protein complex consists of various subunits, further subdivided into a peripheral V_1_ domain consisting of eight different subunits (A-H) and an integral, membrane-bound, V_0_ domain consisting of six different subunits (a-e) (Toei et al., 2010). The V_1_ region contains the catalytic site converting ATP into energy, which is used for proton transport, in which subunit-a plays a crucial role. In addition, subunit-a was shown to be important for coupling specific V_1_ and V_0_ domains to different cellular sites (Kawasaki-Nishi et al., 2001; Sun-Wada and Wada, 2022) through interaction with specific phospholipids (Banerjee and Kane, 2020). Furthermore, the N-terminal domain of subunit-a has been described as a regulatory hub, incorporating environmental input, such as the glycolytic enzyme aldolase, and thereby regulating both assembly/disassembly and localization of the H^+^ V-ATPase complex to specific cellular membranes (Banerjee and Kane, 2020).

#### 4.3.2 *SgVAHa1* transcript profile

The *SgVAHa1* tissue profile highlights large differences in relative transcript levels (Figure 4A) and suggests a specialized function of *SgVAHa1* in the midgut and associated caeca. In addition to *SgVAHa1*, two other unique H^+^ V-ATPase subunit-a isoform sequences were also identified in our study. However, their tissue-dependent expression profiles were not evaluated since they were not differentially enriched upon feeding. It is currently unclear whether these isoforms perform different (tissue-specific) functions compared to *SgVAHa1*. More research is therefore needed to functionally characterize the different *S. gregaria* H^+^ V-ATPase subunit-a isoforms.

Differential distribution is typical for H^+^ V-ATPase subunits isoforms, including subunit-a (Kawasaki-Nishi et al., 2001; Toei et al., 2010; Chintapalli et al., 2013; Sun-Wada and Wada, 2022). All four isoforms of subunit-a in mice show different localization in embryos (Wada and Wada, 2022). In yeast two subunit-a isoforms occur, Stv1p which is localized to the vacuole and Vph1p which is localized to the Golgi apparatus (Kawasaki-Nishi et al., 2001). In insects, such tissue-specific expression of H^+^ V-ATPase subunit-a isoforms has been analyzed in *D. Melanogaster, T. castaneum,* and *L. migratoria* (Chintapalli et al., 2013; Mo et al., 2020; Liu et al., 2022; Naseem et al., 2023). In *D. melanogaster*, a midgut-specific H^+^ V-ATPase subunit-a isoform, called *vha100-4*, was identified. Other *D. melanogaster* H^+^ V-ATPase subunit-a isoforms are more uniformly expressed across tissues, with the exception of *vha100-3* which is almost exclusively expressed in the testes (Chintapalli et al., 2013). Moreover, differential expression of subunit-a isoforms within the midgut has been observed in *D. melanogaster* (Overend et al., 2016). The midgut-specific *vha100-4* is expressed exclusively in the acidic region of the midgut, while other isoforms are expressed over the entire length of the midgut (Overend et al., 2016). Multiple *D. melanogaster* subunit-a isoforms were shown to perform different functions in wing development within the same tissue (Mo et al., 2020). Similarly to what is observed in *D. melanogaster*, a H^+^ V-ATPase subunit-a isoform from *T. castaneum* shows differential expression across different epithelia with the highest expression being measured in the posterior midgut, while three out of five isoforms are not expressed in the midgut (Naseem et al., 2023). Two isoforms were identified in *L. migratoria*, showing distinct tissue-dependent and developmental expression profiles, although they were not highly expressed in the midgut or the gastric caeca (Liu et al., 2022). One isoform is most highly expressed in the hindgut and to a lesser extent in the fat body and trachea, while the second isoform is most highly expressed in the fat body. Tissue-specific expression patterns of different subunit isoforms was suggested to be most likely linked to local, tissue-specific V-ATPase functions (Sun-Wada and Wada, 2022). In many cases however, distinct H^+^ V-ATPase subunit isoforms have not been fully functionally characterized, especially in insect species. Furthermore, it is still unclear whether different subunit isoforms perform different functions within the same tissue, as so far this has only been described in the wing of *D. melanogaster* (Mo et al., 2020). Therefore, it would be of great interest to study the tissue-dependent expression profiles of three *SgVAHa* isoforms identified in the midgut in this study as well as possible other isoforms and their subsequent functional characterization. This would add an interesting perspective from a hemimetabolan insect species to the current knowledge available from the holometabolan model organisms, *D. melanogaster* and *T. castaneum* (Chintapalli et al., 2013; Naseem et al., 2023).

#### 4.3.3 *SgVAHa1* knockdown causes severe mortality and molting defects

RNAi-induced gene silencing of *SgVAHa1* resulted in high mortality rates and impaired molting (Figure 4), illustrating the crucial importance of *SgVAHa1* in the midgut. The majority of the experimental animals died shortly after molting to the adult stage, while some died during the molting process. However, based on our results, it is currently unclear what exactly is causing lethality. We propose two hypotheses, which may not be mutually exclusive. First, lethality might have resulted from a defective uptake of essential nutrients due to the silencing of the H^+^ V-ATPase complex, since its formation and the subsequently induced favorable membrane potential, may have been inhibited upon silencing *SgVAHa1*. Secondly, *SgVAHa1* is important for the integrity and function of the midgut epithelium, including the polarity of the epithelial cells and optimal functioning of various cell organelles. The first hypothesis is supported by the upregulation of *SgVAHa1* as soon as 10 min after feeding (Supplementary Figure S5C) in conjunction to the upregulation of various transporters for carbohydrates, amino acids, and lipids 2 h after feeding (Table 2). The swift upregulation of a H^+^ V-ATPase V_0_ domain subunit in the midgut in response to feeding has only been previously described in the mosquito *A. aegypti*, namely, the upregulation of subunits a and c (as well as various subunits of the V_1_ domain) 12 h after a bloodmeal (Sanders et al., 2003). In addition, to our knowledge only one other study in insects reported the upregulation of subunits of the V_1_ domain in response to feeding, namely, the upregulation of subunit C after a bloodmeal in the mosquito *Lutzomyia longipalpis* (Pitaluga et al., 2009). Furthermore, starvation in *Manduca sexta* caused disassembly of the H^+^ V-ATPase complex, possibly to reduce energy expenditure (Sumner et al., 1995). It would therefore be valuable to study the nutrient uptake at the midgut epithelium or nutrient levels in the hemolymph upon silencing *SgVAHa1*. In the locust, *L. migratoria,* a combined knockdown of two H^+^ V-ATPase subunit-a isoforms was obtained by injecting dsRNA directed against a sequence common to both isoforms. This treatment resulted in mortality and molting defects, similarly to what we observed here (Liu et al., 2022). Targeting these isoforms led to a decrease in food intake and body weight. Interestingly, researchers found no food in the midgut lumen of treated animals, fewer columnar epithelial cells and the absence of the brush border in some regions of the midgut epithelium (Liu et al., 2022). Similarly, knockdown of H^+^ V-ATPase subunit-a genes (*vha100*) or a subunit-B gene (*vha55*) in *D. melanogaster* led to increased pH in the acidic region of the midgut (Overend et al., 2016; Koyama et al., 2021), and *vha55* knockdown resulted in impaired nutrient absorption and mortality (Koyama et al., 2021). It is remarkable that subunit-a is the only H^+^ V-ATPase subunit that was significantly upregulated in the *S. gregaria* midgut 2 h post-feeding. It is possible that subunit-a is under strict transcriptional control because of its critical role in H^+^ V-ATPase assembly as well as in targeting specific V_1_ domains to specific membranes.

A number of studies have evaluated the insecticidal potential of targeting H^+^ V-ATPase subunits in several insect species, including a variety of insect pests, showing strong negative impacts on growth and survival (Ahmed, 2016; Sato et al., 2019; Rahmani and Bandani, 2021; Guo et al., 2022; Liu et al., 2022; Shi et al., 2022; Zeng et al., 2022). So far, the majority of these studies focused on targeting the different subunits of the V_1_ domain of these proton pumps. To the best of our knowledge, only two studies describing the targeting of subunit-a of the H^+^ V-ATPase V_0_ domain in insects exist (Ahmed, 2016; Liu et al., 2022). In both *L. migratoria* and the lepidopteran *Pectinophora gossypiella* a H^+^ V-ATPase subunit-a knockdown resulted in strong negative impacts on insect growth and survival, clearly illustrating the strong insecticidal potential of targeting insect H^+^ V-ATPases, including *SgVAHa1*.

### 4.4 RNAi of SgNPC1b

#### 4.4.1 Niemann-pick proteins

Both NPC1 and NPC2 proteins were identified in the *S. gregaria* midgut. Based on our multiple sequence alignment (Supplementary Figure S8) and phylogenetic analysis (Supplementary Figure S9), we conclude that *SgNPC1b* is a true ortholog of insect NPC1b. We observed a significant upregulation of *SgNPC1b* 2 h after feeding. Niemann-pick proteins are a family of transmembrane sterol-binding proteins known to play an important role in sterol absorption in the digestive tract of both mammals and insects (Davies et al., 2005; Voght et al., 2007; Zheng et al., 2018; Jing and Behmer, 2020). Even though the sterol uptake pathways in mammals and insects are similar, the exact role of Niemann-pick proteins in insects has so far only been studied in *D. melanogaster* and *Helicoverpa armigera*. Functional research in other insect species is currently lacking. NPC1 proteins typically contain three conserved domains which all show sterol-binding activity: N-terminal domain, patched domain and SSD (Voght et al., 2007; Zheng et al., 2018; Jing and Behmer, 2020). Due to gene duplication, the majority of insects possess two NPC1 homologues, namely, *NPC1a* and *NPC1b* (Zheng et al., 2018). NPC1b was shown to be crucial for dietary sterol absorption in the midgut of *D. melanogaster* and *H. armigera* (Voght et al., 2007; Zheng et al., 2020). After absorption, sterols are taken up in the lysosomes and are transferred by NPC2 to NPC1a which is integrated in the lysosomal membrane (Jing and Behmer, 2020). In insect herbivores, such as *S. gregaria*, plant sterols are dealkylated to form cholesterol, likely in the smooth endoplasmic reticulum of the midgut epithelium (Li and Jing, 2020).

#### 4.4.2 *SgNPC1b* transcript profile

*SgNPC1b* shows a midgut and associated caeca specific expression (Figure 5A), similar to *SgVAHa1*, implying a specialized function of SgNPC1b in the midgut, which is consistent with findings in the holometabolan species, *D. melanogaster*, *H. armigera, T. castaneum and Tenebrio molitor* (Voght et al., 2007; Oppert et al., 2018; Zheng et al., 2020). In line with these reports, we found two NPC1-like sequences in *T. castaneum* in the BeetleAtlas^9^ database (Naseem et al., 2023), one putative *NPC1a* sequence (TC005088) with uniform distribution and one putative *NPC1b* sequence (TC033918) with midgut-specific expression. Furthermore, *NPC1b* expression in the digestive system is probably not restricted to insects, since *NPC1L1*, the *NPC1b* ortholog in mammals, is highly expressed in the human liver and to a lesser extent the small intestine (Davies et al., 2005).

#### 4.4.3 *SgNPC1b* knockdown impairs growth and causes severe mortality and molting defects

Injection of *dsNPC1b* caused impaired weight gain, lowered hemolymph ecdysteroid titers, impaired molting, and high mortality (Figure 5). As NPC1b has been shown to be a midgut sterol transporter (Voght et al., 2007; Zheng et al., 2020), the phenotypes we observed here are expected to be the consequence of impaired sterol uptake. Arthropods do not synthesize sterols *de novo*, and thus rely entirely on intake of dietary sterols (Behmer, 2017; Li and Jing, 2020). Therefore, we propose three plausible, not mutually exclusive, hypotheses explaining the phenotypes we observed: 1. Sterols are crucial for normal growth and development since they are an integral component of biological membranes; 2. Sterols play a signaling role, being an important cue for nutritional status and thereby regulating growth and development; 3. Plant sterols are converted to cholesterol, which is the main precursor for ecdysteroid biosynthesis and therefore required for ecdysteroid hormone signaling.

Our data revealed several important phenotypes. We measured a significantly lower ecdysteroid titer in the hemolymph of *SgNPC1b* knockdown *versus* control locusts on N5D6, a developmental time point at which increased ecdysteroid levels are expected to occur in hemolymph (Verbakel et al., 2021). A possible explanation for this observation is that silencing *SgNPC1b* causes impaired sterol uptake in the midgut, resulting in a lack of cholesterol as precursor for ecdysteroid biosynthesis. However, this may only partially explain the observations. Locusts may still have sufficient access to cholesterol as a precursor for ecdysteroid biosynthesis based on the following arguments: 1. We knocked down *SgNPC1b*, meaning that we did not generate a complete knockout and presumably sterol uptake was not completely inhibited; 2. Locusts might still rely on stored steryl esters, which could potentially suffice to maintain ecdysteroid biosynthesis (Korber et al., 2017); 3. Most cholesterol is incorporated into cellular membranes and only a small fraction is needed for ecdysteroid biosynthesis (Jing and Behmer, 2020); 4. Alternative dietary sterol uptake mechanisms might still be in place, such as SCP (Guo et al., 2009). This suggests that it is unlikely that the *SgNPC1b* knockdown only affected ecdysteroid biosynthesis directly, due to the lack of cholesterol precursor. It is well described in insects that the attainment of a critical weight is an important trigger to stimulate ecdysteroid biosynthesis for metamorphosis or molting (Pan et al., 2021). Therefore, we suggest that the impaired weight gain during the N5 stage may also have inhibited or postponed ecdysteroid biosynthesis indirectly, resulting in the observed lower ecdysteroid titers in hemolymph. Anyway, it seems unlikely that the observed phenotypic effects, namely, reduced weight gain, impaired molting, and high mortality, were solely the consequence of a lower ecdysteroid titer. The effects on weight gain already occurred at the start of the N5 stage, long before the expected rise in ecdysteroid levels (around N5D6). Interestingly, in *D. melanogaster NPC1b* mutants, administration of exogenous ecdysone did not affect mortality, indicating that reduced ecdysteroid biosynthesis by itself does not explain all phenotypes observed upon impaired sterol uptake (Voght et al., 2007).

Instead, we suggest that the lethal phenotype we observed may be a consequence of impaired membrane integrity and functionality, especially at the midgut epithelium. Lack of sterols in the cellular membrane is known to cause mortality by compromising membrane integrity, affecting the function of membrane-bound enzymes, endocytosis, receptor binding and signal transduction (Behmer and Elias, 1999; Gasque et al., 2005). Nevertheless, in *D. melanogaster* sterols appeared to be dispensable for maintaining membrane properties such as impermeability during periods of extreme dietary sterol restriction. During such periods these membrane properties were supplied by non-sterol lipids, such as sphingolipids (Carvalho et al., 2010). In contrast to *D. melanogaster*, acridid grasshoppers (Orthoptera) do not tolerate high proportions of non-cholesterol sterols as structural components in their membranes, while the tolerance in the lepidopterans *Heliothis virescens* and *Helicoverpa zea* is somewhere in between (Behmer and Elias, 2000; Jing et al., 2014; Jing and Behmer, 2020). This suggests that acridid grasshoppers (Orthoptera), such as *S. gregaria*, are more vulnerable to reduced cholesterol levels as they need mostly cholesterol for maintaining membrane integrity.

Interestingly, cholesterol has been shown to perform an important signaling function in *D. melanogaster* (Texada et al., 2022). Cholesterol serves as a nutritional signal, mediated by the target of rapamycin pathway in the fat body, which stimulates growth and development by increasing insulin-like peptide transcription and release in the insulin-producing cells of the brain. Furthermore, the growth-promoting effect of cholesterol requires insulin signaling and is not the result of effects on ecdysteroid biosynthesis. It is currently unclear whether cholesterol has a growth-promoting effect in *S. gregaria* as well, and whether this effect is facilitated by the insulin signaling pathway. A *SgNPC1b* knockdown did not affect transcript levels of insulin-related peptide in the locust brain (Supplementary Figure S10), although this result does not exclude possible effects on actual insulin-related peptide release from the insulin-producing cells of the brain. Further investigation is therefore required to find out whether *SgNPC1b* depletion might have affected the insulin signaling pathway in *S. gregaria*.

Our data show that *SgNPC1b* performs a crucial function in the midgut. Furthermore, if compensatory mechanisms were available, either alternative sterol transporters or the incorporation of alternative sterols or non-sterol lipids in the cellular membranes, then these mechanisms were insufficient to rescue the knockdown of *SgNPC1b* in a timely fashion. In addition to *SgNPC1b*, several *SCP-x* and *SCP2* transcripts were identified in the *S. gregaria* midgut. However, none of these putative sterol transporters were upregulated upon feeding. Functional characterization of these proteins is therefore necessary to determine their possible roles in *S. gregaria*. Our data suggest that SCPs are either unable to compensate for reduced SgNPC1b function or are not involved in sterol uptake in *S. gregaria*. Finally, it cannot be excluded that SgNPC1b performs another crucial function, for example, the uptake of a different essential nutrient. This was suggested earlier by Voght et al., although so far no direct support for this hypothesis was published (Voght et al., 2007). Therefore, investigating the substrate specificity of SgNPC1b as well as the exact sterol depletions caused by the knockdown of SgNPC1b may contribute to a better understanding of its role in midgut physiology. In summary, we hypothesize that injection of *dsNPC1b* reduced dietary sterol uptake in the midgut and may have affected membrane integrity, resulting in reduced weight gain, impaired molting, and death. Additionally, we suggest that a *SgNPC1b* knockdown affected ecdysteroid biosynthesis, probably more indirectly (through an effect on weight gain and metabolism) than directly (through a lack of cholesterol as ecdysteroid precursor). Overall, our experiments were the first to demonstrate the crucial role and insecticidal target potential of NPC1b in an hemimetabolan (orthopteran) insect species.

## 5 Conclusion

The midgut transcriptome data presented here provide a useful and promising resource for studying the midgut physiology of *S. gregaria*, a socio-economically important pest insect. Furthermore, most midgut gene expression analyses available in the literature are exploratory or look at changes upon infection or exposure to xenobiotics in holometabolan orders, such as Diptera, Lepidoptera or Coleoptera. By contrast, studies focusing on the temporal changes in gene expression depending on food uptake and digestion, such as the one presented here, are still underrepresented. The gene enrichment patterns we observed are consistent with the polyphagous herbivorous lifestyle of *S. gregaria*, which is supported by the identification of a wide array of putative digestive enzymes and detoxification systems. The *S. gregaria* midgut can react rapidly at the transcriptional level to the presence of food, since a broad range of intestinal genes are upregulated, including genes coding for putative digestive enzymes, nutrient transporters and detoxification enzymes. This study highlighted the significant upregulation of H^+^ V-ATPase subunit-a and Niemann-Pick 1b protein upon feeding. Their vital role in conjunction with their accessibility from the luminal side of the midgut make them promising insecticidal target candidates.

## Data Availability

The datasets presented in this study can be found in online repositories. The names of the repository/repositories and accession number(s) can be found below: https://www.ncbi.nlm.nih.gov/geo/, GSE226871.

## Footnotes

1
http://transdecoder.github.io

2
https://www.ebi.ac.uk/interpro/

3
http://hmmer.org/

4
https://services.healthtech.dtu.dk/service.php?SignalP

5
https://services.healthtech.dtu.dk/service.php?TMHMM-2.0

6
https://www.bioinformatics.babraham.ac.uk/projects/fastqc/

7
https://cran.r-project.org/

8
https://primer3plus.com/cgi-bin/dev/primer3plus.cgi

9
https://www.beetleatlas.org
