# Supplementary material for: Post-feeding transcriptomics reveals essential genes expressed in the midgut of the desert locust
Source: Front Physiol. 2023 Aug 10;14:1232545. doi: 10.3389/fphys.2023.1232545 (PMC10484617; doi:10.3389/fphys.2023.1232545)
Supplement: Supplementary file 5 [file DataSheet1.docx]

Supplementary Material

Post-feeding transcriptomics reveals essential genes expressed in the midgut of the desert locust

Joachim Van Lommel^1,†^, Michiel Holtof^1,†^,Laurentijn Tilleman^2^, Dorien Cools^1^, Seppe Vansteenkiste^1^, Daria Polgun^1^, Rik Verdonck^1,3^, Filip Van Nieuwerburgh^2^ and Jozef Vanden Broeck^1^

^1^Molecular Developmental Physiology and Signal Transduction Lab, University of Leuven, Department of Biology, Leuven, Belgium

^2^NXTGNT, Ghent University, Department of Pharmaceutics, Ghent, Belgium

^3^ Environmental Biology, Hasselt University, Centre for Environmental Sciences, Hasselt, Belgium

†: These authors contributed equally to this work and share first authorship

***Correspondence:**Jozef Vanden Broeck
jozef.vandenbroeck@kuleuven.be

# Supplementary Materials and Methods

Protein sequences of H^+^ V-ATPase subunit-a and NPC1b orthologs from several other insect species were obtained from NCBI (Suppl. Table 10 and 11). Selected protein sequences were aligned using the Clustal Omega algorithm in BioEdit version 7.0.5.3 (Hall, 1999). Conserved protein domains were identified using NCBI conserved domain search (Lu *et al.*, 2020). A maximum likelihood phylogenetic analysis was performed on this multiple sequence alignment using IQTREE (version 1.6.12, LG+I+G4 substitution model, 1000 SH-aLRT replicates and 1000 ultrafast bootstrap replicates) (Trifinopoulos *et al.*, 2016). Phylogenetic trees were visualized using Interactive Tree of Life v6 (iTOL) (Letunic and Bork, 2021).

# Supplementary Figures and Tables

## Supplementary Figures


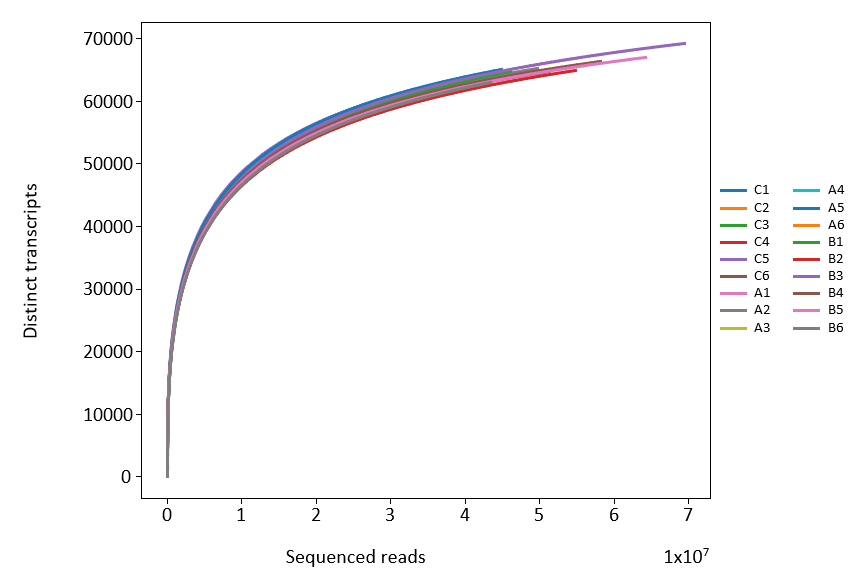


**Supplementary Figure 1.** **Sequencing coverage of the *S. gregaria* midgut reference transcriptome.** The number of distinct transcripts (y-axis) is plotted in function of the total amount of sequenced reads (x-axis). Colored lines represent all sequenced samples of the RNA-Seq experiment: A1-A6, 10 minutes after feeding; B1-B6, 2 hours after feeding and C1-C6, 24 hours after feeding.


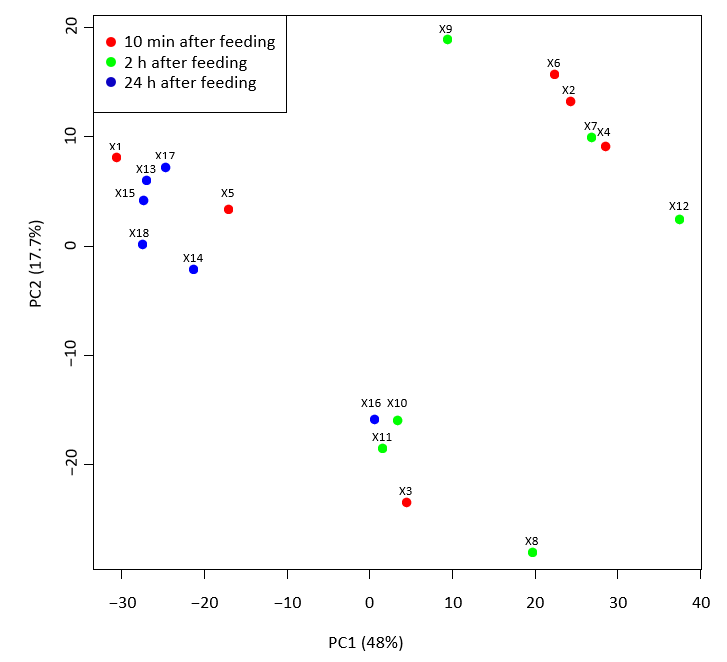


**Supplementary Figure 2. Principal component analysis (PCA) on log2 transformed counts of all samples**. Principal component 1 (PC1) and 2 (PC2) are indicated on the x- and y-axis, respectively. Transcript abundance was compared between three conditions: 10 minutes after feeding (10 min, red dots, sample x1-x6), 2 hours after feeding (2 h, green dots, sample x7-x12) and 24 hours after feeding (24 h, blue dots, sample x13-x18).

**Supplementary Figure 3**. ***SgVAHa1* consensus nucleotide and amino acid sequences.** The *SgVAHa1* nucleotide sequence contains a full open reading frame. Start and stop codon are highlighted in green and red, respectively. Primers for RT-qPCR are in bold and T7-linked primers are highlighted in teal. Both amplicons are underlined. A total of 14 amino acid residues previously described to be crucial for H^+^ V-ATPase subunit-a function were identified in SgVAHA1 and highlighted in black.

***SgVAHa1* nucleotide sequence**

TTCAGCAATCACGTTGGTAATCTCATATTGCCGACGAATGATAGGTACTAAACGCTAAAAGATCTGTGAACAGCCAGCAGC**TATTACTGTCGCGTCGCATC**TAAATACTGCCGCTTCTGCATCGGACGGTTGTGGAAGAAATCATCGCTCAGTGAAGCGCACGGAAGAAAAACAGCAGCCATGGGGGC**GATGTTTCGGAGCGAGAAGA**TGGCCTTGTGCCAGCTCTTCATCCAGCCTGAAGCCGCCTACGCTTCTGTGTCCGAGCTGGGAGAATTGGGCATCGCTCAGTTTCGGGATCTAAATCATAATGTGAATGCCATGTTGCGCAAGTTTGTTAACGAAGTTCGACTATGTGATGAGCTTGAACGCAAGCTACGATTTTTTGAGGCTGAGATAATCAAGGATGAAGTACCAATTCCTGATGTAGAGGACAACCCAAAGGCACCTAACCCAAGAGAGCTTGTCGATCTGGAGGCAAAATTTGAGCAAACAGAGAATGAATTACTGGAACTGAGTCAGAATGCAGTGAACCTGAAGCAGAACTTCCTAGAATTGACAGAACTGAAGAATGTGCTGGATAAGGCAGAGGGATTTTTTAAGAATCTGGAAGTTGCAAGTGCCTCTGACCTGCAGACTAGGGCACTCATGCAGGATGAGCCTGACAGTGGGAACCCTGATAAGGGTAGCCTGGGATTTGTTGCTGGAGTTGTACCTAAAGCGAAGGTTCCAGGATTTGAGAGAATGCTATGGAGAATATCACATGGAAATGTGTTCTTGCGACAGGCTGACTTAGAAGAACCCTTAGAAGAGCCAAAGACAGGCAACATGGTACAGAAGACAGTGTTTGTGGCCTTCTTTCAAGGTGAACAGTTGAGACTCCGGGTGAAAAAAGTGTGTACAGGTTTCCATGCAGCACTGTACTCATGTCCAACTGCTGCAGAAGAGCGTGCTGATATGTTATCTGGTGTCCGCACCCGCCTTCAAGACCTAACAGTTGTTCTAAATCAAACTAAAGATCACAGGCAGAGGGTACTACAGTCTGTTGCTAAAGAGCTGAACAGATGGACAATGATGGTACGCAAGATGAAGGCCATTTACCATACTCTCAATGGTTTCAATATGGATGTCACAAGCAGATGCCTTATTGCAGAGTGCTGGGTGCCTGTGAATGACCTACCAAGGATGACTCAAGCACTGCAAGATGGTGGGGTTGCTTCTGGAAGTTCTGTAGCTTCATTTCTTAATATAATTGAAACGTCAGATACTCCACCAACATACGTTAGAACAAATAAGTTCACAGCAGGATTTCAAAACCTTATTGATGCATATGGATCCATAACATACCAAGAAGTTAATCCAGGGTTGTTTACAATTATTACATTCCCGTTCTTGTTTGCAATAATGTTTGGAGATTCTGGACATGGACTCATTCTGTTTGCTTTTGGATTGTATATGATTCTCACTGAAAAGCAGCACTTGAAAAAGAAAATTACAAATGAGATTTGGGGCATCTTCTTCGCTGGACGTTACATTATAGTTTTAATGGGAATATTTTCTGTTTATACTGGACTAATATACAATGATGTTTTCTCAAAGTCTGTGAACATCTTTGGTAGCAATTGGAAAGTCTCCTATAATAGTTCAACTCTCAGATCAAATGATGAACTTCAGCTCAATCCAGCAACAGATTATGGTGATAACATATATCCCTTAGGCATGGATCCAGCTTGGCAGTTGGCTGAAGCAAACAAGATTATGTTCCTGAACTCATACAAAATGAAGTTATCCATAATATTTGGTTTCTTTCATATGGGATTTGGAGTGACTCTTGGTGTTGTGAACCATATCCACTTTCGTAAGCCAATCAACATCCTCCTTGAAACTGTACCCCAATTTCTTTTCCTACTGTTGCTGTTTGGATACTTGGTCTCACTTATGTTTGCTAAATGGATTCTATATGGAGCAAAAAATGAACTGTTGACAAGTGAACATTGTGCCCCAAATGTGCTCATCACGTTCATCAATATGCTCCTTTTCAAGAAATCTGAAGCAGTGTGTAATAGTGAAGGTGAAGACTGCTGTGATCCATACTTTTTTAATAACCAAAGAATGGTACAGATAGTCATGGTACTTGTGGCACTCCTTTGCATTCCGTGGCTTCTTCTGGCCAAACCAATATATATAATTCGTTCCCAGAAGAAAAACAGACAGAAAGTCCCCTCATCAGAAAATGCTGAGGAAAATGGAACCATTGAAATGGGGCATAGAGATGAAGAGGCTGGACCAGTGACTTCTTCAGAAGGAAATCATGATGATCACGAGGGTGGCGAAACTAGTGATATCTTCATTTACCAGGCCATACACACAATTGAGTATATCCTTGGAACCATATCACACACTGCCTCTTACTTGCGGCTGTGGGCTCTTTCTCTTGCACATTCCCAACTGTCTGAGGTGCTGTGGTCCAAAATTTTCCACATAGCTCTAGCGATGGATATAGGCTATGGTGGGGGAGTTTTAATATATGTACTATTCTGGGCATGGTCTTTCCTTACTCTTTCCATCCTTGTACTGATGGAAGGCCTCTCAGCATTTCTTCACACATTGAGGCTGCACTGGGTGGAGTTTATGAGCAAATTTTATACAGGTACTGGCTACATTTTTACACCATTCTCCTTCAAATCTATTTTGGAACAAGAGGACAATGAAGGACCATAGAGAAATTCGCTGTATCAGTGTAAAATATTGTTTTATTTATTCCATGTAAGATGTTATGGTAAAATGAGAAGCAGTTTCAAATTTTTGGAACTGCGTAGAGAAACTGTTTATAAGAACAAAACTGTAATATAAATATCTGCATTTGATAACTCCATACTTTGGAATTCTTTCACATTAAAAGAGTAACTGGCACCGATGTAAGATAACAGGCACATCAAATAAACAAGAAAAACTTGTAATGTAATAAAAAAATAGAATAATTTTATTTGTCAGAATATTTTGTGTCGTTATACAATTTACTTAATTATTTTCTCTTTAACAACTTTTTTAACTCAGCACAACATTATACAATATGTACTACATAAAAAGTGACCACACTC

**SgVAHa1 amino acid sequence**

MGAMFRSEKMALCQLFIQPEAAYASVSELGELGIAQFRDLNHNVNAMLRKFVNEVRLCDELERKLRFFEAEIIKDEVPIPDVEDNPKAPNPRELVDLEAKFEQTENELLELSQNAVNLKQNFLELTELKNVLDKAEGFFKNLEVASASDLQTRALMQDEPDSGNPDKGSLGFVAGVVPKAKVPGFERMLWRISHGNVFLRQADLEEPLEEPKTGNMVQKTVFVAFFQGEQLRLRVKKVCTGFHAALYSCPTAAEERADMLSGVRTRLQDLTVVLNQTKDHRQRVLQSVAKELNRWTMMVRKMKAIYHTLNGFNMDVTSRCLIAECWVPVNDLPRMTQALQDGGVASGSSVASFLNIIETSDTPPTYVRTNKFTAGFQNLIDAYGSITYQEVNPGLFTIITFPFLFAIMFGDSGHGLILFAFGLYMILTEKQHLKKKITNEIWGIFFAGRYIIVLMGIFSVYTGLIYNDVFSKSVNIFGSNWKVSYNSSTLRSNDELQLNPATDYGDNIYPLGMDPAWQLAEANKIMFLNSYKM**K**LSIIFGFFHMGFGVTLGVVNHIHFRKPINILLETVPQFLFLLLLFGYLVSLMFA**K**WILYGAKNELLTSEHCAPNVLITFINMLLFKKSEAVCNSEGEDCCDPYFFNNQRMVQIVMVLVALLCIPWLLLAKPIYIIRSQKKNRQKVPSSENAEENGTIEMGHRDEEAGPVTSSEGNHDDHEGGETSDIFIYQAIHTIEYILGTISHTASYLRLWALSLAHSQ**L**SEVLWSKIFHIALAMDIGYGGGVLIYVLFWAWSF**L**TLSILVLM**E**GLSAFLHTLRLHWVEFMSKFYTGTGYIFTPFSFKSILEQEDNEGP

**Supplementary Figure 4**. ***SgNPC1b* consensus nucleotide and amino acid sequences.** The *SgNPC1b* nucleotide sequence contains the full open reading frame. Start and stop codon are highlighted in green and red, respectively. Primers for RT-qPCR are in bold and T7-linked primers are highlighted (*dsNPC1b1*, black; *dsNPC1b2*, gray). PCR amplicons are underlined. The 3 domains, including reference to their Pfam accession number, that are characteristic for NPC1 proteins were identified and highlighted: N-terminal domain (PF16414, amino acid residue 27-251, magenta), sterol-sensing domain (PF12349, amino acid residue 628-781, yellow) and patched domain (PF02460, amino acid residue 1066-1240, cyan).

***SgNPC1b* nucleotide sequence**

GGGAGGAGTCACGCAGGCCAGCAGCATCATGAACTCCGCCTGGCAGTGGATAGGACTAGTTTTGCTTCTCTGGGCAGGAACAACCATTGCGCAAGAAGAGAGTGAGGGTCACTGCGTATGGTA**CGGTGAATGTTACGATGACG**GCGCAAGCAACAAGTTCAACTGCTATACCAACATAACAGCACAGAACTTGACCGACAAAGACTCACTTGAATACTTAAAGCAAACATGTC**CCTTTCTGGTGGACCAAGAA**GCAAAATATGCTACTCTGTGCTGCGATGGGCCCATGCTCGTTTCGATGCAAGAGAAGCTGAGCGTGGCTGAGGGCCTGCTGAAGCGCTGTCCTACGTGCTTGAACAACTTCCGCAAGCACCTGTGTGGAATGACGTGCGACCCACATCAGAGCCGCTTTATGGAAGCAGCGTCCACGAAAATAAATGAAGAAACGCAAAAAGAGTACATCGACAAACTGATTGTATACCTCTCAAAAGATTACATGGAAGGAACGTTTAACTCTTGCCGTCAAGTTTTGATGCCTTCGAGTGGTGTTCCCGCACTTGAAGCCATGTGTGGTAGTTTCGGTGCCGCTGGATGTACTTACGATAGATGGTTCGCATACATGGGAGATGATGACACTCCATTAGTACCTTTCCCTATCGTTTATAACGAGACAGCACCAGATGGAATGGAACTATATAATCCAGACGTCCTTCCGTGCAATCAGTCGTATGAGAACGACTACGCCTGCAGCTGCGCCGACTGTGAGGAGAGCTGTCCCGCAGACATAACTGTTATAGACACAGGAGAGACGAATGAGGAATTCAAAATTGGAGAAGCCGACGGCGTCCTCGTTATAATGGCAATAATATATGGAATTTTCGCAATTGGTTTTCTCATCTCTGTGATTTTTGTATCTCAAAGTGACACAGCAGGTGGTTCACCTCCATCACTCATCTACAAGCTTTTCTTCGGAGGCATCCCATTATTCCAGACGACGTTAAGCAAACTGTTCAGAAAACTGGGATTAGTATTTGCCTCGTATCCAGGAGCAACAATATGCTTAACATCCTGGGCAGTTGTTGGCATGAGCTATGGCGCATTGTCGTTGATCGTCACGACTGACCGCATAGAGCTATGGGCTTCACCGACAAGTCAATCGAGGATCGAAAAAGATTACTTTGACACTCATTTCGGGCCCTTCTACAGAACTGAACAAGTGTTCATCAAGGCTCACGGTCTGGACAATATTATGTACGAAAGCGGTATGCAACCTGTGGAATTCGGGCCAGTTTTCAATAAAACTTTTCTGTTAACTGTCCTCGATTTGCAACAACAGATAATTCAGATCGGAGCTGGTGAAAGCTACGAGCTGAAGAATATCTGTAATGCACCATTGGCGACGGGTGAAGTGAAAACAGAAGACTGCCTTGTGCAAAGTATTTGGGGCTATCTGAAAAACAATGCAAGTTTGTTAGAAGATGATTCCTACTTGAGCATATTGTTAACGTGCATGCAGAACAACGTTGATTTTAGCTGTTTGGGCCCTTACGGCGGGCCCATTTTCGCTGGTTTGGCACTTGGTGGTTTTCCGGAAGATGCAAACGTTAATTACTCAAAGTATGCGTTGTCAACAGGTCTCTCACTGACATTTTTGGTCAACAATCACTTGGATAAGACAGATCTACAACCGGCTCTTGAATGGGAATCTAAGTTCATCCAGTTTTTGAAAGAATGGTCGGAGACAAAGATGCCCTCCTTCATGTCAATTGCTTTTTCGGCTGAACGGTCAATAGAAGACGAGCTTGCACGTGAATCTCAAGCAGAAATAGCAACTGTTGTTATCAGCTATGCTGTGATGTTTTTGTATATCACACTTGCCATTGGACGGTATAGATCTTCAAAAACAATACAGATTGATGGAAAATTTACTCTGGGAGTAAGCGGAATAATCATAGTACTTATGTCAGTATTATCATCTCTTGGGATATTTGGATATGCCGGAGTTGCAACAACATTGTTAACAATTGAGGTCATCCCTTTCCTTGTACTGGCCATTGGAGTTGACAACATATTCATCCTAGTACAGACACATCAGCGTAATCCGAGACAAAAAACTGAAAGCCATGAGGAACATGTTGCACGGACACTGGGTACAGTTGGACCCAGTATGCTACTCACAAGTTTATCAGAGACATGCTGCTTTCTTATAGGTGCTCTGTCAAACATGCCTGCTGTCAATACATTTGCTCTTTATGCATCTGTAGCTACAATACTAAATTTCATTTTCCAAATAACATGCTTTGTAAGCATTATGACTTTGGATGATAAACGACAGTCAGTAAACAGATATGATATTTGCTGCTGTGTTAAGTCAGAAAAGGCAGTTGATGATACACCTTCTGAAGACTTTCTGTACCTGTTATTCAAGTCATATTACAGTAAATTTCTCCTCAATCGCTTTGTAAAAATAGTGGTTGTTCTCCTATTTTCATTCTGGCTGTGTTCGAGTTTAGTGCTAATACCACGCATTGAGGCTGGATTAGACCAAGAAATTGCAGTGCCAACAGATTCATATGTCCGATCATATTTTGAGTACATGAAAGATGTTCTCTCCATGGGTCCAAATGTGTATTTTGTAGTTAAAAGTGGCCTGAACTACAGTAATGAAAATGTTCAAAATTTGTTCTGTGGTGGCCTGAACTGCTATGCAGATTCCCTAACAACTCAAATTACTCGTGCTTCAAAGTTACCAGACAGATCATACATAACTACAAGTGCGTCATCATGGCTTGATGATTATTTTGACTGGATAAACTCTGGCAACTGCTGTCAAGATGATAAAGGCACAGAATGTTCTGAAACACCAAGACCAACACCGAAGGATTTTGAAACGTATTTACCACAGTTTTTGCTAGCAAATCCTTCACAAGAATGTGCTAAGGGAGGACACGCAGCATATGCAGAGGGCTTGAATTATGTTTTGAATAAAGATGGTCTGGCAATTGCCATGGACAGTTACTTCATGACTTATCACACTGTTCTGAGAACTTCTGAGGAGTACACAAATGCTCTGAGATCAGCAAGAGAAATAGCTAGTAGTATTACTACAATGTTACAGAGTTATCTAAGACCCACTGTGAATGTTACTACAGAAAATGATACAGATTCGACTAATTGGTTTGAAGTTGACAATGACACGTATATATCAAATGTTTCAAATGTTGAAGTGTTTCCATACAGTGTCTTCTATGTGTTTTATGAACAGTATCTCACAATTACAGGAGATACAATAAACTCTATTGGCTACTCTTTGTCGGCAGTGTTCATAGTGTCATTTATTCTCATGGGCTTTAACATATTCTCATCTGTCATCATACTAATTATGGTTACCATCATTGTGACTAACCTCGCTGCTTTCATGGTATGGTGGGATGTACCACTAAATGCAGTCTCCCTTGTAAATCTTGTTGTTGGTGTTGGTATAGCTGTGGAATTCTGCAGTCACATTATACGGGCTTACACTGTTTCCACTCTTTCAGGGAGCAATAAACGAGCAGCAGATGCTCTTACTAAGATTGGCAGCTCAGTACTGTCAGGAATTACGCTGACAAAAATTGTTGGTATTTTTGTGCTGGCTTTTGCTAAGACTGAGATCTTCCAAGTCTTCTATTTCCGAATGTATCTTGGAATCGTGCTGATAGGAGCAGCACATGGGCTGATTTTTCTTCCAGTGTTGCTTAGCTATATTGGACCAAACAAGAACACCAATCCAACAATTGAAGACATTACAAGATCTACA**TAA**CCAGTTACCAGTGAAATGGTATCATCGATCAACATATACCACATGTAACTGGTGCAAAGTGAACAAATAAAGAGGCAGCTGTGGATATGATATCTGTATGTGCATAACCTGTGTCCAGACTCCTAAGATGGAGCACATTTAACAAATGAACTGATGAAATCTGTGTAGCACTTTTGAAGGTTTCCGAGGTGTCACATTAGTATGTGTGTGTGTGTGTGTGTGTGTGTGTGTGTGTGTGTGTGAGAGAGAGAGAGAGAGTGAGAGAGAGAGAGAGAGAGAG

**SgNPC1b amino acid sequence**

MNSAWQWIGLVLLLWAGTTIAQEESEGHCVWYGECYDDGASNKFNCYTNITAQNLTDKDSLEYLKQTCPFLVDQEAKYATLCCDGPMLVSMQEKLSVAEGLLKRCPTCLNNFRKHLCGMTCDPHQSRFMEAASTKINEETQKEYIDKLIVYLSKDYMEGTFNSCRQVLMPSSGVPALEAMCGSFGAAGCTYDRWFAYMGDDDTPLVPFPIVYNETAPDGMELYNPDVLPCNQSYENDYACSCADCEESCPADITVIDTGETNEEFKIGEADGVLVIMAIIYGIFAIGFLISVIFVSQSDTAGGSPPSLIYKLFFGGIPLFQTTLSKLFRKLGLVFASYPGATICLTSWAVVGMSYGALSLIVTTDRIELWASPTSQSRIEKDYFDTHFGPFYRTEQVFIKAHGLDNIMYESGMQPVEFGPVFNKTFLLTVLDLQQQIIQIGAGESYELKNICNAPLATGEVKTEDCLVQSIWGYLKNNASLLEDDSYLSILLTCMQNNVDFSCLGPYGGPIFAGLALGGFPEDANVNYSKYALSTGLSLTFLVNNHLDKTDLQPALEWESKFIQFLKEWSETKMPSFMSIAFSAERSIEDELARESQAEIATVVISYAVMFLYITLAIGRYRSSKTIQIDGKFTLGVSGIIIVLMSVLSSLGIFGYAGVATTLLTIEVIPFLVLAIGVDNIFILVQTHQRNPRQKTESHEEHVARTLGTVGPSMLLTSLSETCCFLIGALSNMPAVNTFALYASVATILNFIFQITCFVSIMTLDDKRQSVNRYDICCCVKSEKAVDDTPSEDFLYLLFKSYYSKFLLNRFVKIVVVLLFSFWLCSSLVLIPRIEAGLDQEIAVPTDSYVRSYFEYMKDVLSMGPNVYFVVKSGLNYSNENVQNLFCGGLNCYADSLTTQITRASKLPDRSYITTSASSWLDDYFDWINSGNCCQDDKGTECSETPRPTPKDFETYLPQFLLANPSQECAKGGHAAYAEGLNYVLNKDGLAIAMDSYFMTYHTVLRTSEEYTNALRSAREIASSITTMLQSYLRPTVNVTTENDTDSTNWFEVDNDTYISNVSNVEVFPYSVFYVFYEQYLTITGDTINSIGYSLSAVFIVSFILMGFNIFSSVIILIMVTIIVTNLAAFMVWWDVPLNAVSLVNLVVGVGIAVEFCSHIIRAYTVSTLSGSNKRAADALTKIGSSVLSGITLTKIVGIFVLAFAKTEIFQVFYFRMYLGIVLIGAAHGLIFLPVLLSYIGPNKNTNPTIEDITRST


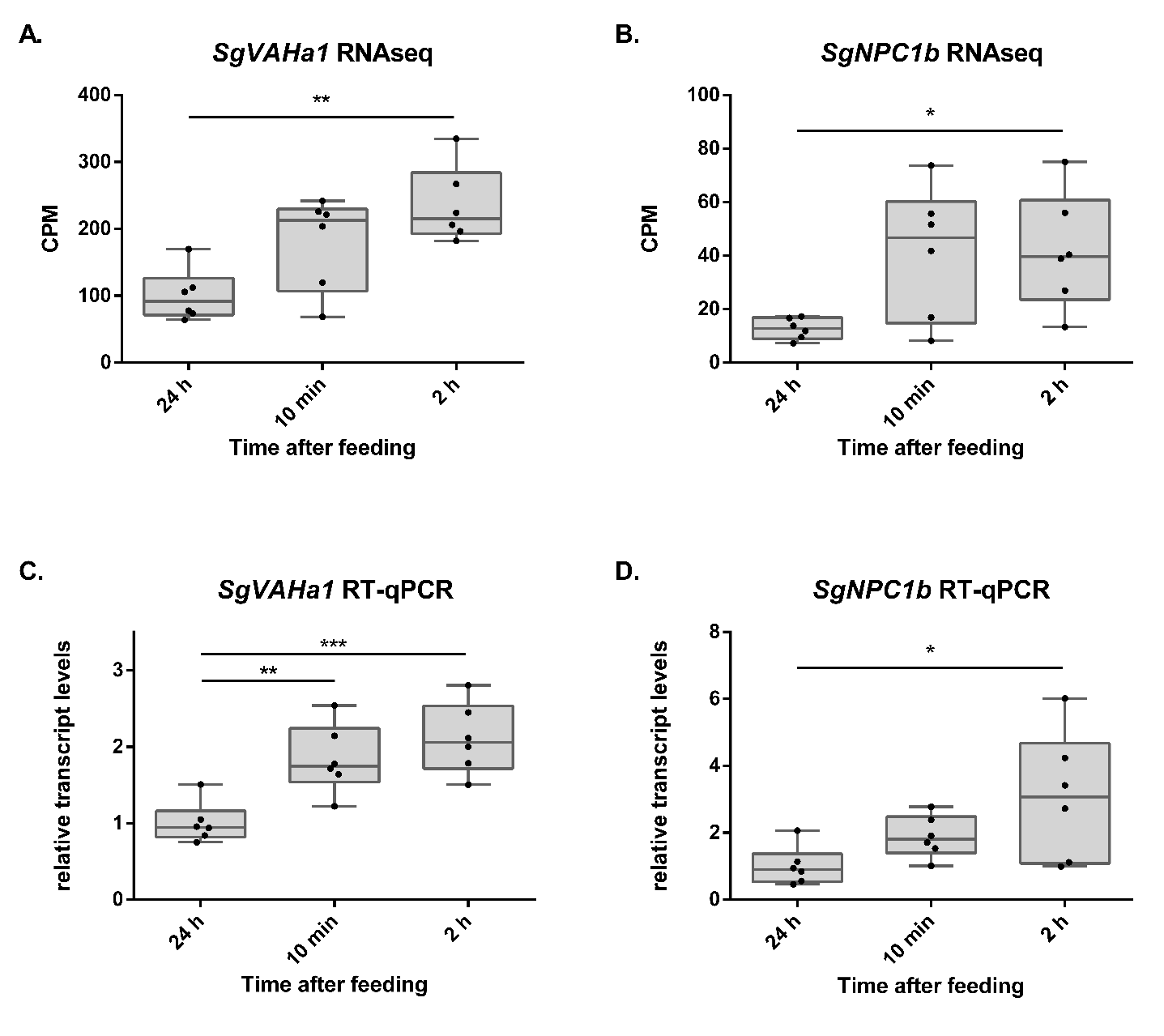


**Supplementary Figure 5.** **Validation of changes in *SgVAHa1* and *SgNPC1b* expression in the *S. gregaria* midgut upon feeding.** Abundance of transcripts encoding *SgVAHa1* (panel A) and *SgNPC1b* (panel B) were determined by Illumina® deep sequencing of mRNAs (RNA-seq) in three conditions: 24 hours after feeding (24 h), 10 minutes after feeding (10 min) and 2 hours after feeding (2 h). The transcript abundance is indicated on the y-axis and is expressed as the log_2_-transformed trimmed mean of M-values (TMM)-normalized counts per million (CPM). These changes were validated using RT-qPCR for both *SgVAHa1* (panel C) and *SgNPC1b* (panel D). Data are represented by boxplots containing the upper and lower quartile, while the whiskers indicate the minimum and maximum. The median is indicated by the gray line in the center of the box. Relative transcript levels (panel C and D) were normalized using *SgGAPDH* and *SgRP49* as reference genes. Statistical significance is indicated by an asterisk (*: p ≤ 0.05, **: p ≤ 0.01, ***: p ≤ 0.001). **A.** Transcript abundance of *SgVAHa1* was significantly higher in the 2 h condition compared to the 24 h condition (one-way ANOVA with Holm-Šídák’s post-hoc test, p=0.0208). **B.** Transcript abundance of *SgNPC1b* was significantly higher in the 2 h condition compared to the 24 h condition (one-way ANOVA with Holm-Šídák’s post-hoc test, p = 0.0469). **C.** Relative transcript levels of *SgVAHa1* were significantly higher in the 10 min and 2 h condition compared to the 24 h condition (one-way ANOVA with Holm-Šídák’s post-hoc test, 10 min vs. 24 h: p = 0.0057, 2 h min vs. 24 h: p = 0.0008). **D.** Relative transcript levels of *SgNPC1b* were significantly higher in the 2 h condition compared to the 24 h condition (one-way ANOVA with Holm-Šídák’s post-hoc test, p = 0.0027).

**Supplementary Figure 6**. **Multiple sequence alignment of H^+^ V-ATPase subunit-a isoforms of *S. gregaria*.** The H^+^ ATPase subunit-a amino acid sequences from three *Schistocerca gregaria* isoforms (*SgVAHa1*, accession number XP_049832128.1; *SgVAHa2*, accession number [XP_049843022.1](https://www.ncbi.nlm.nih.gov/protein/XP_049843022.1?report=genbank&log$=prottop&blast_rank=1&RID=ZPWVC9AP016) and *SgVAHa3*, accession number [XP_049831722.1](https://www.ncbi.nlm.nih.gov/protein/XP_049831722.1?report=genbank&log$=prottop&blast_rank=1&RID=ZPWP5MP0016)) were compared to three insect sequences, namely *Locusta migratoria* (Lm, accession number UHW40945.1), *Manduca sexta* (Ms, accession number [XP_030029557.1](https://www.ncbi.nlm.nih.gov/protein/XP_030029557.1?report=genbank&log$=prottop&blast_rank=35&RID=KS2DJ7DV01R)) and *Drosophila melanogaster* (DmVHa100.4, accession number NP_650720.1). The seven transmembrane domains (I-VII) as well as the coiled coil domain are indicated with black boxes. A total of fourteen amino acid residues previously described to be crucial for H^+^ V-ATPase subunit-a function were identified and indicated with red arrowheads.

**10 20 30 40 50 60 70 80**

**....|....|....|....|....|....|....|....|....|....|....|....|....|....|....|....|**

***SgVAHa1***  ------------MGAMFRSEKMALCQLFIQPEAAYASVSELGELGIAQFRDLNHNVNAMLRKFVNEVRLCDELERKLRFF

***SgVAHa2***  ------------MGSLFRSEEMTLCQLFLQSEAAYACVSELGELGLVQFRDLNPDVNAFQRKFVNEVRRCDEMERKLRYL

***SgVAHa3***  ------------MGAMFRSEEMALCQLFIQPEAAYPSVSELGELGIAQFRDLNNDVNAFQRKFVNEVRRCDELERKLRYI

***LmVAHa1***  ------------MGSLFRSEEMALCQLFLQSEAAYACVSELGELGLVQFRDLNPDVNAFQRKFVNEVRRCDEMERKLRYL

***MsVAHa***  ------------MGSLFRSEEMTLCQLFLQSEAAYACVSELGELGLVQFRDLNPDVNAFQRKFVNEVRRCDEMERKLRYL

***DmVHa100.4*** MSKWWSCGSNQESNSIFRSEVMSLVQMYLQPEAAYDTIAALGEVGCVQFRDLNAKINAQQRKFIGEVRRCDELERRIRYV

Coiled coil

**90 100 110 120 130 140 150 160**

**....|....|....|....|....|....|....|....|....|....|....|....|....|....|....|....|**

***SgVAHa1***  EAEIIKDEVPIPDV-EDNPKAPNPRELVDLEAKFEQTENELLELSQNAVNLKQNFLELTELKNVLDKAEGFFKNLE-VAS

***SgVAHa2***  EKEIRKDGIPMLDT-GESPEAPQPREMIDLEATFEKLENELSEVNQNAEALKRNFLELTELKHILRKTQVFFDEHEGGAN

***SgVAHa3***  EAEIVKDGVSVPEI-DEIPKAPNPREIIDLEANLEKTENEIMELSQNAVNLKLNFLELTEMRHVLEKTEGFFTEQE-GVG

***LmVAHa1***  EKEIRKDGIPMLDT-GESPEAPQPREMIDLEATFEKLENELSEVNQNAEALKRNFLELTELKHILRKTQVFFDEHEGGAN

***MsVAHa***  EKEIRRDGIPMLEIPGECPEAPQPREMIDLEATFEKLENELREVNQNAEALKRNYLELTELKHILRKTQVFFDEMADPSR

***DmVHa100.4*** TAELNKEGHKVLDLMDDFPPAPQPREIIDLELHLEKTETEILELAANNVNLQTSYLELSEMIQVLERTDQFFSDQE----

**170 180 190 200 210 220 230 240**

**....|....|....|....|....|....|....|....|....|....|....|....|....|....|....|....|**

***SgVAHa1***  ASDLQTRALMQDEPDSGN----PDKGSLGFVAGVVPKAKVPGFERMLWRISHGNVFLRQADLEEPLEEPKTGNMVQKTVF

***SgVAHa2***  ATESMTRALISDDSIARQAP--SGPVQLGFVAGVILRERIPAFERMLWRACRGNVFLRQAEIETPLEDPSNGDAVYKSVF

***SgVAHa3***  ASDSLTRALIQEESSTQAGASASARGRLGFVAGVVPRERVPAFERMLWRISRGNVFLRQVGLDQPLDEPNTGNSVYKTVF

***LmVAHa1***  ATESMTRALISDDSIARQAP--SGPVQLGFVAGVILRERIPAFERMLWRACRGNVFLRQAEIETPLEDPSNGDAVYKSVF

***MsVAHa***  EEEQVT--LLGEE--GLMAG--GQALKLGFVAGVILRERIPAFERMLWRACRGNVFLRQAEIDTPLEDPSSSDQVYKSVF

***DmVHa100.4*** -SHNFDLNKMGTHRDPEKSN-----GHLGFVAGVISREREYAFERMLWRISRGNVFVRRCDVDVALTDPKTGNVLHKSVF

**250 260 270 280 290 300 310 320**

**....|....|....|....|....|....|....|....|....|....|....|....|....|....|....|....|**

***SgVAHa1***  VAFFQGEQLRLRVKKVCTGFHAALYSCPTAAEERADMLSGVRTRLQDLTVVLNQTKDHRQRVLQSVAKELNRWTMMVRKM

***SgVAHa2***  IIFFQGDQLKTRVKKICEGFRATLYPCPEAPADRREMAMGVMTRIEDLNTVLGQTQDHRHRVLVAAAKNIKNWFIKVRKI

***SgVAHa3***  VAFFQGEELKSRVKKVCAGFHASLYPCPSVAGERADMLAGVRTRLQDLTLVLNQTQDHRQRVLVGVARELNNWMVMVRKM

***LmVAHa1***  IIFFQGDQLKTRVKKICEGFRATLYPCPEAPADRREMAMGVMTRIEDLNTVLGQTQDHRHRVLVAAAKNIKNWFIKVRKI

***MsVAHa***  IIFFQGDQLKTRVKKICEGFRATLYPCPESPADRREMAMGVMTRIEDLNTVLGQTQDHRHRVLVAAAKNIKNWFVKVRKI

***DmVHa100.4*** VVFFQGDQLQARIRKVCTGFHAHMYPCPSSHSERQEMVKNVRTRLEDLQVIINQTSDHRTCVLQAALKQLPTWSAMVKKM

**330 340 350 360 370 380 390 400**

**....|....|....|....|....|....|....|....|....|....|....|....|....|....|....|....|**

***SgVAHa1***  KAIYHTLNGFNMDVTSRCLIAECWVPVNDLPRMTQALQDGGVASGSSVASFLNIIETSDTPPTYVRTNKFTAGFQNLIDA

***SgVAHa2***  KAIYFTLNLFNLDVTQKCLIAECWVPLLDLETIQLALRRGTERSGSSVPPILNRMQTFEDPPTYNRTNKFTSAFQTLVDA

***SgVAHa3***  KAIYHTLNSFNMDVTNKCLIAECWVPVSDLPKLRKALQDGSKACGSSIPSFLNAIETSETPPTFNRTNKFTAGFQNLIDA

***LmVAHa1***  KAIYFTLNLFNLDVTQKCLIAECWVPLLDLETIQLALRRGTERSGSSVPPILNRMQTFEDPPTYNRTNKFTSAFQTLVDA

***MsVAHa***  KAIYHTLNLFNLDVTQKCLIAECWVPALDLETIQLALRRGTERSGSSVPPILNRMETLEDPPTYNRNNKFTQAFQNLIYA

***DmVHa100.4*** KGIYHTLNLFNVDLGSKCLIGEGWVPKRELELVEVALAAGSASVGSTVPSFINVLDTKKEPPTHFRTNKFTRGFQNLIDA

I

II

**410 420 430 440 450 460 470 480**

ii

**....|....|....|....|....|....|....|....|....|....|....|....|....|....|....|....|**

***SgVAHa1***  YGSITYQEVNPGLFTIITFPFLFAIMFGDSGHGLILFAFGLYMILTEKQHLKKKITNEIWGIFFAGRYIIVLMGIFSVYT

***SgVAHa2***  YGVASYREVNPAPYTIITFPFLFAVMFGDTGHGLIMALFGFWMVLKEKPLAAKKSDSDIWNIFFGGRYVVLLMGLFSMYT

***SgVAHa3***  YGAISYQELNPALYTIITFPFLFAIMFGDAGHGLIMFAFGLYMVLTEKKHMKQKSTNEIWNIFFAGRYIILLMGTFSMYT

***LmVAHa1***  YGVASYREVNPAPYTIITFPFLFAVMFGDTGHGLIMALFGFWMVLKEKPLAAKKSDSDIWNIFFGGRYVVLLMGLFSMYT

***MsVAHa***  YGVATYREVNPAPYTIITFPFLFAVMFGDLGHGALMAAFGFWMCYKEKPLQAKKIDSEIWNIFFGGRYIILLMGLFSMYT

***DmVHa100.4*** YGIAGYREVNPGLYTCITFPFLFAVMFGDMGHGTILFLLGLWMVIDEKRLSKKR-GGEIWNIFFAGRYIIMLMGLFAMYT

**490 500 510 520 530 540 550 560**

**....|....|....|....|....|....|....|....|....|....|....|....|....|....|....|....|**

***SgVAHa1***  GLIYNDVFSKSVNIFGSNWKVSYNSSTLRSNDELQLNP-ATDYGDNIYPLGMDPAWQLAEANKIMFLNSYKMKLSIIFGF

***SgVAHa2***  GLIYNDVFSKSLNIFGSYWKVEYNESTLRHSKELQLDP-AKEFLQYPYPFGMDPVWQLAEN-KIIFMNSYKMKISIILGV

***SgVAHa3***  GLIYNDFFSKSVNIFGSNWNVSYDESTLKANHELQLSP-KNDYGDSIYPLGMDPAWQLAEANKIMYLNSYKMKLSIIFGV

***LmVAHa1***  GLIYNDVFSKSLNIFGSYWKVEYNMSTLRTNKELQLDP-AKEFLQYPYPFGMDPVWQLAEN-KIIFMNSYKMKISIILGV

***MsVAHa***  GLIYNDIFSKSLNIFGSSWRQNYNASTLTENKLLQLNPDSPDYLQYPYPFGIDPVWQLAEANKIIFMNAYKMKISIIIGV

***DmVHa100.4*** GFHYNDIFSKSINVFGTRWVNVYNRTTVLTNPTLQLNP--SVATRGVYPMGIDPIWQSASN-KIIFLNTYKMKLSIIFGV

III

IV

V

**570 580 590 600 610 620 630 640**

**....|....|....|....|....|....|....|....|....|....|....|....|....|....|....|....|**

***SgVAHa1***  FHMGFGVTLGVVNHIHFRKPINILLETVPQFLFLLLLFGYLVSLMFAKWILYGAKN---------ELLTSEHCAPNVLIT

***SgVAHa2***  LHMLFGVLLSLWNHLYFKNSINIICEFVPQIIFLIFLFLYMCILMFVKWVNYGPT---FG------FTAGPACAPSILIT

***SgVAHa3***  LHMIFGVCLSVVNHVHFRKRINIVLEFLPQVLFLVLLFGYLVSLMFVKWVVYSAKN---------ELLTSEHCAPNVLIT

***LmVAHa1***  IHMLFGVLLSLWNHLYFKNSINIICEFVPQIIFLVFLFLYMCILMFVKWVNYGPT---FD------FTMGPACAPSILIT

***MsVAHa***  FHMLFGVCLSLWNHLYFKRRISVYVEFIPQILFLTLLFFYMVLLMFIKWTSYGPTPGAFGSQDPAIVKTSAYCAPSILIT

***DmVHa100.4*** LHMVFGVCMSVENFVFFKKYAYIILQFVPQVLFLLLMFGYMCFMMFYKWVKYSPTT--------DVEADTPGCAPSVLIM

V

VI

**650 660 670 680 690 700 710 720**

**....|....|....|....|....|....|....|....|....|....|....|....|....|....|....|....|**

***SgVAHa1***  FINMLLFKKSEAVCNSEGEDCCDPYFFNNQRMVQIVMVLVALLCIPWLLLAKPIYIIRSQKKNRQKVPSSENAEENG---

***SgVAHa2***  FINMVLFKG-----SVPPK-GCDEFMYSGQKGLQRFFVVLALLCVPWMLLAKPIVLIMRHRKAHQLLSSHPVPAENGMDA

***SgVAHa3***  FINMLLFKQAEPLCNSEGKDCCNIYIFESQGTVQQIMVFVALLCVPWLLLAKPLYIMCSRKKSRQQVSASENGDVNQ---

***LmVAHa1***  FINMVLFKG-----SVPPK-GCDEFMFSGQKGLQRFFVVLALLCIPWMLLAKPIVLIMRHRKAHQPLSSHPVPAENGMDA

***MsVAHa***  FINMMLFKT-----DANTRPQCDDTMYAGQLQLQKFFVIVALLCVPVMLFGKP-YFIMKEQKQRARQGHQPV--------

***DmVHa100.4*** FIDMVLFKT------ETALPGCDVNMFPIQKNLEMIFLVVALLCIPWILLGKPLYIKYQ-RRNRPAGPVEEVDEIVEKIE

**730 740 750 760 770 780 790 800**

**....|....|....|....|....|....|....|....|....|....|....|....|....|....|....|....|**

***SgVAHa1***  TIEMGHRDEEAGPVTSSEGNHDDHEGGETSDIFIYQAIHTIEYILGTISHTASYLRLWALSLAHSQLSEVLWSKIFHIAL

***SgVAHa2***  EVGSMSGTAHKDSTDGAPAPQS-SEDHDLGEIFIHQGIHTIEYVLGSVSHTASYLRLWALSLAHAQLSEVLWSMVMKNGL

***SgVAHa3***  GIELRQENEEVGAVTPADG-HDGHGEDDMTEVFIYQSIHTIEYILSTISHTASYLRLWALSLAHSQLSEVLWTMVLRMGL

***LmVAHa1***  EVGSMSGTVQKETTEGPPAPQS-SEDHDLGEIFIHQGIHTIEYVLGSVSHTASYLRLWALSLAHAQLSEVLWSMVMKNGL

***MsVAHa***  -----EGAAENGTAGGAPVPSSGHHDDDITEVFIHQGIHTIEYVLGSVSHTASYLRLWALSLAHAQLAEVAWNMLLRKGL

***DmVHa100.4*** VTTGKEIIITEVAEAHESGGHSEEDDEPMSEIWIHQAIHTIEYILSTISHTASYLRLWALSLAHAQLSEVLWTMVLAMGL

VII

**810 820 830 840 850 860 870**

**....|....|....|....|....|....|....|....|....|....|....|....|....|....|....|....**

***SgVAHa1***  AMDIGYGGGVLIYVLFWAWSFLTLSILVLMEGLSAFLHTLRLHWVEFMSKFYTGTGYIFTPFS-FKSILEQEDNEGP--

***SgVAHa2***  VVEG-WTGGFALWLVFAFWAVLTVGILVLMEGLSAFLHTLRLHWVEFQSKFYAGVGYSFQPFS-FEAILDSASQAPED-

***SgVAHa3***  TAEG-YTGAIILYIMFLAWSFLTVAILVLMEGLSAFLHTLRLHWVEFMSKFYSGAGYLFAPFS-FKNILEQDDNEQ---

***LmVAHa1***  VVEG-WAGGFALWMVFAFWAVLTVGILVLMEGLSAFLHTLRLHWVEFQSKFYSGVGYSFQPFS-FETILDSASHSAED-

***MsVAHa***  MSTD-FQGGIFLYIVFAGWAAISVSILVLMEGLSAFLHTLRLHWVEFQSKFYAGEGLSLPTGSRFEVILDSAGPGRGVN

***DmVHa100.4*** QMNG-YVGAIGLFFIFAVWEFFTIAIMVMMEGLSAFLHTLRLHWVEFMSKFYVGNGYPFTPFS-FKDILIVVEDD----


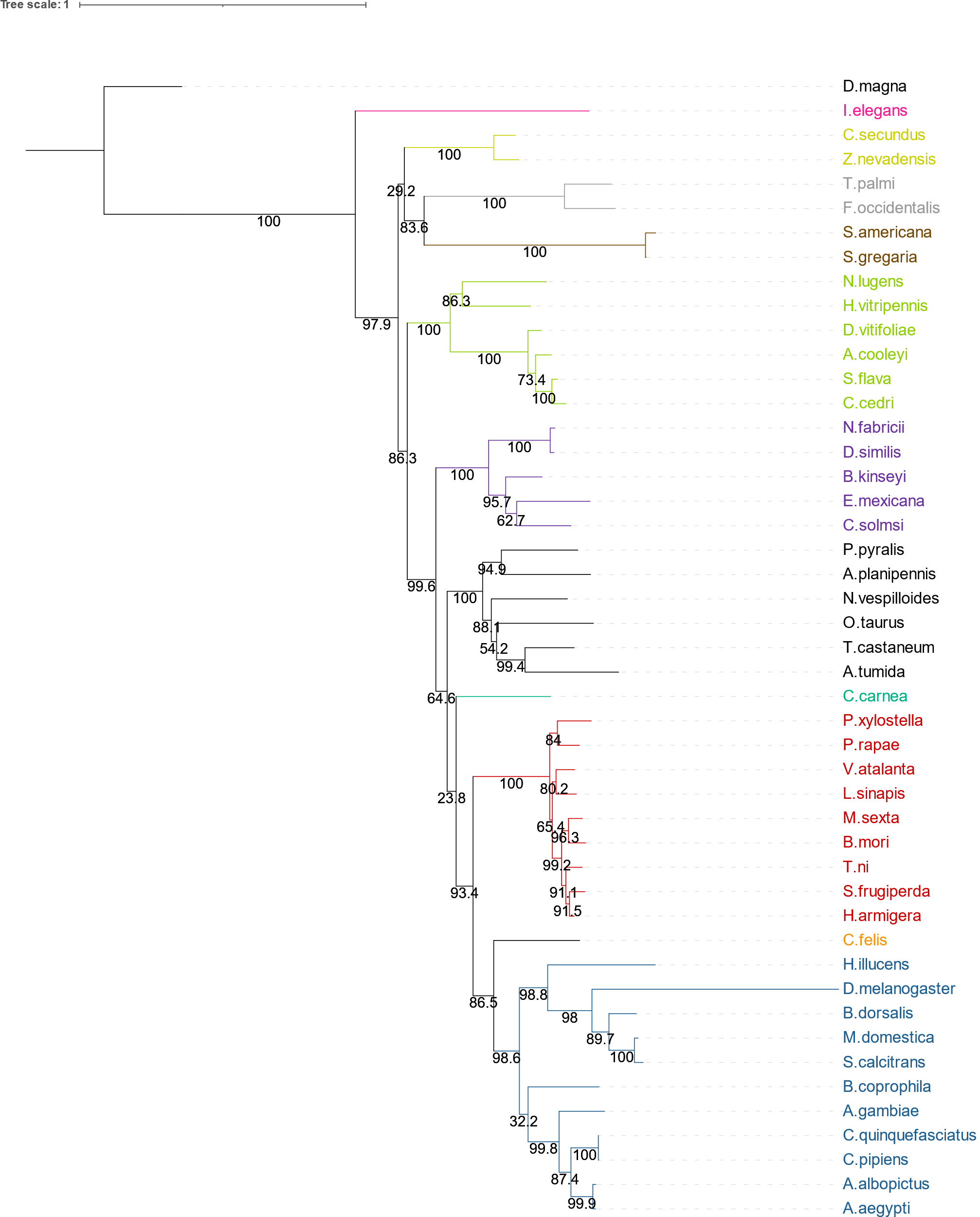


**Supplementary Figure 7**. **Phylogenetic analysis of H^+^ V-ATPase subunit-a proteins in various insect species.** Phylogenetic relationships of H^+^ V-ATPase 116 kDa subunit proteins obtained by Maximum Likelihood method. The branch lengths represent the evolutionary distance between clades and is expressed as the amino acid substitution rate per site. The branch length scale is indicated by the scale bar in the top left corner. The node support (%) is indicated at each node. Different insect orders are indicated with distinct colors (Coleoptera: black, Diptera: blue, Hemiptera: green, Hymenoptera: purple, Isoptera: yellow, Lepidoptera: red, Neuroptera: teal, Odonata: magenta, Orthoptera: brown, Siphonaptera: orange, Thysanoptera: grey). A sequence from the crustacean *Daphnia magna* was used as an outgroup to root the tree.

**Supplementary Figure 8**. **Multiple sequence alignment of NPC1b proteins with SgNPC1b as reference sequence.** The NPC1b amino acid sequence from *Schistocerca gregaria* (SgNPC1b) was compared to 2 insect sequences, namely *Helicoverpa armigera* (Ha, accession number QEP99645.1) and *Drosophila melanogaster* (Dm, accession number NP_608417.2) as well as *Homo sapiens* Niemann-pick C1-like 1 protein (HsNPC1L1, accession number AAI43757.1). The three domains characteristic for Niemann-pick C1 proteins are indicated by boxes: N-terminal domain (black box), sterol-sensing domain (dark grey box) and patched domain (light grey box).

**10 20 30 40 50 60 70 80**

**....|....|....|....|....|....|....|....|....|....|....|....|....|....|....|....|**

***SgNPC1b***  ----MNSAWQWIGLVLLLWAGTTIAQEESEGHCVWYGECYDDGASN------KFNCYTNITAQNLTDKDSLEYLKQTCPF

***HaNPC1b***  -----MKYLTTILCFLTLWCS-ANARCVVRGECYEVNGMAKPCHVD------MEAQPLIDGLTEEKAREVVEIFSSICPT

***DmNPC1b***  --------MKVIFATIWLIAG-AWSQSAEQLGCIWYG-QSHMIGAH------WVNKGDTNPARPLNSPTSEAIFAKRCPM

***HsNPC1L1*** MAEAGLRGWLLWALLLRLAQSEPYTTIHQPGYCAFYDECGKNPELSGSLMTLSNVSCLSNTPARKITGDHLILLQKICPR

N-terminal domain

**90 100 110 120 130 140 150 160**

**....|....|....|....|....|....|....|....|....|....|....|....|....|....|....|....|**

***SgNPC1b***  LVDQEAKY------ATLCCDGPMLVSMQEKLSVAEGLLKRCPTCLNNFRKHLCGMTCDPHQSRFMEAASTKINEETQKEY

***HaNPC1b***  FVVDDEGNRLPDDQILTCCTADQVIKTAESLTLAEGVLGRCPTCYRNFARQICEMNCAADQSRFLNATNTTAPDG--TVH

***DmNPC1b***  LYKEYKGE-SGEDELSLCCDAAQIETMESGLSQADGVFSRCPTCTRNMALTVCAMTCAKNHTLFLTAYNDTNDAG--VDY

***HsNPC1L1*** LYTGPNTQ--------ACCSAKQLVSLEASLSITKALLTRCPACSDNFVNLHCHNTCSPNQSLFINVTRVAQLGAGQLPA

**170 180 190 200 210 220 230 240**

**....|....|....|....|....|....|....|....|....|....|....|....|....|....|....|....|**

***SgNPC1b***  IDKLIVYLSKDYMEGTFNSCRQVLMPSSGVPALEAMCGSFGAAGCTYDRWFAYMGDDDT-PLVPFPIVYNETAPDGMELY

***HaNPC1b***  VDVINFRVYEKFMLDAHASCSGVLVPQTGMPAINMMCG--NAVVCDAEAWFGFTGDTTVNPLAPVHVNF-HMWPNTEESM

***DmNPC1b***  VKYIDYRLTDDTVSKIYNSCIGIQHTQTGRPAMDLGCGSYNAKTCNYRRWYEFMGDVSG-DYVPFQINY-KWSEDAEEGS

***HsNPC1L1*** VVAYEAFYQHSFAEQSYDSCSRVRVPAAATLAVGTMCGVYGSALCNAQRWLNFQGDTGN-GLAPLDITFHLLEPGQAVGS

**250 260 270 280 290 300 310 320**

**....|....|....|....|....|....|....|....|....|....|....|....|....|....|....|....|**

***SgNPC1b***  N-----PDVLPCNQSYENDYA-CSCADCEESCPADITVIDTGETNEEFKIGEADGVLVIMAIIYGIFAIGFLISVIFVSQ

***HaNPC1b***  N-----VEALPCNETFGDDLP-CSCVDCVSTCP--VGTEPVVPDICTVLAVNCYGFSVGVVFFVISVIIFMVLAYKERTK

***DmNPC1b***  NEIYLDLSPLKCGESYEDSYA-CACIDCEESCP--LTDAPTGPDELWKIAG-LYGVTFILALIIACALSFFIFWG-----

***HsNPC1L1*** GIQPLNEGVARCNESQGDDVATCSCQDCAASCP-AIARPQALDSTFYLGQMPGSLVLIIILCSVFAVVTILLVGFRVAPA

**330 340 350 360 370 380 390 400**

**....|....|....|....|....|....|....|....|....|....|....|....|....|....|....|....|**

***SgNPC1b***  SDTAGGSPPSLIYKLFFGGIPLFQTTLSKLFRKLGLVFASYPGATICLTSWAVVGMSYGALSLIVTTDRIELWASPTSQS

***HaNPC1b***  QNKSADSKESGTGPNKTT------RLFQSMFAKIGGFSASNPVLVIMLTSWVTFGMIFGLAYLNLTSNPIELWSAPESRS

***DmNPC1b***  -AFGKTSAPSVCMPTLFG------EFFYHGFRIWGTFCAKHPVIVLALCSWAIAGLSFGIRYMTITTDPVELWAGEESQT

***HsNPC1L1*** RDKSKMVDPKKGTSLSDKLSFSTHTLLGQFFQGWGTWVASWPLTILVLSVIPVVALAAGLVFTELTTDPVELWSAPNSQA

**410 420 430 440 450 460 470 480**

**....|....|....|....|....|....|....|....|....|....|....|....|....|....|....|....|**

***SgNPC1b***  RIEKDYFDTHFGPFYRTEQVFIKAHGLDNIMYESGMQ-PVEFGPVFNKTFLLTVLDLQQQIIQIGA----GES-YELKNI

***HaNPC1b***  RQHLNYFNERFGPFYRPAQAFLRIDLDGFEANNVS------YGSAFRIEALEELVKLEDVIINIGR----EDGGVKLEDV

***DmNPC1b***  RIEKDYFDQHFGPFYRTNQMFVKAVNQTYFTHETSNG-VLNFGPAFEYNFLKEVFELQDSIMKLGM----ADN-EGLDKI

***HsNPC1L1*** RSEKAFHDQHFGPFFRTNQVILTAPNRSSYRYDSLLLGPKNFSGILDLDLLLELLELQERLRHLQVWSPEAQRNISLQDI

**490 500 510 520 530 540 550 560**

**....|....|....|....|....|....|....|....|....|....|....|....|....|....|....|....|**

***SgNPC1b***  CNAPLATG--EVKTEDCLVQSIWGYLKNNASLLE---------DDSYLSILLTCMQNN-----------VDFSCLGPYGG

***HaNPC1b***  CYAPLRQRGGEKRLDQCVSMSASSYLAGDRNDIN---------PNTYLTNIQNCINNH-----------YSFDCLASWGG

***DmNPC1b***  CYAPVLMAGETPTVDRCAIQSVYGYFQHDMDRFENSYVDSNNYTINYLNQLEDCLRVP-----------MMEDCFGTFGG

***HsNPC1L1*** CYAPLNPD--NTSLYDCCINSLLQYFQNNRTLLLLTANQTLMGQTSQVDWKDHFLYCANAPLTFKDGTALALSCMADYGA

**570 580 590 600 610 620 630 640**

**....|....|....|....|....|....|....|....|....|....|....|....|....|....|....|....|**

***SgNPC1b***  PIFAGLALGGFPEDANVNYSKYALSTGLSLTFLVNNHLD-KTDLQPALEWESKFIQFLKEWSETKMPSFMSIAFSAERSI

***HaNPC1b***  GAEPDLVFGGF------EPGNILSANTLLINFPIANFLL-EEDLQPVLEWELKFIEILHDYRDNWKQDFVHVAFSTERSI

***DmNPC1b***  PIEPGIAVGGMPKVAVGEDPDYMLATGLVLTFLGRNYND-ESKLEPNMKWEKLFVDFLRDYKS----DRLDIAYMAERSI

***HsNPC1L1*** PVFPFLAIGGYK------GKDYSEAEALIMTFSLNNYPAGDPRLAQAKLWEEAFLEEMRAFQR-RMAGMFQVTFMAERSL

**650 660 670 680 690 700 710 720**

**....|....|....|....|....|....|....|....|....|....|....|....|....|....|....|....|**

***SgNPC1b***  EDELARESQAEIATVVISYAVMFLYITLAIGRYRSSKTIQIDGKFTLGVSGIIIVLMSVLSSLGIFGYAGVATTLLTIEV

***HaNPC1b***  EDEIQRVSVAEAVPIVISYILMFIYVTLSLGNIRSLKTWFIDSKIMVAIGSIVVVILAIVCAMGALGFASVTLTLLAINV

***DmNPC1b***  QDAIVELSEGEVSTVVISYVVMFVYVAIALGHIRSCRGFLRESRIMLAIGGIVIVLASVVCSLGFWGYLDVTTTMLAIEV

***HsNPC1L1*** EDEINRTTAEDLPIFATSYIVIFLYISLALGSYSSWSRVMVDSKATLGLGGVAVVLGAVMAAMGFFSYLGIRSSLVILQV

Sterol-sensing domain

**730 740 750 760 770 780 790 800**

**....|....|....|....|....|....|....|....|....|....|....|....|....|....|....|....|**

***SgNPC1b***  IPFLVLAIGVDNIFILVQTHQRNP------------RQKTESHEEHVARTLGTVGPSMLLTSLSETCCFLIGALSNMPAV

***HaNPC1b***  IPFFVLSIGIDNVFLMVNTLHDVQGNLKSYDDYKEDFTFEKKRKFVFEKMLRQVGPSMFVSSVTQITCFAIGSIANFPAV

***DmNPC1b***  IPFLVLAVGVDNIFIMVHTYQRLD------------HSKFKTTHEAIGEAIGQVGPSILQTAGSEMACFAIGCISDMPAV

***HsNPC1L1*** VPFLVLSVGADNIFIFVLEYQRLP------------RRPGEPREVHIGRALGRVAPSMLLCSLSEAICFFLGALTPMPAV

**810 820 830 840 850 860 870 880**

**....|....|....|....|....|....|....|....|....|....|....|....|....|....|....|....|**

***SgNPC1b***  NTFALYASVATILNFIFQITCFVSIMTLDDKRQSVNRYDICCCVKSE-KAVDD-------TPSED-FLYLLFKSYYSKFL

***HaNPC1b***  VTFAIFASVSLSFLFVFQITTVVALLSIDYKRASSNRLDLFCCIQK--KVLDDENPLHSETPYKG--VTQRLMEPYSKFL

***DmNPC1b***  KTFAMYAAIAILLDFLLQITAFVALMAIDEKRYLDGRLDMLCCVKSGGKKINDEDGDGVDRPKEVGLLETLFKNFYSPFL

***HsNPC1L1*** RTFALTSGLAVILDFLLQMSAFVALLSLDSKRQEASRLDVCCCVKPQ---------------------------------

**890 900 910 920 930 940 950 960**

**....|....|....|....|....|....|....|....|....|....|....|....|....|....|....|....|**

***SgNPC1b***  LNRFVKIVVVLLFSFWLCSSLVLIPRIEAGLDQEIAVPTDSYVRSYFEYMKDVLSMGPNVYFVVKSGLNYSNENVQNLFC

***HaNPC1b***  LGFRVKVVVVIIFLAMVSISVMLIPELEIGLDQELALPKDSYVYEYLLAVANLIRMGPPVYFVVKGGLDFTNPIHQNTIC

***DmNPC1b***  LSKPVKVSVLLIFTVITCLSLMVTPSIEKGLDQEMSMPKNSHVVKYFRYMVDLLAMGAPVYWVLKPGLNYSEPLQQNLIC

***HsNPC1L1*** ----------------------ELPPPGQGEGLLLGFFQKAYAPFLLHWITRGVVVGAPVYFVTTLGYNFSSEAGMNAIC

**970 980 990 1000 1010 1020 1030 1040**

**....|....|....|....|....|....|....|....|....|....|....|....|....|....|....|....|**

***SgNPC1b***  GGLNCYADSLTTQITRASKLPDRSYITTSASSWLDDYFDWINSG-NCCQD--DKGTECSETP------------------

***HaNPC1b***  GGQLCNSDSLTTQIFLAAQHSNITYIAKSSNSWLDDFIDWSSLYGGCCKYNTTDGGFCESSSSEQECAFCEIPRSDYANG

***DmNPC1b***  GGVECNNNSLSVQLYTQAQYPEITSLARPASSWLDDYIDWLAIS-DCCKYNVTTGGFCSSNSKSEDCLPCERGFTENG--

***HsNPC1L1*** SSAGCNNFSFTQKIQYATEFPEQSYLAIPASSWVDDFIDWLTPS-SCCRLYISGPNKDKFCPSTVNSLNCLKNCMSITMG

**1050 1060 1070 1080 1090 1100 1110 1120**

**....|....|....|....|....|....|....|....|....|....|....|....|....|....|....|....|**

***SgNPC1b***  --RPTPKDFETYLPQFLLANPSQECAKGGHAAYAEGLNYVLNKDGLAIAMDSYFMTYHTVLRTSEEYTNALRSAREIASS

***HaNPC1b***  FLRPHVDAFETYIPFFLRDPPTEVCNKAGLASYSSAVNYVLNAEGRATVYDTNFMAYHSPLSTSKDYITAVDYGYRIASN

***DmNPC1b***  -LRPDAETFNKYIPYFLFDLPDAECAKAGRASYADAVIYTIDDVGMSTVQDSYFMQYSTTSTTSEEFYSQLREVRRISGE

***HsNPC1L1*** SVRPSVEQFHKYLPWFLNDRPNIKCPKGGLAAYSTSVNLTSDGQ----VLASRFMAYHKPLKNSQDYTEALRAARELAAN

**1130 1140 1150 1160 1170 1180 1190 1200**

**....|....|....|....|....|....|....|....|....|....|....|....|....|....|....|....|**

***SgNPC1b***  ITTMLQSYLRPTVNVTTENDTDSTNWFEVDNDTYISNVSNVEVFPYSVFYVFYEQYLTITGDTINSIGYSLSAVFIVSFI

***HaNPC1b***  ISAAIK----------------------------ANTGVDVEVFPYSLFYVFFEQYLTMWSDTFSSIGYCLIGALFFNLI

***DmNPC1b***  INAMFK----------------------------ENN-VDAEIFAYCVFYIYYEQYLTIWGDAMFSLGMSLVAIFLVTLL

***HsNPC1L1*** ITADLR--------------------------KVPGTDPAFEVFPYTITNVFYEQYLTILPEGLFMLSLCLVPTFAVSCL

Patched domain

**1210 1220 1230 1240 1250 1260 1270 1280**

**....|....|....|....|....|....|....|....|....|....|....|....|....|....|....|....|**

***SgNPC1b***  LMGFNIFSSVIILIMVTIIVTNLAAFMVWWDVPLNAVSLVNLVVGVGIAVEFCSHIIRAYTVSTLSGSNKRAADALTKIG

***HaNPC1b***  ASGFNVLTTFAVLLNTIMVVLNMMSVMYIWNIPLNAVSNVNLIVSIGISVEFCSHIAYAYSTSQRHG-REKVEEAIQKVG

***DmNPC1b***  ITGLDITSTFIVLFMVICILINMLGMMWAWSINLNAISLVNLVVCVGIGVEFVAHIVRSFKRAEGTA-QERARHSLNVTG

***HsNPC1L1*** LLGLDLRSGLLNLLSIVMILVDTVGFMALWGISYNAVSLINLVSAVGMSVEFVSHITRSFAISTKPTWLERAKEATISMG

**1290 1300 1310 1320 1330 1340 1350 1360**

**....|....|....|....|....|....|....|....|....|....|....|....|....|....|....|....|**

***SgNPC1b***  SSVLSGITLTKIVGIFVLAFAKTEIFQVFYFRMYLGIVLIGAAHGLIFLPVLLSYIGPNKNTNPTIEDITRST-------

***HaNPC1b***  ATIITGITFTNIP-IVVLAFSYTEIIEVFFFRMFLSLVVLGFLHGMIFFPVLLCYLDSLKRK------------------

***DmNPC1b***  SSVLSGITLTKFAGIVVLGFSNSQIFQVFYFRMYLGIVLIGAAHGLILLPVLLSLLGPPQKLARSSGAEPTASITITTN-

***HsNPC1L1*** SAVFAGVAMTNLPGILVLGLAKAQLIQIFFFRLNLLITLLGLLHGLVFLPVILSYVGPDVNPALALEQKRAEEAVAAVMV

**1370 1380 1390 1400**

**....|....|....|....|....|....|....|....|...**

***SgNPC1b***  -------------------------------------------

***HaNPC1b***  -------------------------------------------

***DmNPC1b***  -------------------------------------------

***HsNPC1L1*** ASCPNHPSRVSTADNIYVNHSFEGSIKGAGAISNFLPNNGRQF

**
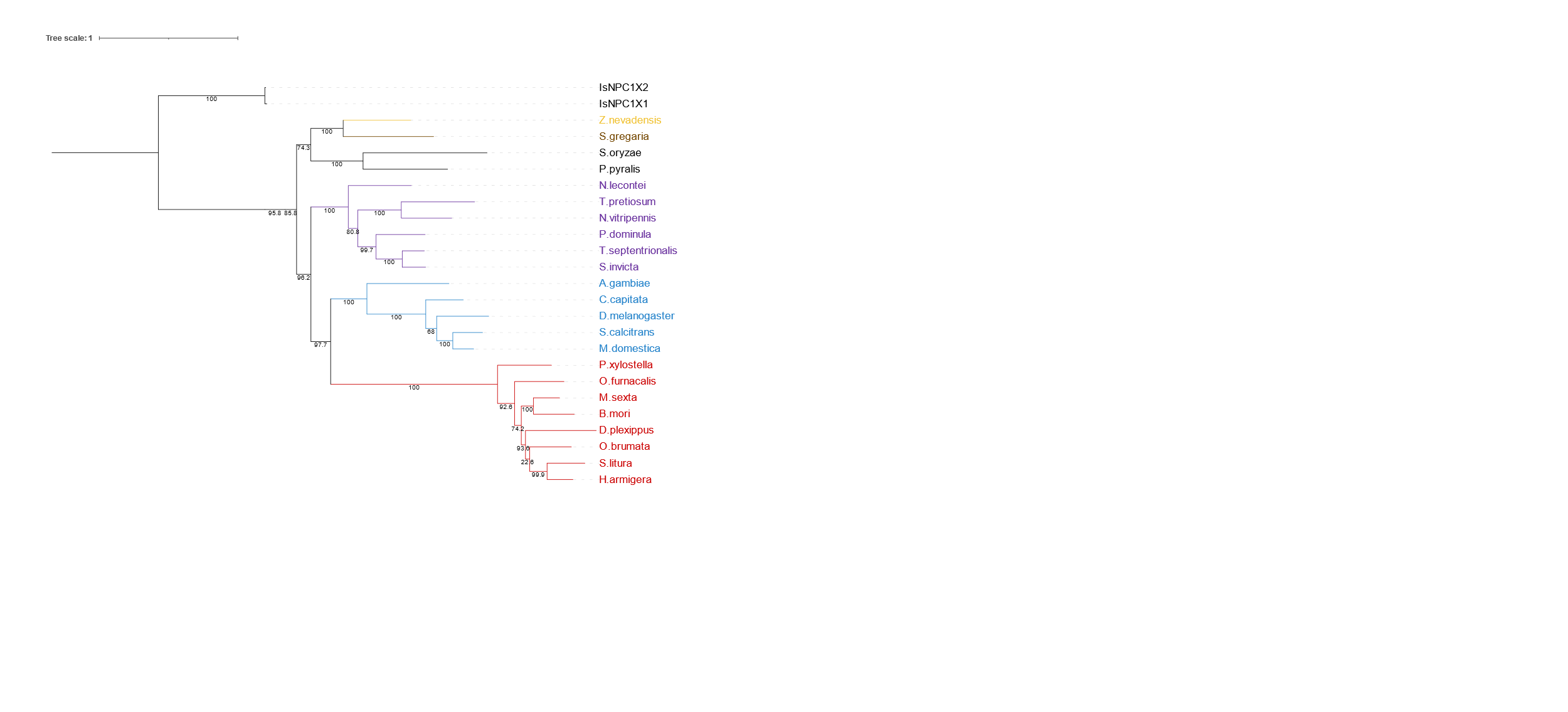
**

**Supplementary Figure 9**. **Phylogenetic analysis of NPC1b proteins in various insect species.** Phylogenetic relationships of NPC1b proteins obtained by Maximum Likelihood method. The branch lengths are indicated at each branch and is expressed as the amino acid substitution rate per site. The branch length scale is indicated by the scale bar in the top left corner. The node support (%) is indicated at each node. Different insect orders are indicated with distinct colors (Lepidoptera: red, Diptera: blue, Isoptera: yellow, Orthoptera: brown, Coleoptera: black, Hymenoptera: purple). Two NPC1 sequences from the arachnid *Ixodes scapularis* were used as an outgroup to root the tree (IsNPC1X1 and IsNPC1X2).


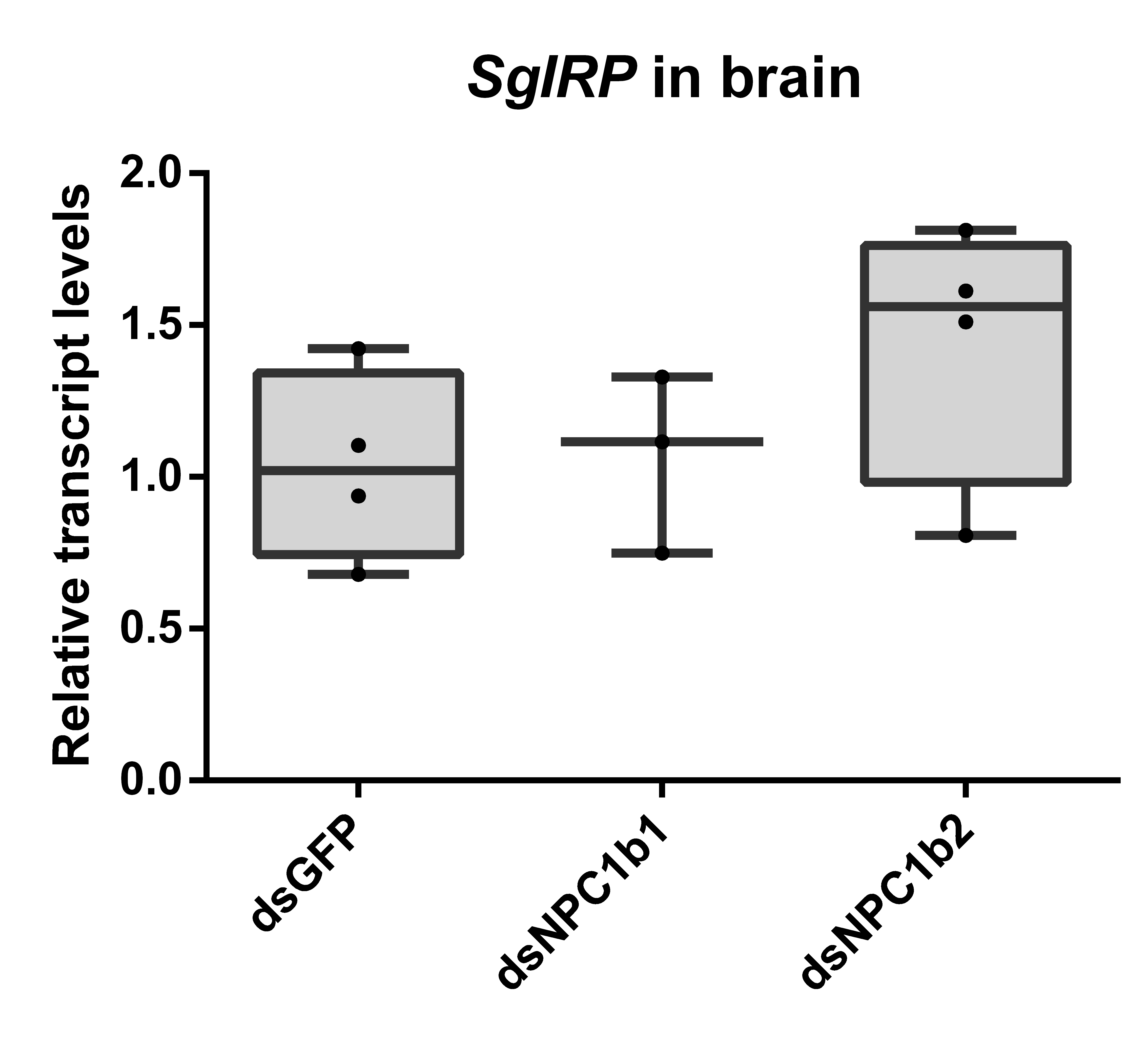


**Supplementary Figure 10.** **Insulin-related peptide transcript abundance in the brain upon *SgNPC1b* knockdown.** Relative transcript levels of insulin-related peptide (*SgIRP)* in the brain of *dsGFP* and *dsNPC1b* injected animals measured by RT-qPCR (*dsGFP*: n=4, *dsNPC1b1*: n=3, *dsNPC1b2*: n=4). Data are represented by boxplots containing the upper and lower quartile, while the whiskers indicate the minimum and maximum. The median is indicated by the grey line in the center of the box. A *SgNPC1b* knockdown did not significantly affect *SgIRP* transcript levels in the brain (one-way ANOVA with Dunnett’s post-hoc test).

## Supplementary Tables

**Supplementary Table 1. Annotated mapped midgut transcripts.**

See file “Suppl. Table 1_ Midgut_Transcriptome_Annotated.xlsx”

**Supplementary Table 2.** List of TMM normalized counts per million (CPM) of all samples. Sample S1-S6: 10 min after feeding, sample S7-S12: 2 h after feeding, sample S13-S18: 24 h after feeding.

See file “Suppl. Table 2_TMM_Normalized_CountTable_allsamples.xlsx”

**Supplementary Table 3.** Oligonucleotide sequences for primers used in dsRNA construct design. Underlined sequences are the T7 promoter sequences. *SgNPC1b1* and *SgNPC1b2* refer to two non-overlapping dsRNA constructs targeting *SgNPC1b*.

| **Target genes** | **Forward primer** | **Reverse primer** | |  |
| --- | --- | --- | --- | --- |
| *SgVAHa1* | 5’- TAATACGACTCACTATAGGGCTGACAGTGGGAA  CCCTGAT-3’ | | 5’-TAATACGACTCACTATAGGGTTGCAGTGCTTGAGT  CATCC-3’ | |
| *SgNPC1b 1* | 5’-TAATACGACTCACTATAGGGTCGGAGCTGGTGA  AAGCTA-3’ | | 5’-TAATACGACTCACTATAGGGGTGCAACATGTTCC  TCATGG-3’ | |
| *SgNPC1b 2* | 5’-TAATACGACTCACTATAGGGCGCATTGAGGCTG  GATTAGA-3’ | | 5’-TAATACGACTCACTATAGGGTCAGAGCATTTGTG  TACTCCTC-3’ | |
| *GFP* | 5’-TAATACGACTCACTATAGGGAGAAAGGTGATGC  TACATACGGAA-3’ | | 5’-TAATACGACTCACTATAGGGAGAATCCCAGCAGC  AGTTACAAAC-3’ | |

Abbreviations*: VAHa1= H^+^ V-ATPase subunit-a isoform 1, NPC1b =niemann-pick C1 protein isoform b, GFP = green fluorescent protein*

**Supplementary Table 4**. Oligonucleotide sequences for primers used in RT-qPCR.

| **Reference genes** | **Forward primer** | **Reverse primer** |
| --- | --- | --- |
| *SgGAPDH* | 5’-CGCTACAAGAAGCTTAAGAGGTCAT-3’ | 5’-CCTACGGCGCACTCTGTTG-3’ |
| *SgRP49* | 5’-GTCTGATGACAACAGTGCAT-3’ | 5’-GTCCATCACGCCACAACTTTC-3’ |
|  |  |  |
| **Target genes** | Forward primer | Reverse primer |
| *SgVAHa1* | 5’-TATTACTGTCGCGTCGCATC-3’ | 5’-TCTTCTCGCTCCGAAACATC-3’ |
| *SgNPC1b* | 5’-CGGTGAATGTTACGATGACG -3’ | 5’-TTCTTGGTCCACCAGAAAGG-3’ |

Abbreviations: *SgGAPDH = Glyceraldehyde 3-phosphate dehydrogenase, RP49*=*ribosomal protein 49*, *VAHa1= H^+^ V-ATPase subunit-a isoform 1, NPC1b =niemann-pick C1 protein isoform b*

**Supplementary Table 5**. Predicted digestive enzymes with annotation in the *S. gregaria* midgut transcriptome.

| **Predicted Identity** | **# Transcripts** |
| --- | --- |
| **Protein digestion** |  |
| Endopeptidase activity |  |
| Serine protease |  |
| Trypsin | 84 |
| Chymotrypsin | 22 |
| Elastase | 6 |
| Cysteine protease | 9 |
| Aspartic protease | 3 |
| Metalloprotease | 8 |
| Exopeptidase activity |  |
| Aminopeptidase | 47 |
| Carboxypeptidase | 32 |
| Dipeptidase | 3 |
| **Carbohydrate digestion** |  |
| α-Amylase | 6 |
| α-Glucosidase | 20 |
| β-Glucosidase | 44 |
| Cellulase | 3 |
| **Lipid digestion** |  |
| Lipase | 22 |
| Phospholipase | 13 |
| Sterol dehydrogenase/reductase | 29 |
| **Nucleases** |  |
| DNase | 4 |
| RNase | 38 |
| Nucleotidase | 14 |
| Nucleosidase | 1 |

**Supplementary Table 6.** Summary of Pfam annotations of predicted nutrient transporters and their substrates present in the *S. gregaria* midgut transcriptome.

| **Nutrient** | **Pfam** | **# Transcripts** | **Description** |
| --- | --- | --- | --- |
| **Amino acids** | PF01490 | 17 | Solute carrier protein 36 |
|  | PF00324 | 12 | Cationic amino acid transporter (SLC7) |
|  | PF00209 | 7 | Sodium-coupled amino acid transporter (iNAT-SLC6) |
|  | PF10149 | 2 | Solute carrier protein 38 |
|  | PF00854 | 2 | Oligopeptide transporter (SLC15) |
| **Carbohydrates** | PF00083 | 53 | Facilitated hexose and polyol transporters (GLUT) |
|  | PF00474 | 3 | Sodium-glucose co-transporters (SGLT) |
| **Lipids** | PF00501 | 24 | Fatty acid transport protein (FATP) |
|  | PF00061 | 15 | Fatty acid binding protein (FABP) |
|  | PF01130 | 7 | Scavenger receptors class B type I (CD36) |
|  | PF00108 | 7 | Sterol carrier protein-x (SCPx) |
|  | PF02036 | 4 | Sterol carrier protein-2 (SCP2) |
|  | PF02221 | 4 | Niemann-Pick C2 (NPC2) |
|  | PF16414 | 2 | Niemann-Pick C1 (NPC1) |

**Supplementary Table 7**: Summary of Pfam annotations of proteins with important midgut-associated functions, outside of digestive enzymes and nutrient transporters, present in the *S. gregaria* midgut transcriptome.

| **Functional category** | **Pfam** | **# Transcripts** | **Description** |
| --- | --- | --- | --- |
| **Peritrophic membrane** | PF01607 | 59 | Chitin binding peritrophin A domain |
|  | PF00379 | 11 | Chitin binding domain |
|  | PF00704 | 13 | Endochitinase |
| **ATP-binding cassette (ABC) transporters** | PF00005 | 55 | ABC transporter NBD |
|  | PF00664 | 36 | ABC transporter TMD |
| **Transmembrane proton pump** | PF02874 | 4 | H+ V-ATPase V1 subunit-A |
|  | PF00137 | 4 | H+ V-ATPase V1 subunit-C |
|  | PF01813 | 2 | H+ V-ATPase V1 subunit-D |
|  | PF01991 | 1 | H+ V-ATPase V1 subunit-E |
|  | PF01990 | 1 | H+ V-ATPase V1 subunit-F |
|  | PF03179 | 1 | H+ V-ATPase V1 subunit-G |
|  | PF11698 | 1 | H+ V-ATPase V1 subunit-H |
|  | PF01496 | 3 | H+ V-ATPase V0 subunit-a |
| **Detoxification** | PF00067 | 132 | CYP450 |
|  | PF00201 | 63 | Uridine diphosphateglucuronosyltransferase (UGT) |
|  | PF02798/13417 | 59 | Glutathione S-transferase (GST) |
|  | PF00135 | 123 | Carboxylesterase (CE) |
| **Other** | PF00230 | 4 | Aquaporin |
|  | PF00999 | 4 | Sodium/hydrogen exchanger |
|  | PF00689/00690 | 1 | Sodium/potassium V-ATPase alpha |
|  | PF00287 | 3 | Sodium/potassium V-ATPase beta |
|  | PF00955 | 1 | Anion exchanger |

**Supplementary Table 8.** Upregulated transcripts 2 hours after feeding versus 24 hours after feeding.

See file “Suppl. Table 8_Upregulated_2 h_Transcripts_Annotated”

**Supplementary Table 9.** Downregulated transcripts 2 hours after feeding versus 24 hours after feeding.

See file “Suppl. Table 9_Downregulated_2 h_Transcripts_Annotated.xlsx”

**Supplementary Table 10.** List of all accession numbers of H^+^ V-ATPase 116 kDa subunit-a protein sequences used for phylogenetic analysis. A sequence from *Daphnia magna* (accession number JAN07249.1) was used as an outgroup for phylogenetic analysis.

| **Species** | **Order** | **Accession number** |
| --- | --- | --- |
| *Aethina tumida* | Coleoptera | [XP_019865195.1](https://www.ncbi.nlm.nih.gov/protein/XP_019865195.1?report=genbank&log$=prottop&blast_rank=4&RID=KS936M7H013) |
| *Agrilus planipennis* | Coleoptera | XP_025831982 |
| *Nicrophorus vespilloides* | Coleoptera | XP_017778558 |
| *Photinus pyralis* | Coleoptera | [XP_031337487](https://www.ncbi.nlm.nih.gov/protein/XP_031337487?report=genbank&log$=taxrep&RID=KS9NGEWS016) |
| *Tribolium castaneum* | Coleoptera | [XP_008200952.1](https://www.ncbi.nlm.nih.gov/protein/XP_008200952.1?report=genbank&log$=prottop&blast_rank=3&RID=KS936M7H013) |
| *Aedes aegypti* | Diptera | [XP_001657344.1](https://www.ncbi.nlm.nih.gov/protein/XP_001657344.1?report=genbank&log$=prottop&blast_rank=33&RID=KS2DJ7DV01R) |
| *Aedes albopictus* | Diptera | [XP_019546778.1](https://www.ncbi.nlm.nih.gov/protein/XP_019546778.1?report=genbank&log$=prottop&blast_rank=37&RID=KS2DJ7DV01R) |
| *Anopheles gambiae* | Diptera | [CAD27758](https://www.ncbi.nlm.nih.gov/protein/CAD27758?report=genbank&log$=taxrep&RID=KS2DJ7DV01R) |
| *Batrocera dorsalis* | Diptera | [XP_011211500.1](https://www.ncbi.nlm.nih.gov/protein/XP_011211500.1?report=genbank&log$=prottop&blast_rank=38&RID=KS850K21016) |
| *Culex pipiens* | Diptera | [XP_039445164.1](https://www.ncbi.nlm.nih.gov/protein/XP_039445164.1?report=genbank&log$=prottop&blast_rank=29&RID=KS2DJ7DV01R) |
| *Culex quinquefasciatus* | Diptera | [XP_038115263](https://www.ncbi.nlm.nih.gov/protein/XP_038115263?report=genbank&log$=taxrep&RID=KS2DJ7DV01R) |
| *Drosophila melanogaster* | Diptera | NP_650720.1 |
| *Hermetia illucens* | Diptera | [XP_037913625.1](https://www.ncbi.nlm.nih.gov/protein/XP_037913625.1?report=genbank&log$=prottop&blast_rank=94&RID=KS2DJ7DV01R) |
| *Musca domestica* | Diptera | [XP_005182534.1](https://www.ncbi.nlm.nih.gov/protein/XP_005182534.1?report=genbank&log$=prottop&blast_rank=28&RID=KS850K21016) |
| *Stomoxys calcitrans* | Diptera | [XP_013114901.1](https://www.ncbi.nlm.nih.gov/protein/XP_013114901.1?report=genbank&log$=prottop&blast_rank=27&RID=KS850K21016) |
| *Adelges cooleyi* | Hemiptera | XP_050419890 |
| *Cinara cedri* | Hemiptera | [VVC33897](https://www.ncbi.nlm.nih.gov/protein/VVC33897?report=genbank&log$=taxrep&RID=KS9NGEWS016) |
| *Daktulosphaira vitifoliae* | Hemiptera | [XP_050538204.1](https://www.ncbi.nlm.nih.gov/protein/XP_050538204.1?report=genbank&log$=prottop&blast_rank=63&RID=KS2DJ7DV01R) |
| *Homalodisca vitripennis* | Hemiptera | [XP_046682460.1](https://www.ncbi.nlm.nih.gov/protein/XP_046682460.1?report=genbank&log$=prottop&blast_rank=18&RID=KS2DJ7DV01R) |
| *Nilaparvata lugens* | Hemiptera | [XP_022201975.1](https://www.ncbi.nlm.nih.gov/protein/XP_022201975.1?report=genbank&log$=prottop&blast_rank=20&RID=KS2DJ7DV01R) |
| *Sipha flava* | Hemiptera | [XP_025423815.1](https://www.ncbi.nlm.nih.gov/protein/XP_025423815.1?report=genbank&log$=prottop&blast_rank=81&RID=KS2DJ7DV01R) |
| *Belonocnema kinseyi* | Hymenoptera | XP_033214483 |
| *Diprion similis* | Hymenoptera | XP_046737475 |
| *Neodiprion fabricii* | Hymenoptera | [XP_046411765](https://www.ncbi.nlm.nih.gov/protein/XP_046411765?report=genbank&log$=taxrep&RID=KS9NGEWS016) |
| *Cryptotermes secundus* | Isoptera | XP_023722813.1 |
| *Zootermopsis nevadensis* | Isoptera | [XP_021940961](https://www.ncbi.nlm.nih.gov/protein/XP_021940961?report=genbank&log$=taxrep&RID=KS9NGEWS016) |
| *Bombyx mori* | Lepidoptera | [XP_004931128.1](https://www.ncbi.nlm.nih.gov/protein/XP_004931128.1?report=genbank&log$=prottop&blast_rank=60&RID=KS2DJ7DV01R) |
| *Helicoverpa armigera* | Lepidoptera | [XP_021200676.2](https://www.ncbi.nlm.nih.gov/protein/XP_021200676.2?report=genbank&log$=prottop&blast_rank=65&RID=KS2DJ7DV01R) |
| *Leptidea sinapsis* | Lepidoptera | [XP_050669605.1](https://www.ncbi.nlm.nih.gov/protein/XP_050669605.1?report=genbank&log$=prottop&blast_rank=51&RID=KS2DJ7DV01R) |
| *Manduca sexta* | Lepidoptera | [XP_030029557.1](https://www.ncbi.nlm.nih.gov/protein/XP_030029557.1?report=genbank&log$=prottop&blast_rank=35&RID=KS2DJ7DV01R) |
| *Pieris rapae* | Lepidoptera | [XP_022118568.1](https://www.ncbi.nlm.nih.gov/protein/XP_022118568.1?report=genbank&log$=prottop&blast_rank=45&RID=KS2DJ7DV01R) |
| *Plutella xylostella* | Lepidoptera | [XP_037965097.2](https://www.ncbi.nlm.nih.gov/protein/XP_037965097.2?report=genbank&log$=prottop&blast_rank=95&RID=KS2DJ7DV01R) |
| *Spodoptera frugiperda* | Lepidoptera | [XP_050562224.1](https://www.ncbi.nlm.nih.gov/protein/XP_050562224.1?report=genbank&log$=prottop&blast_rank=87&RID=KS2DJ7DV01R) |
| *Trichoplusia ni* | Lepidoptera | [XP_026737260.1](https://www.ncbi.nlm.nih.gov/protein/XP_026737260.1?report=genbank&log$=prottop&blast_rank=79&RID=KS2DJ7DV01R) |
| *Vanessa atalanta* | Lepidoptera | [XP_047528562.1](https://www.ncbi.nlm.nih.gov/protein/XP_047528562.1?report=genbank&log$=prottop&blast_rank=44&RID=KS2DJ7DV01R) |
| *Ischnura elegans* | Odonata | [XP_046398900.1](https://www.ncbi.nlm.nih.gov/protein/XP_046398900.1?report=genbank&log$=prottop&blast_rank=12&RID=KSAAFGHG013) |
| *Schistocerca americana* | Orthoptera | XP_046999637.1 |
| *Schistocerca gregaria* | Orthoptera | XP_049832128.1 |
| *Ctenocephalides felis* | Siphonaptera | [XP_026476806.1](https://www.ncbi.nlm.nih.gov/protein/XP_026476806.1?report=genbank&log$=prottop&blast_rank=17&RID=KS2DJ7DV01R) |
| *Frankliniella occidentalis* | Thysanoptera | XP_026280156.1 |
| *Thrips palmi* | Thysanoptera | [XP_034233898.1](https://www.ncbi.nlm.nih.gov/protein/XP_034233898.1?report=genbank&log$=prottop&blast_rank=14&RID=KSAAFGHG013) |

**Supplementary Table 11.** List of all GenBank accession numbers of NPC1b protein sequences used for phylogenetic analysis. As an outgroup 2 NPC1 sequences from *Ixodes scapularis* were used (IsNPC1X1, accession number XP_002435857.2 and IsNPC1X2, accession number XP_029829620.1)

| **Species** | **Order** | **Accession number** |
| --- | --- | --- |
| *Sitophilus oryzae* | Coleoptera | XP_030749451.1 |
| *Photinus pyralis* | Coleoptera | XP_031329506.1 |
| *Tribolium castaneum* | Coleoptera | XP_008199197.1 |
| *Anopheles gambiae* | Diptera | EAA06340.6 |
| *Ceratitis capitata* | Diptera | XP_004523941.1 |
| *Drosophila melanogaster* | Diptera | NP_608417.2 |
| *Musca domestica* | Diptera | XP_011290675.1 |
| *Stomoxys calcitrans* | Diptera | XP_013112245.1 |
| *Nasonia vitripennis* | Hymenoptera | XP_008208782.1 |
| *Neodiprion lecontei* | Hymenoptera | XP_015518929.1 |
| *Polistes dominula* | Hymenoptera | XP_015180568.1 |
| *Solenopsis invicta* | Hymenoptera | XP_011166446.1 |
| *Trachymyrmex septentrionalis* | Hymenoptera | KYN32399.1 |
| *Trichogramma pretiosum* | Hymenoptera | XP_023316634.1 |
| *Zootermopsis nevadensis* | Isoptera | XP_021915666 |
| *Helicoverpa armigera* | Lepidoptera | QEP99645.1 |
| *Operophtera brumata* | Lepidoptera | KOB70440.1 |
| *Plutella xylostella* | Lepidoptera | AYA73999.1 |
| *Spodoptera litura* | Lepidoptera | XP_022826606.1 |
| *Manduca sexta* | Lepidoptera | XP_030023820.2 |
| *Bombyx mori* | Lepidoptera | XP_037873251.1 |
| *Danaus plexippus* | Lepidoptera | XP_032518509.1 |
| *Ostrinia furnacalis* | Lepidoptera | XP_028178908.1 |
| *Schistocerca gregaria* | Orthoptera | [XP_049852647.1](https://www.ncbi.nlm.nih.gov/protein/XP_049852647.1?report=genbank&log$=prottop&blast_rank=1&RID=WJER79KS01N) |
